# Nuclear interacting SET domain protein 1 inactivation impairs GATA1-regulated erythroid differentiation and causes erythroleukemia

**DOI:** 10.1038/s41467-020-16179-8

**Published:** 2020-06-12

**Authors:** Katharina Leonards, Marwa Almosailleakh, Samantha Tauchmann, Frederik Otzen Bagger, Cécile Thirant, Sabine Juge, Thomas Bock, Hélène Méreau, Matheus F. Bezerra, Alexandar Tzankov, Robert Ivanek, Régine Losson, Antoine H. F. M. Peters, Thomas Mercher, Juerg Schwaller

**Affiliations:** 1 University Children’s Hospital Basel, Basel, Switzerland; 2 Department of Biomedicine, University of Basel, 4031 Basel, Switzerland; 3 Swiss Institute of Bioinfomatics, 4031 Basel, Switzerland; 4 Genomic Medicine, Righospitalet, University of Copenhagen, 2100 Copenhagen, Denmark; 5 INSERM U1170, Equipe Labellisée Ligue Contre le Cancer, Gustave Roussy Institute, Université Paris Diderot, Université Paris-Sud, Villejuif, 94800 France; 6 Proteomics Core Facility, Biozentrum University of Basel, Basel, Switzerland; 7 Aggeu Magalhães Institute, Oswaldo Cruz Foundation, Recife, Brazil; 8 Institute for Pathology, University Hospital Basel, 4031 Basel, Switzerland; 9 Institute de Génétique et de Biologie Moléculaire et Cellulaire (I.G.B.M.C.), CNRS/INSERM Université de Strasbourg, BP10142, 67404 Illkirch Cedex, France; 10 Friedrich Miescher Institute for Biomedical Research, 4058 Basel, Switzerland; 11 Faculty of Sciences, University of Basel, 4056 Basel, Switzerland

**Keywords:** Acute myeloid leukaemia, Molecular medicine

## Abstract

The nuclear receptor binding SET domain protein 1 (NSD1) is recurrently mutated in human cancers including acute leukemia. We show that NSD1 knockdown alters erythroid clonogenic growth of human CD34^+^ hematopoietic cells. Ablation of *Nsd1* in the hematopoietic system of mice induces a transplantable erythroleukemia. In vitro differentiation of *Nsd1*^*−/−*^ erythroblasts is majorly impaired despite abundant expression of GATA1, the transcriptional master regulator of erythropoiesis, and associated with an impaired activation of GATA1-induced targets. Retroviral expression of wildtype NSD1, but not a catalytically-inactive NSD1^N1918Q^ SET-domain mutant induces terminal maturation of *Nsd1*^*−/−*^ erythroblasts. Despite similar GATA1 protein levels, exogenous NSD1 but not NSD^N1918Q^ significantly increases the occupancy of GATA1 at target genes and their expression. Notably, exogenous NSD1 reduces the association of GATA1 with the co-repressor SKI, and knockdown of SKI induces differentiation of *Nsd1*^*−/−*^ erythroblasts. Collectively, we identify the NSD1 methyltransferase as a regulator of GATA1-controlled erythroid differentiation and leukemogenesis.

## Abstract (web summary)

Loss of function mutations of NSD1 occur in blood cancers. Here, the authors report that NSD1 loss blocks erythroid differentiation which leads to an erythroleukemia-like disease in mice by impairing GATA1-induced target gene activation.

## Introduction

Steady-state erythropoiesis is primarily controlled by erythropoietin (EPO) and other hormones including stem cell factor and glucocorticoids. Different pathways translate external signals to the activation of transcription factors and co-regulators that drive expression programs that define erythroid identity^1^. Erythroid differentiation is mainly regulated by a relatively small number of transcriptional regulators, including GATA-1, SCL/TAL1, LMO2, LDB1, KLF1, and GFI1b, that dynamically form multiprotein complexes. However, it remains poorly understood how distinct complexes interact and activate or repress specific gene expression programs^2^.

The best studied erythroid transcription factor is the GATA1 zinc-finger protein. GATA1 was shown to activate its target genes by complexing with SCL/TAL1, the bHLH protein E2A, and the LIM domain containing factors LMO2 and LDB1. GATA1-mediated repression was proposed to be executed by complexes containing FOG1, GFI1b, and/or Polycomb repressive complex 2 (PRC2) proteins^2,3^. Inactivation studies in mice revealed that GATA1 is an essential master regulator of erythropoiesis as *Gata1*-null embryos died in utero from anemia^4^. Moreover, some adult female mice that are heterozygous for the targeted disruption of the X chromosome-linked *Gata1* promoter region displayed reduced *Gata1* gene expression (*Gata1*^*1.05/X*^ allele) and developed an early onset erythroleukemia-like disease^5^. This mouse model suggested that reduced Gata1 activity contributes to leukemogenesis by preventing proper erythroid differentiation. Acute erythroleukemia is a rare form of human acute myeloid leukemia (AML) generally associated with poor outcome^6^. Recent studies started to unravel the genetic AEL landscape but the molecular mechanisms that control the erythroid identity of the tumor cells remain poorly understood^7^.

The nuclear receptor SET domain protein 1 (NSD1) histone methyltransferase was identified as a protein interacting with several nuclear receptors^8,9^. Mono- and di-methylation of histone 3 lysine 36 (H3K36) and lysine 168 of linker histone 1.5 have been proposed to be the major cellular NSD1 substrates^10,11^. Multiple studies suggest that *NSD1* can act as a tumor suppressor gene. First, the *NSD1* gene locus is subject to recurrent putative loss-of-function mutations in hematological malignancies and solid cancers^12–16^. Second, the CpG island promoter of the *NSD1* locus has also been reported to be frequently hyper-methylated in certain human cancers, thereby epigenetically silencing the allele^17,18^. Third, heterozygous germline point mutations in *NSD1* are the molecular correlate for SOTOS, an overgrowth syndrome with learning disabilities and increased cancer risk^19,20^. Finally, *NSD1* was identified as putative cancer predisposition gene mediated by rare germline variants and somatic loss-of-heterozygosity (LOH)^21^. However, the mechanism of how NSD1 protects different cell types from malignant transformation remains unknown.

We study the role of NSD1 in steady-state hematopoiesis and leukemia. We observe that reduced *NSD1* expression alters the clonogenic growth of erythroid progenitor cells derived from human CD34^+^ hematopoietic cells. Targeted *Nsd1* gene inactivation during late fetal hematopoiesis in mice leads to malignant accumulation of erythroblasts phenocopying human acute erythroleukemia. Complementation experiments reveal that the NSD1-SET domain is critical for in vitro erythroblast terminal differentiation. In addition, our work suggests that NSD1 controls target gene activation by the erythroid master regulator GATA1, most likely through regulated association with the transcriptional co-repressor SKI. Collectively, we identify NSD1 as a co-regulator of GATA1-controlled terminal erythroid maturation and leukemogenesis.

## Results

### *NSD1* knockdown in human CD34^+^ hematopoietic cells

**Fig. 1:**
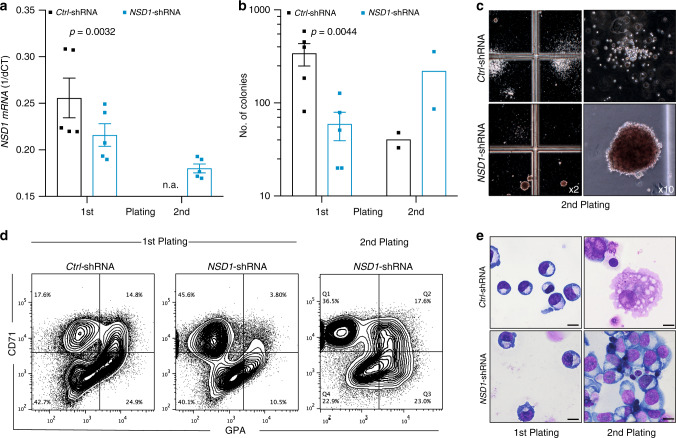
*NSD1* knockdown alters clonogenic erythroid differentiation of human CD34^+^ hematopoietic cells. **a** Relative *NSD1* mRNA expression (1/dCt) in peripheral blood CD34^+^ cells transduced with *pLKO.1* expressing control shRNA (*Ctrl*) or *NSD1* shRNA *(*#372) harvested from the first and second plating in growth-factor-containing MC (H4434). Bars represent average relative expression normalized to (*n* = 5 per group). **b** Numbers of colonies formed by 4 × 10^4^ peripheral CD34^+^ cells transduced with *pLKO.1* expressing control shRNA (*Ctrl)* or *NSD1* shRNA in the first plate (*n* = 5) and upon replating (*n* = 2) in growth-factor-containing MC (H4434). **c** Representative images of colonies formed in MC (H4434) by 4 × 10^4^ peripheral CD34^+^ cells transduced with *pLKO.1* expressing control or *NSD1* shRNA. **d** Flow cytometric analysis of cells harvested from the first and second plating in MC (H4434) revealed accumulation of CD71^high^ and glycoprotein A (GPA)^−^ cells upon replating. The plots represent one out of three independent experiments. **e** Representative images of Wright Giemsa-stained cytospin preparations from control shRNA (*Ctrl)* or *NSD1* shRNA-expressing CD34^+^ cells harvested from the MC (H4434) cultures after the first and second plating, illustrating the overall predominance of cells with erythroblast morphology upon replating (one out of three experiments) (×1000, the size bar = 10 μM). Values are presented as individual points, bar graphs represent the mean value of biological replicates, error bars as standard error of the mean. Statistical significances in **a**, **b** was tested with paired two-tailed *t*-test.

To address the role of NSD1 in hematopoiesis, we first optimized lentiviral shRNA-mediated knockdown in human CD34^+^ hematopoietic cells (Supplementary Fig. 1a–d). We identified three NSD1 shRNA that reduced the numbers of colonies grown in methylcellulose (MC) containing growth factors including EPO (Fig. 1a, b, Supplementary Fig. 1a, b). Interestingly, whereas very few colonies were generated upon replating of *Ctrl*-shRNA-transduced cells, cells transduced with *NSD1-*shRNA “372” or “353” formed abundant relatively dense reddish colonies (Fig. 1b, c, Supplementary Fig. 1c). These colonies were mostly composed of CD45^low^ cells expressing the transferrin receptor (CD71) and glycophorin-A (GPA) presenting with a proerythroblast-like morphology (Fig. 1d, e, Supplementary Fig. 1d). The cells could however not be further expanded in MC or in liquid cultures. Very similar results were obtained with human cord blood-derived cells (Supplementary Fig. 1e–h). Collectively, these data suggest that NSD1 regulates clonogenic erythroid differentiation of fetal and adult human CD34^+^ hematopoietic cells in vitro.

### Ablation of *Nsd1* induces erythroleukemia in mice

**Fig. 2:**
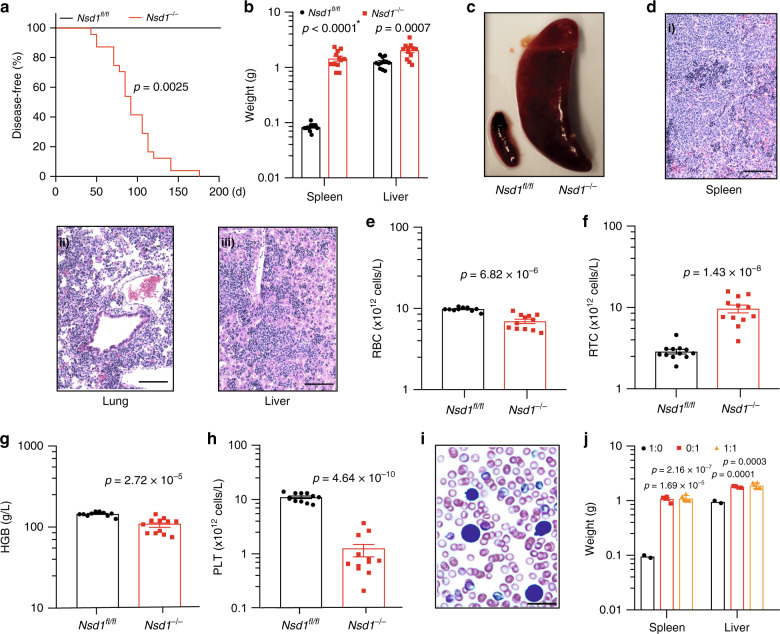
Hematopoietic ablation of *Nsd1* leads to a fully penetrant and transplantable leukemia-like disease in mice. **a** Kaplan Meier plot of disease-free survival of *Nsd1*^*fl/fl*^ (*n* = 12, black line) and *Nsd1*^*−/−*^ (*n* = 24; red line) mice. Median survival of *Nsd1*^*−/−*^ mice was 91 days; *Nsd1*^*fl/fl*^ mice did not develop any disease. **b** Spleen and liver weight of *Nsd1*^*fl/fl*^ and symptomatic *Nsd1*^*−/−*^ mice in grams (*n* = 12 per group) (* indicates a *p* value smaller than 1 × 10^−15^). **c** Representative image of spleens of *Nsd1*^*fl/fl*^ (left) and diseased *Nsd1*^*−/−*^ (right) mice. **d** Representative images (from 1 out of 24 mice) of HE-stained sections (×200, size bars = 50 μm) of (i) spleen, (ii) lung, and (iii) liver of diseased *Nsd1*^*−/−*^ mice showing significant cell infiltrations in all organs. **e** Peripheral red blood cell counts (RBC, ×10^12^ cells/l), **f** reticulocyte counts (RTC, ×10^12^ cells/l), **g** hemoglobin levels (HGB, g/l), and **h** platelet counts (PLT, ×10^12^ cells/l) in diseased *Nsd1*^*−/−*^ compared to *Nsd1*^*fl/fl*^ littermate controls (*n* = 12 per group). **i** Representative image of a Wright Giemsa-stained peripheral blood smear of a symptomatic *Nsd1*^*−/−*^ mouse with the presence of an erythroblast (from 1 out of 24 mice, ×600, the size bar = 10 μM). **j** Spleen and liver weight in grams of WT mice transplanted with whole BM from diseased *Nsd1*^*−/−*^ mice (red bars) (*n* = 4) or *Nsd1*^*fl/fl*^ littermate controls (black bars) (*n* = 2) or in a 1:1 mixture of *Nsd1*^*−/−*^ (CD45.2) and B6.SJL (CD45.1) cells (orange bars) (*n* = 6). Values are presented as individual points, bar graphs represent the mean value of biological replicates, error bars as standard error of the mean. Statistical significances in **a** was tested with log-rank Mantel Cox test, and in **b**, **e**–**h**, **j** with an unpaired two-tailed *t*-test.

To address its role in steady-state hematopoiesis, we inactivated *Nsd1* in mice^22^. *Nsd1*^*fl/fl*^*;Vav1-iCre*^*tg/+*^ transgenic mice (here referred as *Nsd1*^*−/−*^) efficiently excised both alleles in cells from different lineages leading to almost undetectable levels of *Nsd1 exon 5* mRNA and protein expression (Supplementary Fig. 2a–g). At the age of 6–25 weeks (median 91 days, *n* = 24) all *Nsd1*^*−/−*^ mice developed signs of distress, significant hepatosplenomegaly with extensive cellular multi-organ infiltrations, reduced red blood cell (RBC) counts and hemoglobin levels, reticulocytosis, and severe thrombocytopenia (Fig. 2a–h, Supplementary Fig. 2h, Supplementary Table 1). White blood cell (WBC) counts were mostly within the normal range but “unclassified leukocytes” were detected and erythroblast-like cells were seen on peripheral blood smears (Fig. 2i, Supplementary Fig. 2i–k). Transplantation of BM cells from symptomatic *Nsd1*^*−/−*^ mice (alone or 1:1 in competition with normal cells) rapidly induced the same disease in lethally irradiated wild-type recipients, after a latency of 33 and 42 days, respectively, characterized by hepatosplenomegaly, multi-organ infiltration, anemia, thrombocytopenia, and erythroblasts in the periphery (Fig. 2j, Supplementary Fig. 21, Supplementary Table 2).

**Fig. 3:**
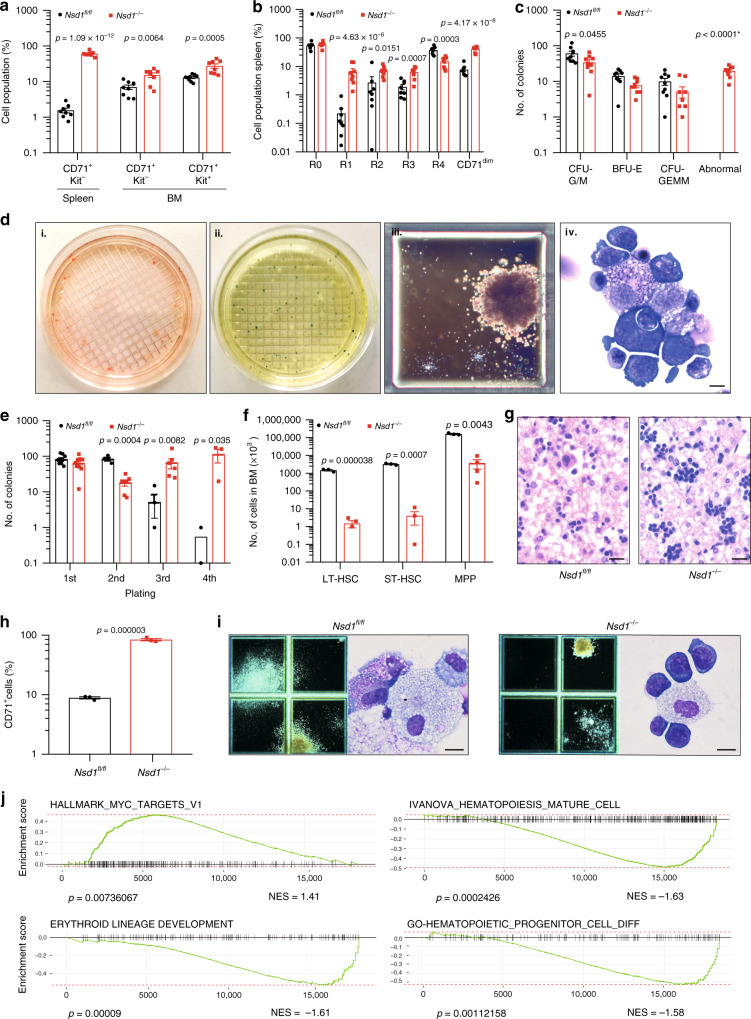
Cellular and molecular characterization of the erythroleukemia-like disease of *Nsd1*^*−/−*^ mice. **a** CD71^+^ and Kit^+^ cell populations (given in %) in single-cell suspensions of spleen and BM of healthy *Nsd1*^*fl/fl*^ (spleen, *n* = 8, BM, *n* = 9, black bars) and diseased *Nsd1*^*−/−*^ mice (spleen, *n* = 8; BM, *n* = 8, red bars). **b** Comparative flow cytometric analysis of erythroid maturation (R1–R4) of single-cell suspensions of total BM of healthy *Nsd1*^*fl/fl*^ (*n* = 9, black bars) and diseased *Nsd1*^*−/−*^ mice (*n* = 9, red bars). **c** Colony types formed by 4 × 10^4^ BM cells of *Nsd1*^*fl/fl*^ (*n* = 9, black bars) and *Nsd1*^*−/−*^ mice (*n* = 9, red bars) in growth-factor-containing MC (M3434). * indicating a *p* value smaller than 1 × 10^−15^. **d** Representative images (illustrating one out of four experiments) of MC (M3434) cultures of *Nsd1*^*−/−*^ BM cells demonstrating (i) abnormal large red colonies, (ii) partially benzidine-staining-positive colonies, (iii) a large dense isolated colony, and (iv) Wright Giemsa-stained cytospin of an isolated colony (×4 and ×1000, size bar = 10 μM). **e** Number of colonies in four consecutive rounds of plating in MC (M3434) formed by 4 × 10^4^ BM cells of *Nsd1*^*fl/fl*^ (black dots; 1^st^ plating: *n* = 9, 2^nd^ plating: *n* = 4, 3^rd^ plating: *n* = 3, 4^th^ plating: *n* = 2) and *Nsd1*^*−/−*^ mice (red squares; 1^st^ plating: *n* = 9, 2^nd^ plating: *n* = 7, 3^rd^ plating: *n* = 6, 4^th^ plating: *n* = 3). **f** Number of LT-HSC, ST-HSC, and MPP (×10^4^) in lineage-marker-depleted single-cell BM suspensions of *Nsd1*^*fl/fl*^ (*n* = 3, black bars) and *Nsd1*^*−/−*^ mice (*n* = 4, red bars) relative to the total number of lineage-depleted cells obtained during each procedure. **g** Representative HE-stained biopsies of E19.5 fetal livers from a *Nsd1*^*fl/fl*^ (left panel, illustrating one out of two experiments) and *Nsd1*^*−/−*^ (right panel, illustrating one out of four experiments) mouse (×400, size bar = 10 μM). **h** CD71^+^ cells (%) in E19.5 fetal livers of *Nsd1*^*fl/fl*^ (*n* = 3, black bar) and *Nsd1*^*−/−*^ (*n* = 3, red bar) mice. **i** Representative images of colonies in MC cultures (M3434) and Wright Giemsa-stained cytospin preparations from 4 × 10^4^ E17.5 fetal liver-derived hematopoietic cells of *Nsd1*^*fl/fl*^ (left panels, illustrating one out of three experiments) and Nsd*1*^*−/−*^ (right panels, illustrating one out of three experiments) mice (×2 and ×1000, size bars = 10 μM). **j** Gene set enrichment analysis (GSEA) (weighted Kolmogorov–Smirnov-like statistics, two-sided, with adjustment for multiple comparisons) of selected signatures of differentially expressed gene between *Nsd1*^*−/−*^ mice (*n* = 5) and littermate controls (*n* = 3). Values are presented as individual points, bar graphs represent the mean value of biological replicates, error bars as standard error of the mean. Statistical significances in **a**–**c**, **e**, **f**, **h** was tested with unpaired two-tailed *t*-test.

BM and spleen cells from diseased mice expressed modest levels of the transferrin receptor (CD71) and variable amounts of Kit and FcγRII/III, but were negative for CD34, B220, and Sca-1 (Fig. 3a, Supplementary Fig. 3a, b). Erythroid differentiation was defined by staining of CD71 and Ter119 progressing from immature CD71^low^Ter119^low^ (“R0”) to CD71^low^Ter119^high^ (“R4”) cells (Supplementary Fig. 3c)^23^. Whereas a decrease of the R4 fraction that was mostly evident in the spleens, all diseased *Nsd1*^*−/−*^ mice significantly accumulated CD71^dim^/Ter119^low^ cells in BM and spleen (Fig. 3b, Supplementary Fig. 3d). BM cells of diseased *Nsd1*^*−/−*^ mice formed reduced numbers of colonies in MC with significant reduction of CFU-GM and BFU-E colonies accompanied with sometimes large and abnormally dense, reddish and benzidine-staining positive “BFU-E-like” serially platable colonies, composed of myeloid and erythroid progenitors (Fig. 3c–e).

Diseased *Nsd1*^*−/−*^ BM contained reduced numbers of lineage marker negative, Kit^+^/Sca-1^+^ (LSK), long-term- (LT-HSC), and short-term repopulating hematopoietic stem cells (ST-HSC) (Fig. 3f). The number of multi-potent progenitors (MPP) and granulocytic-macrophage progenitors (GMP) was also reduced, common myeloid progenitors (CMP) were less affected, and the number of other progenitors (pre-GM, pre-MegE, MkP) did not significantly differ from littermate controls (Supplementary Fig. 3e, f).

As *Vav1*-promoter driven Cre expression resulted in significant reduction of *Nsd1* expression as early as at E13.5 of development, we also analyzed the impact of *Nsd1* inactivation during fetal liver hematopoiesis (Supplementary Fig. 3g, h)^24^. Hereby, we observed clusters of large cells with a dark-blue cytoplasm on E19.5 fetal liver sections (Fig. 3g). MC cultures did not display any significant differences in total colony number; however, E19.5 *Nsd1*^*−/−*^ fetal liver cells formed dense colonies of mostly CD71^+^ cells (Fig. 3h, i) (Supplementary Fig. 3i) resembling those formed by diseased adult BM (Fig. 3d).

Comparison of the transcriptomes of BM cells from symptomatic *Nsd1*^*−/−*^ mice (*n* = 5) with littermate controls (*n* = 3) (Supplementary Fig. 3j, Supplementary Data 1) revealed significant upregulation of 1705 (of 18301 genes, 9.3%) and downregulation of 1558 (8.5%) genes (FDR < 0.05). Gene set enrichment analysis (GSEA) revealed positive correlations between differentially expressed genes (DEGs) with a previously characterized signature of MYC targets and negative correlation with a gene signature of murine terminal erythroid differentiation (Fig. 3j)^25^. Collectively, these data show that inactivation of *Nsd1* in the hematopoietic system induces an erythroleukemia-like disease in mice^26^.

### Aberrant regulation of GATA1 in *Nsd1*^*−/−*^ erythroblasts

**Fig. 4:**
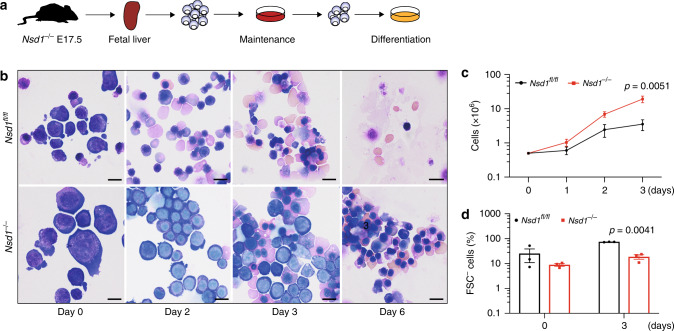
Impaired erythroid maturation of *Nsd1*^*−/−*^ erythroblasts. **a** Experimental setup: lineage-depleted E17.5 fetal liver cells of *Nsd1*^*fl/fl*^ and *Nsd1*^*−/−*^ mice were grown in maintenance medium (>6 days) before induction of maturation in differentiation cultures. **b** Representative images (illustrating one out of two maturation experiments) of Wright Giemsa-stained cytospin preparations of E17.5 *Nsd1*^*fl/fl*^ and *Nsd1*^*−/−*^ fetal liver-derived erythroblasts expanded in maintenance medium (day 0) and induced to maturate in differentiation medium, shown at days 2, 3, and 6 (×1000, the size bar = 10 μM). **c** Growth of fetal liver-derived *Nsd1*^*fl/fl*^ (*n* = 6, black line) and *Nsd1*^*−/−*^ (*n* = 4, red line) erythroblasts in differentiation medium. Living cells were counted using trypan blue exclusion. **d** Forward scatter-negative (FSC^−^) *Nsd1*^*fl/fl*^ (black bars) and *Nsd1*^*−/−*^ (red bars) living cells (%) before (day 0) and after 3 days in differentiation medium (*n* = 3). Values are presented as individual points, bar graphs represent the mean value of biological replicates, error bars as standard error of the mean. Statistical significances in **c**, **d** was tested with unpaired two-tailed *t*-test.

To elucidate the role of *Nsd1* in erythroleukemia, we first established culture conditions for primary erythroblasts that maintain cytokine dependency as well as differentiation potential towards enucleated erythrocytes (Fig. 4a)^27^. Growth of fetal liver (FL)-derived *Nsd1*^*−/−*^ erythroblasts did not significantly differ from littermate controls in maintenance medium (“MM”, containing dexamethasone, hIGF1, cholesterol, and hEPO). In contrast, differentiation of *Nsd1*^*−/−*^ cells was significantly impaired while control cells completely matured in mSCF and hEPO containing differentiation-inducing medium (“DM”) (Fig. 4b–d, Supplementary Fig. 4a–d).

**Fig. 5:**
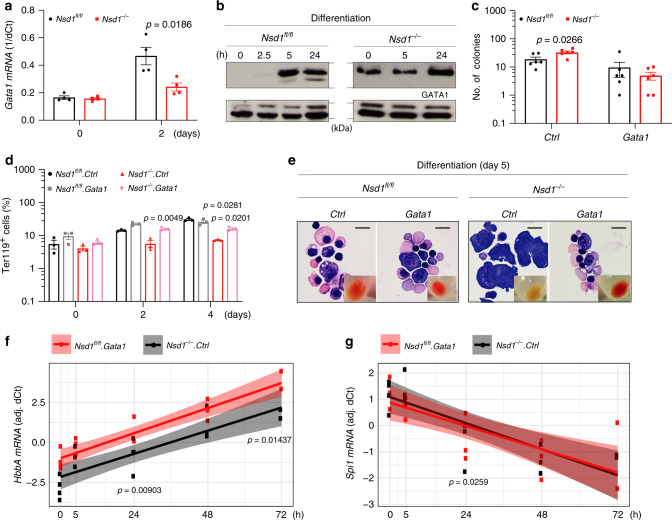
Aberrant regulation of GATA1 expression in *Nsd1*^*−/−*^ erythroblasts. **a** Relative *Gata1* mRNA expression levels (1/dCt) in BM-derived erythroblasts from *Nsd1*^*fl/fl*^ (*n* = 4, black bars) and *Nsd1*^*−/−*^ mice (*n* = 4, red bars) in maintenance medium (day 0) and after 2 days in differentiation medium. Ct values were normalized to *Gapdh* expression. **b** GATA1 protein levels in *Nsd1*^*fl/fl*^ (left panels) and *Nsd1*^*−/−*^ (right panels) BM-derived erythroblasts expanded in maintenance medium (0 h) and in differentiation medium (2.5, 5, and 24 h). LAMIN-A/C was used as immunoblot loading control for nuclear proteins (one out of two experiments). **c** Number of colonies formed by 5 × 10^3^ lineage-marker-depleted BM-derived erythroblasts in MC (M3434) from *Nsd1*^*fl/fl*^ (black bars) and *Nsd1*^*−/−*^ mice (red bars) transduced with *pMSCV-puro* (*Ctrl*) or *pMSCV-mGata1-puro (Gata1)* (*n* = 6 per group). **d** Ter119 expression (Ter119^+^, in %) in maintenance medium (0 h) and after 2 and 4 days in differentiation medium of *Nsd1*^*fl/fl*^ (black and gray bars) and *Nsd1*^*−/−*^ (red and pink bars) BM-derived erythroblasts transduced with control virus (*Ctrl*, black and red bars) or *Gata1-*expressing virus (*Gata1*, gray or pink bars) (*n* = 3 per group). **e** Representative images (one out of two experiments) of Wright Giemsa-stained cytospin preparations and cell pellets (small insets) of *Nsd1*^*fl/fl*^ and *Nsd1*^*−/−*^ BM-derived erythroblasts transduced with control virus (*Ctrl*) or *Gata1-*expressing virus (*Gata1*) after 5 days in differentiation medium (×600, size bars = 10 μm). **f** *HbbA* and **g** *Spi1* mRNA levels in BM-derived *Nsd1*^*−/−*^ BM-derived erythroblasts transduced with control virus (*Ctrl*, black dots) or *Gata1*-expressing virus (*Gata1*, red dots) measured 0, 5, 24, 48, and 72 h in differentiation medium. Values are residual ΔCT relative to *Gapdh*, after adjustment for effect of individual mouse (Tukey test for difference in expression between transductions at 24 h in linear model with interaction between time a and transduction, adjusting for effect of mouse, two-sided, adjusted for multiple comparisons). Values are presented as individual points, bar graphs represent the mean value of biological replicates, error bars as standard error of the mean. Statistical significances in **a**, **c** was tested with unpaired two-tailed *t*-test. Statistical significance in **d** was tested with either unpaired (*Nsd1*^*fl/fl*^*.Gata1* vs. *Nsd1*^*−/−*^*.Gata1*) or paired *(Nsd1*^*−/−*^*.Ctrl* vs*. Nsd1*^*−/−*^*.Gata1*) two-tailed *t*-test. Statistical significance in **f**, **g** was tested using Tukey test for difference in expression between transductions at 24 h in linear model with interaction between time a and transduction, adjusting for effect of mouse.

Erythropoiesis is controlled by the transcriptional master regulator GATA1^28^. While *Nsd1*^*−/−*^ erythroblasts expressed reduced *Gata1* mRNA levels in DM, GATA1 protein expression remained abundant in maintenance medium and during induced differentiation (Fig. 5a, b, Supplementary Fig. 4e, f). Interestingly, retroviral expression of a full-length murine *Gata1* cDNA resulting in 2.6–3.2-fold increased level of exogenous protein not only reduced the number of aberrant colonies in MC but also induced terminal maturation of *Nsd1*^*−/−*^ erythroblasts (Fig. 5c–e, Supplementary Fig. 4g).

To study the impact of NSD1 on GATA1 transcription factor activity we compared the expression of previously proposed GATA1 target genes. Overexpression of GATA1 promoted induced expression of several genes in *Nsd1*^*−/−*^ cells, including *Hba-A, Hbb-B*, and *Bcl2l1*, which are normally activated by GATA1 during differentiation (Fig. 5f, Supplementary Fig. 4h, i). In contrast, exogenous *Gata1* did not affect expression of *Spi1* but further reduced expression of *Kit* and *Gata2* known to be downregulated by GATA1 during normal erythroid differentiation (Fig. 5g, Supplementary Fig. 4j, k). Together, these data suggest that the activation of GATA1-controlled target genes during erythroid differentiation is modulated by NSD1^29^.

### NSD1-SET is essential for in vitro erythroblast maturation

**Fig. 6:**
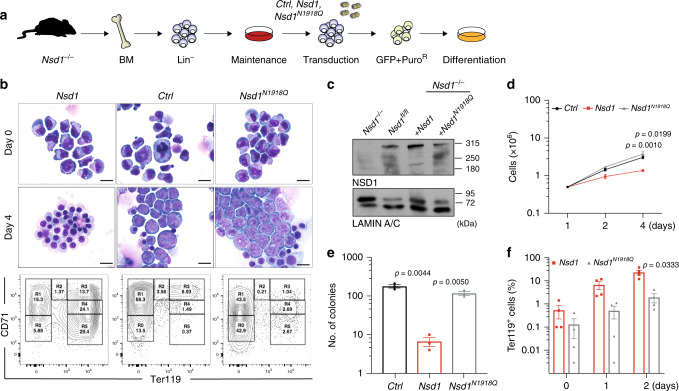
NSD1-SET is essential for in vitro erythroblast maturation. **a** Experimental setup: BM-derived *Nsd1*^*fl/fl*^ and *Nsd1*^*−/−*^ BM-derived erythroblasts were transduced with either *pMSCV-GFP-Puro (Ctrl), pMSCV-Nsd1-GFP-Puro (Nsd1)*, or *pMSCV-Nsd1*^*N1918Q*^*-GFP-Puro (Nsd1*^*N1918Q*^*)* in maintenance medium, GFP^+^ cells were expanded in the presence of Puromycin before induced differentiation and analysis. **b** Representative pictures of Wright Giemsa-stained cytospin preparations transduced with *Nsd1, Nsd1*^*N1918Q*^, or control virus in maintenance medium (day 0, top panels) and after 4 days in differentiation medium (middle panels). The lower panels show flow cytometric analysis of CD71 and Ter119 expression of the cells after 4 days in differentiation medium. These data represent one of four independent experiments (×1000, size bars = 10 μM). **c** Western blot analysis showing NSD1 protein expression in 1 × 10^6^ *Nsd1*^*fl/fl*^ and *Nsd1*^*−/−*^ (untransduced), and *Nsd1*^*−/−*^ transduced erythroblasts either expressing *Nsd1* or *Nsd1*^*N1918Q*^ in maintenance medium. LAMIN-A/C was used as a loading control. These data represent one out of two experiments. **d** Growth of *Nsd1* (red line), *Nsd1*^*N1918Q*^ (gray line), or the control *(Ctrl*, black line) virus transduced *Nsd1*^*−/−*^ BM-derived erythroblasts in differentiation medium (1–4 days). Nucleated living cells were counted by the Trypan blue exclusion (*n* = 3 per group). **e** Number of colonies formed by 1 × 10^4^ *Nsd1* (red bar), *Nsd1*^*N1918Q*^ (gray bar) or the control virus (*Ctrl*, black bar) transduced *Nsd1*^*−/−*^ BM-derived erythroblast in MC (M3434) after 11 days (*n* = 3 per group). **f** Ter119^+^ stained *Nsd1*^*−/−*^ BM-derived erythroblasts transduced with *Nsd1* (red bars) and *Nsd1*^*N1918Q*^ (gray bars) in maintenance medium (day 0) and after 1 and 2 days in differentiation medium (*n* = 4). Values are presented as individual points, bar graphs represent the mean value of biological replicates, error bars as standard error of the mean. Statistical significances in **d**–**f** tested with a paired two-tailed *t*-test.

To address how NSD1 controls erythroid differentiation we compared the effects of expression of wild type (*Nsd1*) or a catalytically inactive SET domain mutant (*Nsd1*^*N1918Q*^) in *Nsd1*^*−/−*^ erythroblasts (Fig. 6a)^30^. Expression of *Nsd1* but not *Nsd1*^*N1918Q*^ significantly rescued terminal maturation as illustrated by cellular morphology, a shift of CD71 and Ter119 surface expression, formation of reddish cell pellets, reduced proliferation, and reduced colony formation in MC (Fig. 6b–f, Supplementary Fig. 5a–c).

**Fig. 7:**
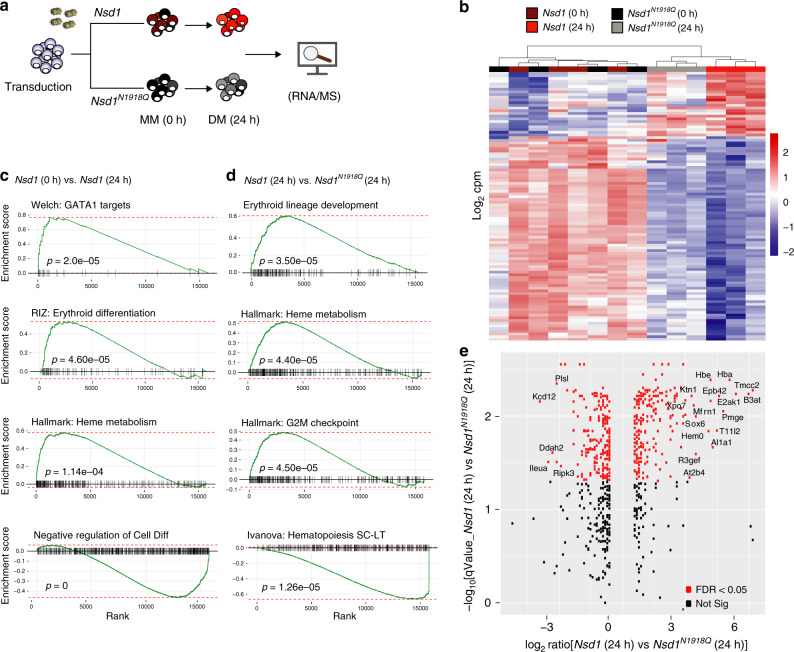
*Nsd1* expression induces an erythroid gene and protein signature. **a** Experimental setup: *Nsd1*^*−/−*^ BM-derived erythroblasts expressing either *Nsd1* or *Nsd1*^*N1918Q*^ were analyzed during expansion in maintenance medium (0 h) and after 24 h in differentiation medium by RNA-seq and global proteome analysis. **b** Heatmap of the top 100 differentially expressed genes (corresponding to FDR < 1.06 × 10^9^) of *Nsd1*^*−/−*^ BM-derived erythroblasts expressing *Nsd1* (brown squares) and *Nsd1*^*N1918Q*^ (black squares) in maintenance medium, and after 24 h in differentiation medium (*Nsd1*, red squares; *Nsd1*^*N1918Q*^, gray square). Columns clustering was done by Wards linkage on correlations. **c** Gene set enrichment analysis (GSEA) (weighted Kolmogorov–Smirnov-like statistics, two-sided, with adjustment for multiple comparisons) of differential expression between *Nsd1*^*−/−*^ BM-derived erythroblast expressing *Nsd1* before and after 24 h in differentiation medium. **d** GSEA (weighted Kolmogorov–Smirnov-like statistics, two-sided, with adjustment for multiple comparisons) of differential expression between *Nsd1*^*−/−*^ BM-derived erythroblasts expressing either *Nsd1* or *Nsd1*^*N1918Q*^ kept for 24 h in differentiation medium. **e** Differential protein expression of *Nsd1*^*−/−*^ erythroblasts expressing *Nsd1* or *Nsd1*^*N1918Q*^ kept for 24 h in differentiation medium (*n* = 3 per group, FDR < 0.05, *p* value <0.05). Labels are shown for proteins with both logFC > 3 and FDR < 0.05.

To address the molecular mechanisms, we measured DEGs and total proteome expression in BM-derived *Nsd1*^*−/−*^ erythroblasts retrovirally expressing *Nsd1* or *Nsd1*^*N1918Q*^ expanded in MM and kept for 24h in DM (Fig. 7a). After 24 h in DM, expression of about 2% of the genes significantly (*p* < 0.05) increased (270 of 15804) or decreased (318 of 15804) in cells expressing *Nsd1* compared to *Nsd1*^*N1918Q*^ (Fig. 7b, Supplementary Data 2). Among more highly expressed genes we found the cell cycle regulator *Cdk2* and the epigenetic regulator *Kmt5a (Setd8)* previously shown to be important for erythroid differentiation^31–34^. Among the lower expressed genes, we found the transcription factor *Gata2* and the RNA-binding protein *Zfp36l2* known for their role in regulating self-renewal of hematopoietic stem and erythroid progenitor cells^35,36^. GSEA revealed significant (*p* < 0.0001) positive correlations between DEGs of *Nsd1*^*−/−*^ cells expressing *Nsd1* for 24 h in DM with signatures linked to heme metabolism, erythroid differentiation, and putative GATA1 target genes, and inverse correlation with a negative regulatory differentiation signature (Fig. 7c, Supplementary Data 3). DEG between cells expressing *Nsd1* or the *Nsd1*^*N1918Q*^ mutant kept 24 h in DM did not only positively correlate with the expression signature of murine terminal erythroid differentiation but also with signatures related to heme metabolism and cell cycle checkpoints, and negatively with signatures related to hematopoietic stemness (Fig. 7d, Supplementary Data 4). In parallel to differential mRNA expression we also determined changes of the global proteome. *Nsd1*^*−/−*^ erythroblasts expressing wild-type *Nsd1* for 24 h in DM expressed significant higher protein levels of several proposed GATA1 targets like hemoglobin (HBA, HBB1, HBE), exportin 7 (XPO7), or mitoferrin (MFRN1) (Fig. 7e, Supplementary Fig. 5d, Supplementary Data 5, and Supplementary Table 3)^37,38^. Collectively, these observations suggest that the catalytic activity of NSD1 is essential for terminal erythroid maturation and regulation of GATA1 targets.

### *Nsd1* regulates GATA1 chromatin binding and protein interactions

**Fig. 8:**
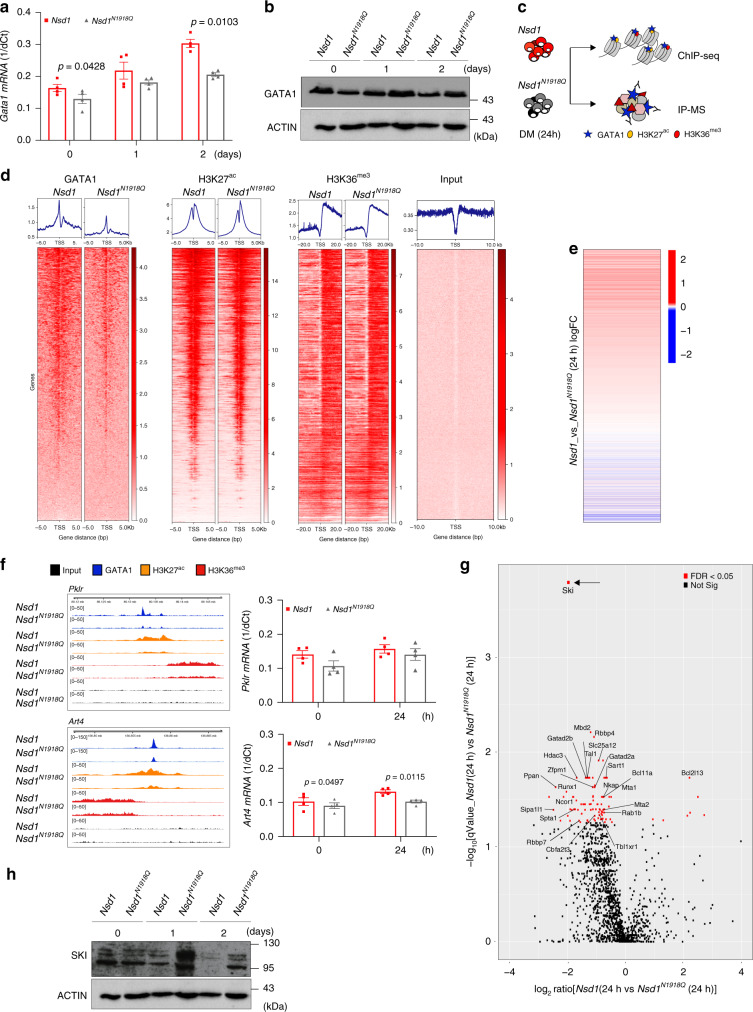
*Nsd1* expression increases GATA1 chromatin binding and changes GATA1 protein interaction partners during induced differentiation of *Nsd1*^*−/−*^ cells. **a** Relative *Gata1* mRNA expression levels (1/dCt) in *Nsd1*^*−/−*^ BM-derived erythroblasts virally expressing *Nsd1* (red bars) or *Nsd1*^*N1918Q*^ (gray bars) expanded in maintenance medium (day 0) or after 1 and 2 days in differentiation medium. Values were normalized to *Gapdh* (*n* = 4 per group). **b** Western blot showing GATA1 protein expression in 1 × 10^6^ *Nsd1*^*−/−*^ BM-derived erythroblasts expressing *Nsd1* or *Nsd1*^*N1918Q*^ upon expansion in maintenance medium (day 0), and after 1 and 2 days in differentiation medium. Actin was used as a loading control. This data represents one of two experiments. **c** Experimental setup of the ChIP-seq and IP-MS experiment. **d** Heatmaps of genome-wide ChIP-seq signals in *Nsd1*^*−/−*^ BM-derived erythroblasts expressing *Nsd1* (left column) or *Nsd1*^*N1918Q*^ (right column) after 24 h in differentiation medium for GATA1, H3K27^ac^, and H3K36^me3^. All heatmaps are sorted decreasingly according to read coverage around transcriptional start sites (TSS) of GATA1 (leftmost). Input denotes sheared non-immunoprecipitated DNA (rightmost), serving as visual control. Density plots above each heatmap depicts corresponding averaged binding around TSS. **e** One-dimensional heatmap of logFC between gene expression of *Nsd1*^*−/−*^ BM-derived erythroblasts expressing *Nsd1* or *Nsd1*^*N1918Q*^ after 24 in differentiation medium (as presented in Fig. 5h, j) sorted according to read coverage around TSS for H3K27^ac^ ChIP (data as shown in panel **c**, sorted independently. Only overlapping genes are displayed). **f** Integrated genome viewer (IGV) representation of GATA1, H3K27^ac^, and H3K36^me3^ ChIP peaks in the *Pklr* (top panel) and *Art4* gene locus (lower panel) from *Nsd1*^*−/−*^ BM-derived erythroblasts either expressing *Nsd1* or *Nsd1*^*N1918Q*^ after 24h in differentiation medium. Right panels show *Pklr* and *Art4* mRNA relative expression levels (1/dCt) in *Nsd1*^*−/−*^ BM-derived erythroblasts expressing *Nsd1* or *Nsd1*^*N1918Q*^ in maintenance medium (day 0) and after 24 h differentiation medium. Values are shown as relative expression normalized to *Gapdh* (*n* = 4). **g** Volcano plot of differential protein enrichments by GATA1 immunoprecipitation (GATA1-IP) in *Nsd1*^*−/−*^ BM-derived erythroblasts either expressing *Nsd1* or *Nsd1*^*N1918Q*^ kept for 24h in differentiation medium, each group is normalized to IgG control (*n* = 2). Significantly reduced GATA1-SKI association (indicated by a black arrow) was observed upon expression of *Nsd1* compared to *Nsd1*^*N1918*Q^ (FDR < 0.05). **h** Western blot analysis showing SKI protein expression in 1 × 10^6^ BM-derived *Nsd1*^*−/−*^ erythroblasts either expressing *Nsd1* or *Nsd1*^*N1918Q*^ during expansion in maintenance medium (day 0), and after 1 and 2 days in differentiation medium. Actin was used as a loading control. This data represent one of two experiments. Values are presented as individual points, bar graphs represent the mean value of biological replicates, error bars as standard error of the mean. Statistical significances in **a**, **f** tested with a paired two-tailed *t*-test.

As expression of wild type or mutant *Nsd1* did not overtly change GATA1 protein levels in *Nsd1*^*−/−*^ erythroblasts kept for 2 days in DM, we compared chromatin binding and putative protein interactions of GATA1 by ChIP and IP-MS after 24 h of induced differentiation (Fig. 8a–c). Hereby, we found increased occupancy of GATA1 at over 3000 sites in the genome overlapping with 1362 genes (*p* < 0.01) in cells expressing *Nsd1* in comparison to the catalytically inactive *Nsd1*^*N1918Q*^ mutant (Fig. 8d). Of genes with significantly increased binding of GATA1, 731 of them had the promotor regions decorated by H3K27^ac^ while H3K36^me3^ marks overlapped with 1179 gene bodies (Supplementary Data 6). Hence, while global levels of GATA1 remains constant, reintroduction of *Nsd1* resulted in increased DNA binding to available GATA1 sites in promotor regions, similarly reflected in changes in H3K36^me3^ and H3K27^ac^ at the genomic coordinates. Interestingly, changes in gene expression aligned with H3K27^ac^ around TSS, confirming that these epigenetic marks are directly regulating the down-stream transcriptional programming (Fig. 8e). However, we could not detect any gene loci with statistically significant increase of all three, GATA1, H3K36^me3^, and H3K27^ac^ (Supplementary Data 7 and 8), which could be a matter of temporal distance along the activation pathway.

Nevertheless, *Nsd1*-induced regulation of several erythroid regulators was associated with simultaneously changed GATA1 binding, H3K27^ac^ and H3K36^me3^ marks. The *Pklr* gene locus, encoding for the liver-red cell pyruvate kinase linked to erythroid differentiation and *Art4*, encoding for the developmentally regulated Dombrock blood group glycoprotein, were both higher expressed in *Nsd1*^*−/−*^ cells expressing wild-type *Nsd1* associated with a narrow GATA1 peak in the promotor region within a broader decoration of H3K27^ac^, followed by gene body-wide H3K36^me3^ marks (Fig. 8f)^39,40^. The opposite was observed for the gene encoding for *Fgf*2 (fibroblast growth factor 2) associated with inhibition of efficient erythroid differentiation that appeared higher expressed in *Nsd1*^*N1918Q*^ than in *Nsd1*-expressing cells (Supplementary Fig. 6a)^41^. Immunoblot and masspectrometry analysis revealed globally reduced mono-, di-, and tri-methylated H3K36 in *Nsd1*^*−/−*^ erythroblasts expressing the inactive *Nsd1*^*N1918Q*^ mutant compared to those expressing wild-type *Nsd1* (Supplementary Fig. 6b, c).

To address whether impaired chromatin binding and transactivation of GATA1 in the absence of *Nsd1* might be associated with altered protein interactions we immunoprecipitated GATA1 followed by mass spectrometry in *Nsd1*^*−/−*^ cells either expressing wild-type *Nsd1* of the inactive *Nsd1*^*N1918Q*^ mutant kept for 24 h in DM. We identified 413 differentially expressed proteins (*p* < 0.05) (Supplementary Data 9), of which the most significant ones included known interactors of GATA1 such as MBD2, RBBP4, ZFPM1, RUNX1, and TAL1 suggesting functionality of the assay (Fig. 8g)^42^. Interestingly, mass spectrometry analysis revealed that differentiation of *Nsd1*-expressing *Nsd1*^*−/−*^ erythroblasts was associated with a highly significant reduction (logFC = −1.96; *p* < 1.08 × 10^−7^) of the transcriptional repressor protein SKI previously proposed to interact with and inhibit GATA1 activation, most likely in cooperation with the nuclear co-repressor (NCoR) complex (Fig. 8g, h, Supplementary Fig. 6d)^43,44^. Notably, several members of the NCoR complex (NCOR1, HDAC3, TBLXR1) co-appeared with SKI, as differentially regulated (Fig. 8g, Supplementary Data 9).

### SKI knockdown differentiates *Nsd1*^*−/−*^ erythroblasts

**Fig. 9:**
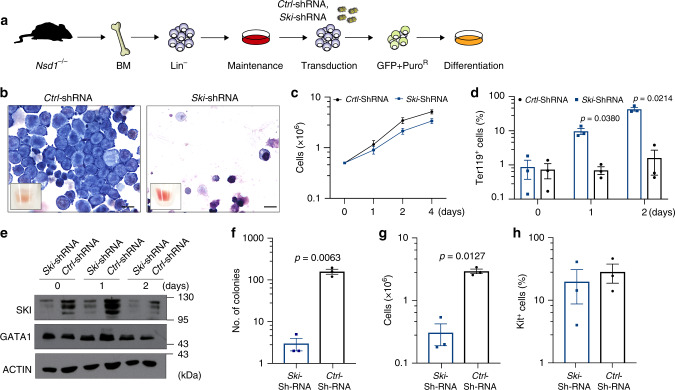
SKI knockdown results in terminal differentiation of *Nsd1*^*−/−*^ erythroblasts. **a** Experimental setup: BM-derived *Nsd1*^*−/−*^ erythroblasts were transduced with either *pLMP-empty-shRNA-GFP-Puro* (*Ctrl-*shRNA) or *pLMP-Ski-shRNA-GFP-Puro* (*Ski-*shRNA) in maintenance medium, sorted for GFP, and selected with Puromycin for 2 days before induced differentiation and analysis. **b** Representative images of Wright Giemsa-stained cytospin preparations of control (*Ctrl-*shRNA, left panel) and Ski shRNA (*Ski-*shRNA*)*, right panel) transduced *Nsd1*^*−/−*^ BM-derived erythroblasts after 2 days in differentiation medium. The small insets show the cell pellets before analysis. These data illustrate one of three experiments (×1000, size bar = 10 μM). **c** Growth of *Nsd1*^*−/−*^ BM-derived erythroblasts transduced with Ski- *(Ski-*shRNA, blue line) or control shRNA (*Ctrl-*shRNA, black line) grown for 4 days in differentiation medium. Nucleated living cells were counted by Trypan blue exclusion (*n* = 3 per group). *P* value > 0.05 for all time points. **d** Fraction of Ter119^+^ cells (%) of *Nsd1*^*−/−*^ BM-derived erythroblasts transduced with Ski- *(Ski-*shRNA, blue bars) or control (*Ctrl-*shRNA, black bars) virus grown for 2 days in differentiation medium (*n* = 3 per group). **e** Western blot showing SKI and GATA1 protein expression in *Nsd1*^*−/−*^ BM-derived erythroblasts transduced with Ski (*Ski-*shRNA) or control (*Ctrl-*shRNA) virus during expansion in maintenance medium (day 0) and following 1–2 days in differentiation medium. Actin was used as a loading control. These data represent one of three experiments. **f** Total number of colonies counted at day 11 after plating of 1 × 10^4^ *Nsd1*^*−/−*^ BM-derived erythroblasts expressing either *Ski shRNA* (*Ski-shRNA*, blue bar) or control (*Ctrl-*shRNA, black bar) in MC (M3434) (*n* = 3 per group). **g** Total number of cells obtained from 1 × 10^4^ *Nsd1*^*−*/−^ BM-derived erythroblasts expressing either *Ski shRNA* (*Ski-shRNA*, blue bar) or control (*Ctrl-*shRNA, black bar*)* after 11 days in MC (M3434) (*n* = 3 per group). **h** Percentage of Kit^+^ living cells obtained from 1 × 10^4^ *Nsd1*^*−/−*^ BM-derived erythroblasts expressing either *Ski shRNA (Ski-shRNA*, blue bar) or control (*Ctrl-*shRNA, black bar) after 11 days in MC (M3434) (*n* = 3 per group). *P* value > 0.05 for all time points. Values are presented as individual points, bar graphs represent the mean value of biological replicates, error bars as standard error of the mean. Statistical significances in **c**, **d**, **f**, **g**, **h** tested with a paired two-tailed *t*-test.

To functionally explore reduced GATA1-SKI association upon *Nsd1* expression, we asked whether experimental shRNA-mediated reduction of SKI might be sufficient to initiate maturation of *Nsd1*^*−/−*^ erythroblasts (Fig. 9a). We found that SKI knockdown significantly increased in vitro induced terminal maturation of erythroblasts from three independent *Nsd1*^*−/−*^ mice, as shown by cellular morphology, flow cytometry (CD71/Ter119/Kit), and proliferation (Fig. 9b–d, Supplementary Fig. 7a). SKI knockdown did not alter GATA1 protein levels (Fig. 9e). Notably, prolonged culture in DM was associated with a general reduction of SKI levels suggesting a role for SKI during initiation rather than terminal differentiation. SKI knockdown also significantly reduced clonogenic growth, total number of cells, and Kit^+^ expression of the cells in MC (Fig. 9f–h, Supplementary Fig. 7b). Collectively, these data suggest that in the absence of *Nsd1*, terminal erythroid maturation is blocked as a consequence of impaired GATA1 transactivation dependent on its association with the transcriptional repressor SKI.

## Discussion

The observations that reduced expression of NSD1 altered erythroid clonogenic growth of human CD34^+^ cells and significantly impaired terminal erythroid maturation leading to an erythroleukemia-like disease in mice characterizes NSD1 as a regulator of erythroid differentiation. Mechanistically, we found that NSD1 activity regulated chromatin binding and target gene activation by the erythroid master regulator GATA1.

### *Nsd1* inactivation phenocopies hallmarks of *Gata1* deficiencies in mice

Earlier studies showed that constitutive *Gata1* gene inactivation in mice resulted in embryonic death at approximately E11.5 due to arrested maturation of primitive erythroid cells^4^. In contrast, some mice with 95% reduced *Gata1* mRNA expression due to a knockdown mutation (*Gata1*^*+/1.05*^) developed a late-onset B-cell lymphoproliferative disease or an earlier erythroleukemia-like disease^5^. Similar to *Nsd1*^*−/−*^ erythroblasts, *Gata1*^*1.05/+*^ erythroleukemia cells were able to differentiate into mature erythrocytes when complemented with full-length *Gata1* (ref. ^45^). In contrast to *Gata1*^*1.05/+*^ mice, *Nsd1*^*−/−*^ mice developed a fully penetrant erythroleukemia-like phenotype after a shorter latency (Fig. 2).

The best-studied in vivo erythroleukemia model is *Friend’s virus complex* induced erythroblastosis in which viral integration results in aberrant expression of the *Spi1* gene encoding for PU.1 (ref. ^46^). Similar to *Nsd1*^*−/−*^, *Spi1* transgenic mice develop anemia, thrombocytopenia, and multi-organ infiltration of erythroblasts progressing from an EPO-dependent stage to EPO-independence by acquisition of activating mutations in the *c-kit* receptor tyrosine kinase^47,48^. However, very similar to *Nsd1*^*−/−*^ erythroblasts, *Friend’s virus* erythroblastosis-derived MEL cells constitutively expressed GATA1 protein that could not be explained by the interaction with PU.1 (Fig. 5)^49^. In addition, conditional activation of exogenous *Gata1* was also reported to induce erythroid differentiation in some MEL cell lines^49,50^.

### *Nsd1* controls GATA1 protein interaction and activation of erythroid regulators

To study the mechanism of *Nsd1*-controlled erythroid differentiation we faced the problem that primary erythroblast cultures can contain significant fractions of myeloid cells, which are not present in *Nsd1*^*−/−*^ cultures. Therefore, we chose to virally express WT or a previously reported catalytically inactive *Nsd1*^*N1918Q*^ SET-mutant in *Nsd1*^*−/−*^ erythroblasts^30^. However, not only the large size of the *Nsd1* ORF drastically impaired the gene transfer efficacy, transduced cells also did not tolerate high levels of exogenous *Nsd1*, which limited generation of stably expressing cells in time and numbers. Nevertheless, low-level mRNA expression resulted in detectable *Nsd1* protein expression sufficient to restore terminal maturation of *Nsd1*^*−/−*^ erythroblasts in a methyltransferase activity-dependent manner (Fig. 6). Interestingly, *Nsd1* expression was associated with increased binding to and transactivation of a large number of previously proposed GATA1 target genes associated with changes in H3K27^ac^ and H3K36^me3^ marks (Fig. 8). These observations led us to speculate that in the absence of *Nsd1*, GATA1 might be functionally trapped in some saturated interactions that may limit its transactivation potential, which can be overcome by expression of additional “free” GATA1.

Characterization of putative GATA1 interactions by immunoprecipitation and mass spectrometry suggested that in the absence of *Nsd1*, GATA1 associates with potent transcriptional repressors (Fig. 8). Notably, expression of wild type but not the inactive SET *Nsd1* mutant resulted in a highly significant reduced association of GATA1 with the transcriptional co-repressor SKI. SKI is well known for its role as a regulator of the TGF-beta/Smad signaling pathway^51,52^. SKI was also found to be overexpressed in AML and proposed to repress retinoic acid receptor and RUNX1-mediated signaling.^53–55^. In addition, SKI was reported to control HSC fitness in myelodysplastic syndromes (MDS)^56^. Most importantly, SKI was shown to physically interact, to repress GATA1-mediated transactivation, and to block erythroid differentiation by blocking its interaction with DNA^43^ . In addition, SKI-mediated repression seemed to be NCoR dependent, and several NCoR complex proteins were altered in GATA1 pulldowns upon expression of *Nsd1* (Fig. 8)^44^. These observations suggest that the methyltransferase activity of NSD1 controls the interaction of GATA1 with SKI or other, yet to be defined mediators.

Very recent studies using quantitative proteomics revealed that co-repressors are dramatically more abundant than co-activators in erythroblasts^57^. How the lack of *Nsd1* directly regulates differential interaction of GATA1 co-activators and co-repressors remains to be elucidated. One can hypothesize that *Nsd1*-mediated H3K36 methylation provides the anchors for ultimate accumulation of sufficient co-activators on critical target gene loci.

Recent work suggested that H3K36 methylation is critical for normal erythroid differentiation. A conditional H3K36M mutation severely affected the murine hematopoietic system resulting in defects that partially phenocopy those observed in the *Nsd1*^*−/−*^ mice. *H3K36M* transgenic mice also developed anemia, thrombocytopenia, and splenomegaly. Most notably, these mice also showed a dramatic increase in early Ter119^−^ erythroid progenitor cells in the BM but also in the periphery^58^. Another study found that reduced H3K36^me2^ by *Nsd1* inactivation in ES cells resulted in re-localization of the DNMT3A DNA methyltransferase, which interacts with the H3K36^me3^ through its PWWP domain. This led to hypomethylation of euchromatic intergenic regions as observed in SOTOS patients having *NSD1* loss of function mutations^59^. Interestingly, normal erythroid maturation and particularly at transition from CFU-E to proerythroblasts was found to correlate with activation of a significant number of genes associated with gained DNA methylation on selective genes including numerous GATA1 targets^60^. Together, these findings suggest that the loss of H3K36 methylation and redistribution of DNMT3A could be directly responsible for impaired binding of GATA1 and its co-activators. The fact that we pulled down DNMT3A by immunoprecipitation of GATA1 in *Nsd1*^*−/−*^ cells (Supplementary Datas 10 and 11) suggests that GATA1 binding could indeed not only be dependent on H3K36me but also on DNMT3A-mediated DNA methylation.

### NSD1, SKI, and human erythroleukemia

*Nsd1* gene inactivation during late stage fetal liver hematopoiesis induced a fully penetrant lethal disease that phenocopied several aspects of acute erythroleukemia, a rare form of human AML^6^. Putative loss of function missense or frameshift *NSD1* mutations have been found in various human cancers including AML (https://cancer.sanger.ac.uk/cosmic/gene/analysis?ln=NSD1). Interrogation of the cancer cell line encyclopedia (CCLE) revealed that very few human cancer cell lines express even negligible levels of NSD1 mRNA and protein, including F-36P, a cell line established from a patient with acute erythroleukemia (https://portals.broadinstitute.org/ccle/page?gene=NSD1)^61^. Notably, very recent work revealed several cases of childhood acute erythroleukemia that harbored fusion genes involving *NSD1*^7^. Based on our findings one can speculate that in such cases the fusion may either act in a dominant-negative manner to *NSD1* expressed from the non-arranged allele, or the presence of LOH is reducing NSD1 activity as recently reported for a significant number of solid cancers^21^. Interestingly, we also found aberrantly high SKI expression levels in tumor cells of some erythroleukemia patients, and that in vivo overexpression of a *SKI* ORF in BM-derived HSPC resulted in an erythroleukemia-like disease in mice, suggesting that SKI expression may not only be critical for impaired erythroid differentiation in *Nsd1*^*−/−*^ mice but also a driver of the human disease^62^. Collectively, our observations suggest that impaired NSD1 activity functionally interferes with lineage-associated transcriptional master regulators such as GATA1 resulting in impaired cellular differentiation as a first step to malignant transformation.

## Methods

### Data presentation and statistical analysis

Bar graphs in the figures represent the mean value of biological replicates. Error bars are standard error of the mean (mean ± SEM). Statistical significance was tested with unpaired (*Nsd1*^*fl/fl*^ vs. *Nsd1*^*−/−*^*)* or paired (viral transduction in *Nsd1*^*−/−*^ cells) two-tailed *t*-test, assuming equal variance, unless otherwise specified. Statistical test was performed in log_10_ space, or for qPCR kept in log_2_ space.

### shRNA-mediated knockdown

Human CD34^+^ cells were obtained by enrichment using the CD34 MicroBead Kit (Miltenyi Biotec, Bergisch Gladbach, Germany) from peripheral blood or cord blood from healthy donors and kept in StemLine II medium (Sigma Aldrich, Buchs, Switzerland), supplemented with human cytokines such as 50 ng/ml hTPO (Peprotech, London, UK), 50 ng/ml hFLT3 ligand (Peprotech, London, UK), 50 ng/ml hSCF (Peprotech, London, UK), and 1 U/ml hEPO (Eprex 4000, Pharmacy of University Hospital Basel). shRNAs were expressed from lentiviral vectors (*pLKO.1*). For transduction lentiviral stock was produced by transient co- transfection of packaging vectors (*pMD2G, pMLDg/PRE, pRSV/Rev*) and respective lentiviral shRNA plasmid (shRNA Ctrl and shRNA *NSD1* #353, #369 and #372) using lipofectamine 2000 (Invitrogen, Thermo Fisher Scientific, Reinach, Switzerland) in HEK293T-LX cells kept in DMEM (Gibco, Lubio, Thermo Fisher Scientific, Reinach, Switzerland) with 10% FCS and 1% penicillin/streptomycin. Viral supernatants were harvested 48 and 72 h after transfection, snap frozen in liquid nitrogen, and stored in −80 °C until usage. Cells were spin-infected in the presence of 5 μg/ml polybrene (Sigma Aldrich, Buchs, Switzerland) with virus for 90 min, 2500 r.p.m. at 30 °C. Six hours after spin infection, cells were washed with PBS and plated in StemLine II cytokine enriched medium. Two days after spin infection cells were selected with 2 µg/ml puromycin (Gibco, Thermo Fisher Scientific, Reinach, Switzerland).

### Transgenic mice

Mice carrying a *Nsd1*^*+/L3*^ allele were previously described^22^. The floxed *pgk-neomycin* selection cassette was removed by viral *Cre* expression in ES cells, leaving two *loxP* sites flanking the largest coding exon 5, here referred as *Nsd1*^*fl/fl*^. *Nsd1*^*fl/fl*^ mice were intercrossed with a *Vav1-iCre*^*tg/+*^ transgenic strain leading to inactivation of the gene in fetal and adult hematopoiesis^63^. All mice in this study were kept under specific pathogen-free conditions. Mice were genotyped using the KAPA Mouse Genotyping Kit Hot Start Kit (Cat. KK7352; KapaBiosystems, Wilmington, USA) following the manufacturer’s instructions. PCR reaction program was 5 min 95 °C, 40× cycles 95 °C 15 s, 60 °C 15 s, 72 °C 30 s followed by 5 min 72 °C and 1 min 4 °C. PCR amplicons were visualized on 2% agarose gels containing ethidium bromide. Used oligonucleotide primers can be found in Supplementary Table 4.

### Analysis of the mouse phenotype

Mice were sacrificed by CO_2_ asphyxia, organs removed and fixed in buffered 4% formalin solution. Paraffin-embedded tissue sections were stained with hematoxylin and eosin (H&E). Blood was collected from the tail vein or by vena cava inferior puncture (terminal) and counts were determined using an Advia120 Hematology Analyzer using Multispecies Version 5.9.0-MS software (Bayer, Leverkusen, Germany). Differential blood counts were analyzed on smears stained using Wright- Giemsa staining (Hematology, University Hospital Basel). Sections were analyzed on an Olympus BX61 microscope (Tokyo, Japan) or Nikon TI (Tokyo, Japan).

### PCR analysis of cleavage of *floxed Nsd1* exon 5

Genomic DNA was extracted from total BM or flow sorting enriched myeloid, erythroid, T and B cells using the Gentra Puregene Cell kit (Qiagen/158767) according to the manufacturer’s protocol. Fifty nanograms of gDNA were amplified using the GoTaq G2 Hot Start Polymerase (Promega/M7405) under the following conditions: 94 °C 1 min, 60 °C 1 min, 72 °C 1 min for 25 cycles. PCR products were separated on a 1.5% agarose gel containing ethidium bromide. PCR primers are given in Supplementary Table 4.

### BM transplantation

Transplantations were performed using whole BM of *Nsd1*^*−/−*^ mice at indicated age. For competitive transplantation, 1 × 10^6^ total BM cells of symptomatic or asymptomatic *Nsd1*^*−/−*^ mice (CD45.2) was mixed in a 1:1 ratio with supporting BM of B6.SJL (CD45.1) donor mice and transplanted into lethally irradiated (2× 600 cGy) B6.SJL (CD45.1) recipients via tail vein.

### Analysis of mouse hematopoiesis

To obtain fetal liver cells, one male mouse was placed with two female mice during the light period and left overnight. At the indicated dates, pregnant females were sacrificed by CO_2_ asphyxia, and fetal liver cells from individual embryos were surgically isolated, minced, and passed through 70 μm cell strainer (Cat. 352350; BD, New Jersey, USA). For adult mice, total BM was harvested by crushing long bones and spine, while spleens were dissected and single-cell suspensions obtained by pressing through a 70 μm cell strainer. Red blood cells were lysed with ammonium-chloride potassium (ACK) lysis buffer (150 mM NH_4_Cl, 10 mM KHCO_3_, and 0.1 mM EDTA, pH 8.0) for 10 min on ice. Lineage depletion was achieved according to the manufacturers’ protocol of mouse hematopoietic lineage depletion kit (Cat. 130-090-858; Miltenyi Biotech, Bergisch Gladbach, Germany). Cytospin preparations of approximately 10^5^ cells were made by centrifugation for 3 min at 300 r.p.m. using a Shandon Cytospin 3 centrifuge using cytofunnel disposable sample chambers (Cat. 5991040; Thermo Fisher Scientific, Reinach, Switzerland) and non-coated cytoslides (Cat. 5991051; Thermo Fisher Scientific, Reinach, Switzerland). Cytospots were stained with Wright Giemsa solution.

### Flow cytometry

Cells in suspension were washed with FACS buffer (0.5% BSA, 1mM EDTA in PBS) and incubated with indicated antibodies for 45 min on ice, washed, and stained with 1 μg/ml DAPI (Life Technologies, Paisley, UK) in PBS. Stained cells were analyzed on a CyAn ADP analyzer (Beckman-Coulter) or LSR Fortessa (BD, New Jersey, USA). Data were analyzed with FlowJo software (Tree Star). For CD71/Ter119 staining, the preparation still contained red blood cells, for CD71/Kit/Sca-1/FcγRII/III stem and progenitor staining the red blood cells were depleted. For stem and myeloid progenitor staining, lineage-positive cells were depleted as described before. All antibodies used in this study are indicated in Supplementary Table 5. For calculating number of stem and progenitor cells in BM, lineage-marker-depleted cells were counted and absolute numbers of cells adjusted to this number. For differentiation analysis of mouse or human cells in vitro, cells were filtered, washed twice with PBS, and stained in 100 µL FACS buffer. Detailed information about the FACS gating strategies can be found in Supplementary Fig. 8.

### RT-PCR

Quantitative RT-PCR: Total RNA was extracted using the RNA Plus extraction kit (Macherey-Nagel, Düren, Germany) according to the manufacturer’s protocol. cDNA synthesis was carried out using the high capacity cDNA reverse transcription kit (Cat. 4368814; Applied Biosystems, Foster City, USA). Quantitative PCR was performed using SYBR Green reagent (Applied Biosystems, Foster City, USA) and an ABI prism 7500 sequence detection system. Ct values were normalized to *Gapdh* expression and relative expression was quantified using 1/dCt or the 2^(−ddCt)^ method^64^. Primers are given in Supplementary Table 6. For multivariate analysis of RT-qPCR the multicomp R package was used to model dCt values kept log space, extracting coefficients at each timepoint using Tukey’s test. Where specified the model included adjustment of the effect of individual mouse and plots depicts the residuals following this regression.

### Colony forming assay

For whole BM analysis, approximately 4 × 10^4^ cells were plated in methylcellulose M3434 (Methocult, StemCell Technologies, Vancouver, Canada). Colonies were scored after 8–10 days. Pictures were taken on Olympus IX50 microscope with ×2, ×4, and ×10 magnification. Cells were washed, resuspended, counted with trypan blue and if applicable replated into fresh methylcellulose. For colony formation analysis of human CD34^+^ cells, 5 × 10^3^ cells were plated into H4434 (Methocult; StemCell Technologies, Vancouver, Canada). After scoring of M3434 plates, when indicated, dishes were incubated with a mix of two volumes of 0.3% hydrogen peroxide and five volumes of 0.2% di-hydrochloride benzidine (Sigma Aldrich, Buchs, Switzerland) in 0.5 M acetic acid/1× PBS for 5 min at 37 °C.

### Western blotting

For protein detection, total cell extracts were isolated from freshly cultured 1 × 10^6^ cells using 60 μl of Laemmli sample buffer containing 20% SDS. Following 5 min boiling at 100 °C, samples were centrifuged at 4 °C for 10 min, and supernatant was placed in a new tube. Nuclear protein lysates were prepared by resuspending cells in hypotonic lysis buffer (10 mM HEPES pH 7.9, 10 mM KCl, 0.1 mM EDTA, 0.1 mM EGTA, 1 mM DTT) for 15 min on ice, followed by treatment with 0.1% NP-40 and 15 s vortexing. Nuclei were spun down at 14,000 r.p.m. for 2 min at 4 °C and supernatant containing cytoplasmic fraction kept for analysis. Pellets were resuspended in nuclear lysis buffer (20 mM HEPES pH 7.9, 0.4 M NaCl, 1 mM EDTA, 1 mM EGTA, 1 mM DTT). In addition, pellets were sonicated for three cycles (30 s sonication, 30 s pause) on a Bioruptor pico sonicator (Diagenode, Seraing, Belgium) and left for 20 min on ice before spinning down at 14,000 r.p.m. for min at 4 °C. Lysates were kept for analysis of nuclear proteins and remaining pellets used for histone extraction in 0.2 N HCl and beta-mercaptoethanol. Lysis buffers were supplemented with Complete Mini protease inhibitors (Cat. 11836153001; Roche). Proteins were quantified by Bradford assay (Biorad, München, Germany) and loading adjusted. Samples were prepared in 4× Laemmli buffer (Biorad, München, Germany) and boiled for 10 min at 95 °C before loading on pre-cast (BioRad) or hand casted gels of different percentages. For NSD1 blot, 50 μg of nuclear extract was loaded on a 5% running gel. Wet transfer was done overnight at 4 °C in 5% methanol/0.1% SDS/tris-base-bicine buffer on 0.45 µM nitrocellulose membranes. For blotting GATA1, 10 μg nuclear extract was loaded on 10% gels and semi-dry transfer was done for 30 min on nitrocellulose 0.2 µM (Biorad, München, Germany). For SKI, whole lysate from 1 MIO cells was boiled and loaded on 6–7.5% gels. Wet transfer was carried out for 3 h at 4 °C. Membranes were blocked in 5% non-fatty milk (NFM) in PBS–1% Tween for 2 h at room temperature. Blots were probed overnight with antibody at 4 °C in 2.5% NFM/PBS–1%Tween, washed three times for 15 min in PBS–1% Tween and probed with a secondary antibody in 2.5%NFM/PBS–1%Tween. Again, blots were washed three times in for 15 min in PBS–1% Tween and then probed with Supersignal West Femto Max substrate (Thermo Scientific, Reinach, Switzerland). Carestream Biomax Kodak films were used for development (Sigma, New York, USA). Uncropped original scans of the western blots membranes as shown in Figs. 5b, 6c, 8b, h are provided in Supplementary Fig. 9. Information regarding the used antibodies can be found in Supplementary Table 7.

### In vitro erythroid differentiation assay

Fetal liver and adult BM-derived erythroblasts cells were obtained following a previously published protocol^27^. Erythroblast cultures from adult mice were established after lineage depletion of BM cells, Cells were cultured for more than one week in maintenance medium composed of StemSpan SFEM (StemCell Technologies, Vancouver, Canada), supplemented with 1% Pen/Strep, 0.4% cholesterol (Gibco, Thermo Fisher Scientific, Reinach, Switzerland), 2 U/ml hEpo (Eprex 4000, 9096976, Pharmacy of University Hospital Basel), 100 ng/ml mScf (Peprotech, London, UK), 10^−6^ M dexamethasone (Calbiochem, Sigma Aldrich, Buchs, Switzerland), and 40 ng/ml hIGF-1 (Peprotech, London, UK). Cells were split every second day and presence of proerythroblasts was verified by flow cytometry (DAPI^−^/FSC^+^/CD71^+^/Ter119^−^) and cytospins. Erythroblasts were subjected to terminal maturation in differentiation medium composed of IMDM (Gibco, Thermo Fisher Scientific, Reinach, Switzerland), 1% P/S, 10% FCS, 10% PFHMII (Gibco, Thermo Fisher Scientific, Reinach, Switzerland), 5% hPDS (0.45 µM filtered, Blood donation service, University Hospital Basel), monothioglycerol (Sigma Aldrich, Buchs, Switzerland), 100 ng/ml mSCF and 2 U/ml hEpo.

### Retroviral gene transfer

Full-length cDNAs for murine *Nsd1* (*pSG5*) was obtained from R. Losson (Strasbourg). Wild type *(Nsd1*) and a catalytically inactive (*Nsd1*^*N1918Q*^) mutant ORF were cloned into the murine stem cell virus (*pMSCV*) expression vector and sequence verified. A retrovirus (*pLMP*) encoding for an SKI-specific mir-shRNA was a gift from M. Hayman (Bufallo, NY). A full-length cDNA for murine *Gata1* was obtained from T. Mercher (Paris) and cloned into *pMSCV* and sequenced verified. Retroviral stocks were produced by transient co-transfection of packaging vectors (*pIPAK6*) and respective plasmids using Turbofect or Jetprime transfection reagent (Life Technologies, Paisley, UK) in HEK293T-LX cells kept in DMEM (Gibco, Lubio, Thermo Fisher Scientific, Reinach, Switzerland) with 10% FSC and 1% P/S. Viral supernatants were harvested 48 and 72 h after transfection, 10× Vivaspin 20 (Sartorius, Göttingen, Germany) concentrated at 4000 r.p.m. for 40 min at 4 °C and snap frozen in liquid nitrogen and stored in −80 °C until usage. Cells were spin-infected either in StemSpan SFEM, supplemented with 50 ng/ml hTPO (Peprotech, London, UK) and 50 ng/ml mSCF or in maintenance medium used for erythroblast culture as described above, in the presence of 5 µg/ml polybrene (Sigma Aldrich, Buchs, Switzerland) with virus for 90 min, 2500 rpm at 30 °C. Four hours after spin infection, the cells were washed with PBS and plated in maintenance medium. Two days after spin infection, the cells were selected with 2 µg/ml puromycin (Gibco, Thermo Fisher Scientific, Reinach, Switzerland) or EGFP^+^ cells were FACS enriched as described before. Information regarding the used plasmids can be found in Supplementary Table 8.

### Reporting summary

Further information on research design is available in the Nature Research Reporting Summary linked to this article.

## Supplementary information

Supplementary Information

Description of Additional Supplementary Files

Supplementary Data 1

Supplementary Data 2

Supplementary Data 3

Supplementary Data 4

Supplementary Data 5

Supplementary Data 6

Supplementary Data 7

Supplementary Data 8

Supplementary Data 9

Supplementary Data 10

Supplementary Data 11

Supplementary Data 12

Reporting Summary

## Data Availability

The RNA raw expression data are accessible with the following number GSE136811. The mass spectrometry proteomics data have been deposited to the ProteomeXchange Consortium via the PRIDE partner repository with the dataset identifierPXD017657 (ref. ^65^).

## References

[1] Hattangadi SM, Wong P, Zhang L, Flygare J, Lodish HF (2011). From stem cell to red cell: regulation of erythropoiesis at multiple levels by multiple proteins, RNAs, and chromatin modifications. Blood.

[2] Kerenyi MA, Orkin SH (2010). Networking erythropoiesis. J. Exp. Med..

[3] Yu M (2009). Insights into GATA-1-mediated gene activation versus repression via genome-wide chromatin occupancy analysis. Mol. Cell.

[4] Fujiwara Y, Browne CP, Cunniff K, Goff SC, Orkin SH (1996). Arrested development of embryonic red cell precursors in mouse embryos lacking transcription factor GATA-1. Proc. Natl. Acad. Sci. USA.

[5] Shimizu R (2004). Leukemogenesis caused by incapacitated GATA-1 function. Mol. Cell Biol..

[6] Boddu P (2018). Erythroleukemia-historical perspectives and recent advances in diagnosis and management. Blood Rev..

[7] Iacobucci I (2019). Genomic subtyping and therapeutic targeting of acute erythroleukemia. Nat. Genet..

[8] Huang N (1998). Two distinct nuclear receptor interaction domains in NSD1, a novel SET protein that exhibits characteristics of both corepressors and coactivators. EMBO J..

[9] Wang X (2001). Identification and characterization of a novel androgen receptor coregulator ARA267-alpha in prostate cancer cells. J. Biol. Chem..

[10] Wagner EJ, Carpenter PB (2012). Understanding the language of Lys36 methylation at histone H3. Nat. Rev. Mol. Cell Biol..

[11] Kudithipudi S, Lungu C, Rathert P, Happel N, Jeltsch A (2014). Substrate specificity analysis and novel substrates of the protein lysine methyltransferase NSD1. Chem. Biol..

[12] Dolnik A (2012). Commonly altered genomic regions in acute myeloid leukemia are enriched for somatic mutations involved in chromatin remodeling and splicing. Blood.

[13] Garg M (2015). Profiling of somatic mutations in acute myeloid leukemia with FLT3-ITD at diagnosis and relapse. Blood.

[14] Papillon-Cavanagh S (2017). Impaired H3K36 methylation defines a subset of head and neck squamous cell carcinomas. Nat. Genet..

[15] Su X (2017). NSD1 inactivation and SETD2 mutation drive a convergence toward loss-of-function of H3K36 writers in clear-cell renal cell carcinomas. Cancer Res..

[16] Peri S (2017). NSD1- and NSD2-damaging mutations define a subset of laryngeal tumors with favorable prognosis. Nat. Commun..

[17] Berdasco M (2009). Epigenetic inactivation of the Sotos overgrowth syndrome gene histone methyltransferase NSD1 in human neuroblastoma and glioma. Proc. Natl. Acad. Sci. USA.

[18] Lee ST, Wiemels JL (2016). Genome-wide CpG island methylation and intergenic demethylation propensities vary among different tumor sites. Nucleic Acids Res..

[19] Kurotaki N (2002). Haploinsufficiency of NSD1 causes Sotos syndrome. Nat. Genet..

[20] Baujat G, Cormier-Daire V (2007). Sotos syndrome. Orphanet J. Rare Dis..

[21] Park S, Supek F, Lehner B (2018). Systematic discovery of germline cancer predisposition genes through the identification of somatic second hits. Nat. Commun..

[22] Rayasam GV (2003). NSD1 is essential for early post-implantation development and has a catalytically active SET domain. EMBO J..

[23] Koulnis M (2011). Identification and analysis of mouse erythroid progenitors using the CD71/TER119 flow-cytometric assay. J. Visualized Exp..

[24] Ogilvy S (1999). Promoter elements of vav drive transgene expression in vivo throughout the hematopoietic compartment. Blood.

[25] Kingsley PD (2013). Ontogeny of erythroid gene expression. Blood.

[26] Kogan SC (2002). Bethesda proposals for classification of nonlymphoid hematopoietic neoplasms in mice. Blood.

[27] England SJ, McGrath KE, Frame JM, Palis J (2011). Immature erythroblasts with extensive ex vivo self-renewal capacity emerge from the early mammalian fetus. Blood.

[28] Ferreira R, Ohneda K, Yamamoto M, Philipsen S (2005). GATA1 function, a paradigm for transcription factors in hematopoiesis. Mol. Cell Biol..

[29] Welch JJ (2004). Global regulation of erythroid gene expression by transcription factor GATA-1. Blood.

[30] Wang GG, Cai L, Pasillas MP, Kamps MP (2007). NUP98-NSD1 links H3K36 methylation to Hox-A gene activation and leukaemogenesis. Nat. Cell Biol..

[31] Dai MS, Mantel CR, Xia ZB, Broxmeyer HE, Lu L (2000). An expansion phase precedes terminal erythroid differentiation of hematopoietic progenitor cells from cord blood in vitro and is associated with up-regulation of cyclin E and cyclin-dependent kinase 2. Blood.

[32] DeVilbiss AW, Boyer ME, Bresnick EH (2013). Establishing a hematopoietic genetic network through locus-specific integration of chromatin regulators. Proc. Natl. Acad. Sci. USA.

[33] DeVilbiss AW (2015). Epigenetic determinants of erythropoiesis: role of the histone methyltransferase SetD8 in promoting erythroid cell maturation and survival. Mol. Cell Biol..

[34] Malik J, Getman M, Steiner LA (2015). Histone methyltransferase Setd8 represses Gata2 expression and regulates erythroid maturation. Mol. Cell Biol..

[35] Zhang L (2013). ZFP36L2 is required for self-renewal of early burst-forming unit erythroid progenitors. Nature.

[36] Wu W (2011). Dynamics of the epigenetic landscape during erythroid differentiation after GATA1 restoration. Genome Res.

[37] Hattangadi SM (2014). Histones to the cytosol: exportin 7 is essential for normal terminal erythroid nuclear maturation. Blood.

[38] Shaw GC (2006). Mitoferrin is essential for erythroid iron assimilation. Nature.

[39] Aizawa S (2005). Ineffective erythropoiesis in mutant mice with deficient pyruvate kinase activity. Exp. Hematol..

[40] Gubin AN (2000). Identification of the dombrock blood group glycoprotein as a polymorphic member of the ADP-ribosyltransferase gene family. Blood.

[41] Bartunek P (2002). bFGF signaling and v-Myb cooperate in sustained growth of primitive erythroid progenitors. Oncogene.

[42] Rodriguez P (2005). GATA-1 forms distinct activating and repressive complexes in erythroid cells. EMBO J..

[43] Ueki N, Zhang L, Hayman MJ (2004). Ski negatively regulates erythroid differentiation through its interaction with GATA1. Mol. Cell Biol..

[44] Ueki N, Hayman MJ (2003). Signal-dependent N-CoR requirement for repression by the Ski oncoprotein. J. Biol. Chem..

[45] Mukai HY (2013). Establishment of erythroleukemic GAK14 cells and characterization of GATA1 N-terminal domain. Genes Cells.

[46] Moreau-Gachelin F (2006). Lessons from models of murine erythroleukemia to acute myeloid leukemia (AML): proof-of-principle of co-operativity in AML. Haematologica.

[47] Moreau-Gachelin F (1996). Spi-1/PU.1 transgenic mice develop multistep erythroleukemias. Mol. Cell Biol..

[48] Kosmider O (2005). Kit-activating mutations cooperate with Spi-1/PU.1 overexpression to promote tumorigenic progression during erythroleukemia in mice. Cancer cell.

[49] Rekhtman N, Radparvar F, Evans T, Skoultchi AI (1999). Direct interaction of hematopoietic transcription factors PU.1 and GATA-1: functional antagonism in erythroid cells. Genes Development.

[50] Choe KS (2003). Reversal of tumorigenicity and the block to differentiation in erythroleukemia cells by GATA-1. Cancer Res..

[51] Tecalco-Cruz AC, Rios-Lopez DG, Vazquez-Victorio G, Rosales-Alvarez RE, Macias-Silva M (2018). Transcriptional cofactors Ski and SnoN are major regulators of the TGF-beta/Smad signaling pathway in health and disease. Signal Transduct. Target Ther..

[52] Bonnon C, Atanasoski S (2012). c-Ski in health and disease. Cell Tissue Res..

[53] Ritter M (2006). Inhibition of retinoic acid receptor signaling by Ski in acute myeloid leukemia. Leukemia.

[54] Teichler S (2011). MicroRNA29a regulates the expression of the nuclear oncogene Ski. Blood.

[55] Feld C (2018). Combined cistrome and transcriptome analysis of SKI in AML cells identifies SKI as a co-repressor for RUNX1. Nucleic Acids Res..

[56] Muench DE (2018). SKI controls MDS-associated chronic TGF-beta signaling, aberrant splicing, and stem cell fitness. Blood.

[57] Gillespie, M. A. et al. Absolute quantification of transcription factors reveals principles of gene regulation in erythropoiesis. *Mol Cell*, 10.1016/j.molcel.2020.03.031 (2020). 10.1016/j.molcel.2020.03.031 PMC7344268 32330456

[58] Brumbaugh, J. et al. Inducible histone K-to-M mutations are dynamic tools to probe the physiological role of site-specific histone methylation in vitro and in vivo. *Nat. Cell Biol*, 10.1038/s41556-019-0403-5 (2019). 10.1038/s41556-019-0403-5 PMC6858577 31659274

[59] Weinberg DN (2019). The histone mark H3K36me2 recruits DNMT3A and shapes the intergenic DNA methylation landscape. Nature.

[60] Schulz, V. P. et al. A unique epigenomic landscape defines human erythropoiesis. Cell Rep. 28, 2996–3009 e2997 (2019). 10.1016/j.celrep.2019.08.020 PMC6863094 31509757

[61] Chiba S (1991). Establishment and erythroid differentiation of a cytokine-dependent human leukemic cell line F-36: a parental line requiring granulocyte-macrophage colony-stimulating factor or interleukin-3, and a subline requiring erythropoietin. Blood.

[62] Fagnan, A. et al. Human erythroleukemia genetics and transcriptomes identify master transcription factors as functional disease drivers. *Blood* 003062, 10.1182/blood.2019003062 (2020). 10.1182/blood.2019003062 PMC8215330 32350520

[63] Georgiades P (2002). VavCre transgenic mice: a tool for mutagenesis in hematopoietic and endothelial lineages. Genesis.

[64] Livak KJ, Schmittgen TD (2001). Analysis of relative gene expression data using real-time quantitative PCR and the 2(-Delta Delta C(T)) method. Methods.

[65] Perez-Riverol Y (2019). The PRIDE database and related tools and resources in 2019: improving support for quantification data. Nucleic Acids Res..

