# Supplementary material for: Nuclear interacting SET domain protein 1 inactivation impairs GATA1-regulated erythroid differentiation and causes erythroleukemia
Source: Nat Commun. 2020 Jun 12;11:2807. doi: 10.1038/s41467-020-16179-8 (PMC7293310; doi:10.1038/s41467-020-16179-8)
Supplement: Supplementary file 1 — Supplementary Information [file 41467_2020_16179_MOESM1_ESM.pdf]

<u>Supplementary Information</u>:

"Inactivation of the nuclear interacting SET domain protein 1 impairs GATA1-regulated erythroid differentiation and causes erythro-leukemia"

Leonards *et al*.

Supplementary figures:

Supplementary Figure 1:

NSD1 knockdown in human CD34^{+} hematopoietic cells

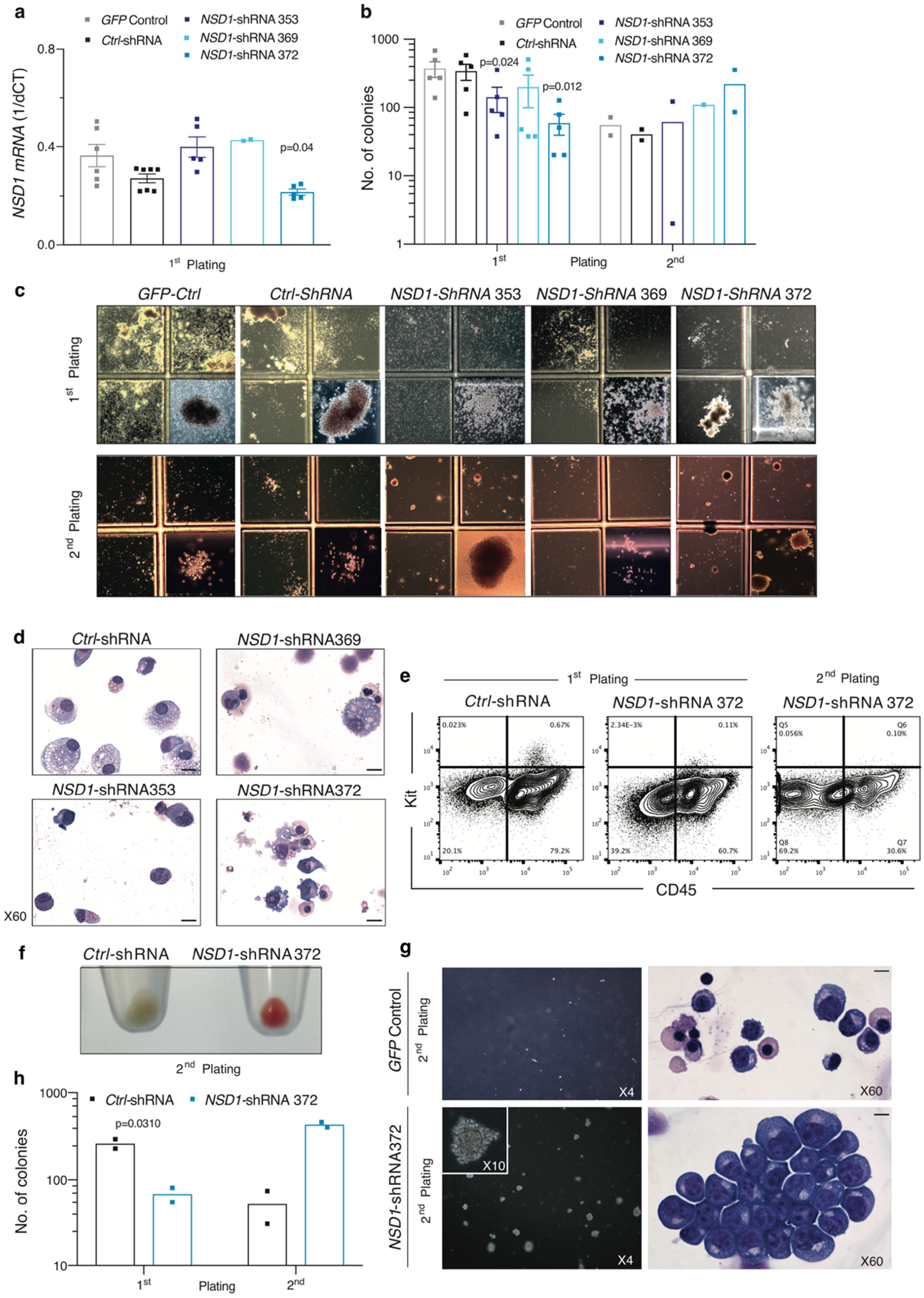

Legend to Supplementary Figure 1:

(**a**) Relative *NSD1* mRNA expression levels (1/dCT) in peripheral blood $CD34^+$ cells transduced with *pLV.PGK.GFP* (*GFP-control*, n=8), *pLKO.1*-control shRNA (*Ctrl*, n=7) or different *NSD1*-shRNAs (#353 (n=5), #369 (n=2), #372 (n=5) after the first MC plating. Bars represent average relative expression normalized to *GAPDH*.
(**b**) Number of colonies formed by $4x10^4$ peripheral blood $CD34^+$ cells transduced with *pLV.PGK.GFP* (*GFP-Control*), *pLKO.1* control shRNA (*Ctrl*-shRNA) or *pLKO.1* NSD1 (#353, #369 and #372) after the first (n=5) and the second plating (n=2) in MC (H4434).
(**c**) Representative images of first and second MC (H4434) platings of peripheral blood $CD34^+$ cells transduced with *pLV.PGK.GFP* (*GFP-control*), *pLKO.1-control* shRNA (*Ctrl*-shRNA) or different *pLKO.1 NSD1*-shRNA (#353, #369 and #372). Aberrantly large reddish colonies were mostly observed upon transduction by #353 and #369 *NSD1*shRNA expressing vectors.
(**d**) Representative images of Wright Giemsa-stained cytospin preparations of first MC (H4434) platings of peripheral blood $CD34^+$ cells transduced with *pLV.PGK.GFP* (*GFP-control*), *pLKO.1-control* shRNA (*Ctrl*-shRNA) or different *pLKO.1 NSD1*-shRNA (#353, #369 and #372), illustrating the overall predominance of cells with erythroblast morphology upon replating of cells expressing #372 and #353. (x600, size bar=10μM)
(**e**) Representative flow cytometry plots showing Kit and CD45 expression on cord blood-derived $CD34^+$ cells transduced with *pLKO.1* control shRNA (*Ctrl*-shRNA) or *pLKO.1 NSD1*-shRNA (#372) harvested from the first and second MC (H4434) plating respectively (first plating n=3, second plating n=2).
(**f**) Representative image of cell pellets formed by cord blood-derived $CD34^+$ cells transduced with *pLKO.1* control shRNA (*Ctrl*-shRNA) or *pLKO.1 NSD1* shRNA (#372) harvested from the 2nd MC (H4434) plating.
(**g**) Representative MC images (x4 & x10), and Wright-Giemsa stained cytospin preparations (x600, size bar=10μM, right panels) of cord blood-derived $CD34^+$ transduced with *pLKO.1* control shRNA (*Ctrl*-shRNA) or *pLKO.1 NSD1* shRNA (#372) from the second plating.
(**h**) Number of colonies in MC (H4434) formed by $4x10^4$ cord blood-derived $CD34^+$ cells transduced with *pLKO.1* control (*Ctrl*-shRNA) shRNA or *pLKO.1 NSD1* shRNA (#372) after the first (n=2), second plating (n=2).
Values are presented as individual points, bar graphs represent the mean value of biological replicates, error bars as standard error of the mean. Statistical significances in **a, b** and **h**, was tested with paired two-tailed t- test.

Supplementary Figure 2:

Hematopoietic ablation of *Nsd1* induces erythroleukemia in mice

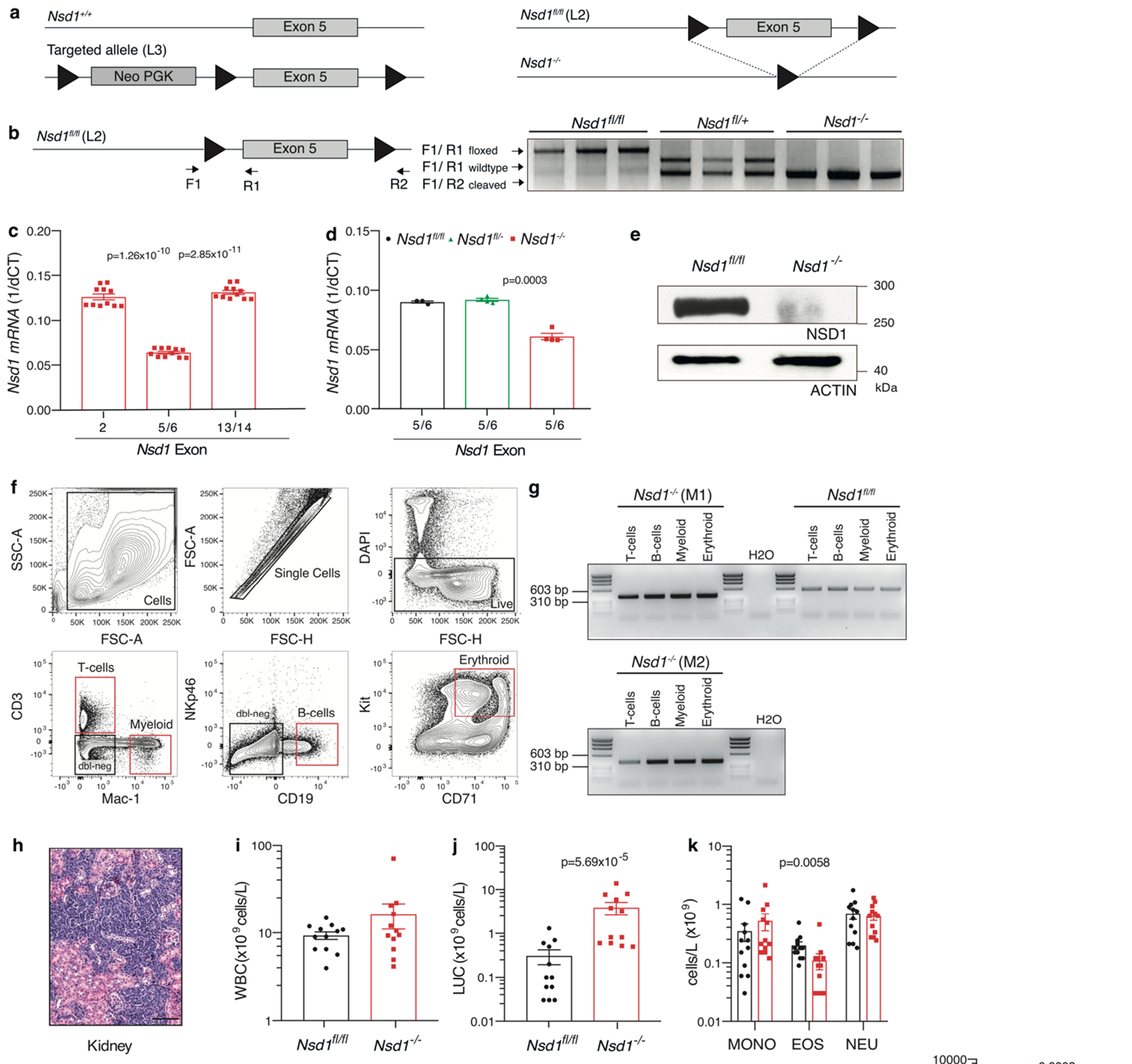

Legend to Supplementary Figure 2:

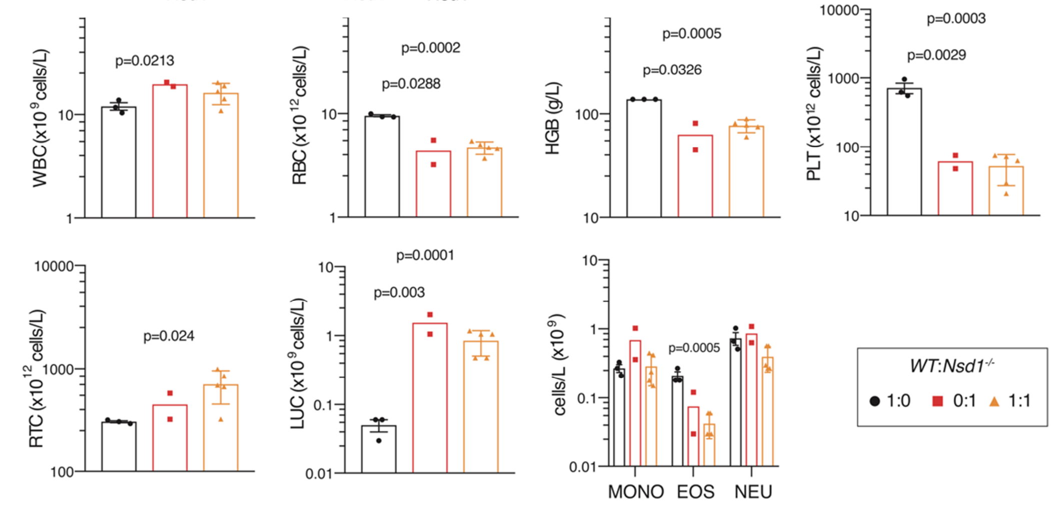

(**a**) Schematic depiction of the genetic mouse model. We first removed the *floxed* (*LoxP* sites represented by black triangles) *Neo-pgk* expression cassette from *Nsd1*$^{fl/fl(L3)}$ mice to obtain *Nsd1*$^{fl/fl(L2)}$. L2

mice were intercrossed with *Vav1-iCre* transgenic mice leading to constitutive *Nsd1* ablation in hematopoietic and endothelial cells. These mice are referred as *Nsd1*$^{-/-}$ mice.

(**b**) The PCR primer pairs F1 with R1/R2 were used to determine the presence of wildtype and the *floxed Nsd1* alleles in genomic DNA respectively.

(**c**) Relative expression of exons 2, 5/6 and 13/14 of *Nsd1* mRNA in *Nsd1*$^{-/-}$ BM cells assessed by quantitative RT-PCR analysis (1/dCt). In contrast to exon 2 (n=12) and the exon 13/14 (n=10) junction, very low levels of the exon 5/6 junction (n=19) was detected indicating almost complete excision. Bars represent average relative expression normalized to *Gapdh*.

(**d**) Relative expression of *Nsd1* exon 5 mRNA in BM cells from *Nsd1*$^{-/-}$ (n=4), *Nsd1*$^{+/-}$ (n=4) and *Nsd1*$^{fl/fl}$ (n=3) mice assessed by quantitative RT-PCR. Bars represent the average relative expression normalized to *Gapdh*.

(**e**) NSD1 protein expression in nuclear extracts of *Nsd1*$^{fl/fl}$ and *Nsd1*$^{-/-}$ spleen tissue assessed by Western blot analysis. Blots were probed with a polyclonal anti-NSD1 antibody (produced by AHFM Peters, FMI Basel). An anti-ACTIN antibody served as loading control. This data represents 1 of 3 independent experiments.

(**f**) Schematic depiction of flow cytometry gating strategies used to enrich cells (red gates) of different mature blood lineages for *Nsd1* cleavage analysis, T-cells (CD3^{+}), Myeloid cells (Mac-1^{+}), B-cells (CD19^{+}), and Erythroid cells (Kit^{+} CD71^{+}) from total BM of *Nsd1*$^{-/-}$ and *Nsd1*$^{fl/fl}$ littermate controls.

(**g**) PCR analysis for cleavage of floxed *Nsd1* exon5 in sorted cells of different mature blood lineages. The smaller PCR product indicates efficient cleavage in all lineages in 2 *Nsd1*$^{-/-}$ mice (M1 and M2, n=1), compared to a *Nsd1*$^{fl/fl}$ that showed a larger product indicating the non-cleaved floxed allele (see also Supplementary Fig. 2b).

(**h**) Representative image (x200, size bar = 50μm) of an HE-stained kidney section of a diseased *Nsd1*$^{-/-}$ mouse showing extensive leukemic cell infiltrations (n=24).

(**i**) Peripheral white cell blood counts (WBC), (**j**) "large unstained cells" (LUC) and (**k**) monocytes (MONO), eosinophils (EOS) and neutrophils (NEU) counts in diseased *Nsd1*$^{-/-}$ (red bars) and healthy *Nsd1*$^{fl/fl}$ littermates (black bars). (n=12 per group).

(**l**) Peripheral white blood counts (WBC), red cell blood counts (RBC), hemoglobin levels, platelet counts, reticulocyte counts, "large unstained cells" (LUC) and monocytes (MONO), eosinophils (EOS) and neutrophils (NEU) counts of diseased wildtype mice transplanted with control (1:0, black bars, n=2), *Nsd1*$^{-/-}$ (0:1, red bars, n=4) or in competition (1:1, orange bars, n=6).

Values are presented as individual points, bar graphs represent the mean value of biological replicates, error bars as standard error of the mean. Statistical significances in **c** was tested with paired two-tailed t- test and **d, I, j, k**, and **l** with unpaired two-tailed t- test.

Supplementary Figure 3:

Cellular and molecular characterization of the erythroleukemia-like disease of *Nsd1*$^{-/-}$ mice

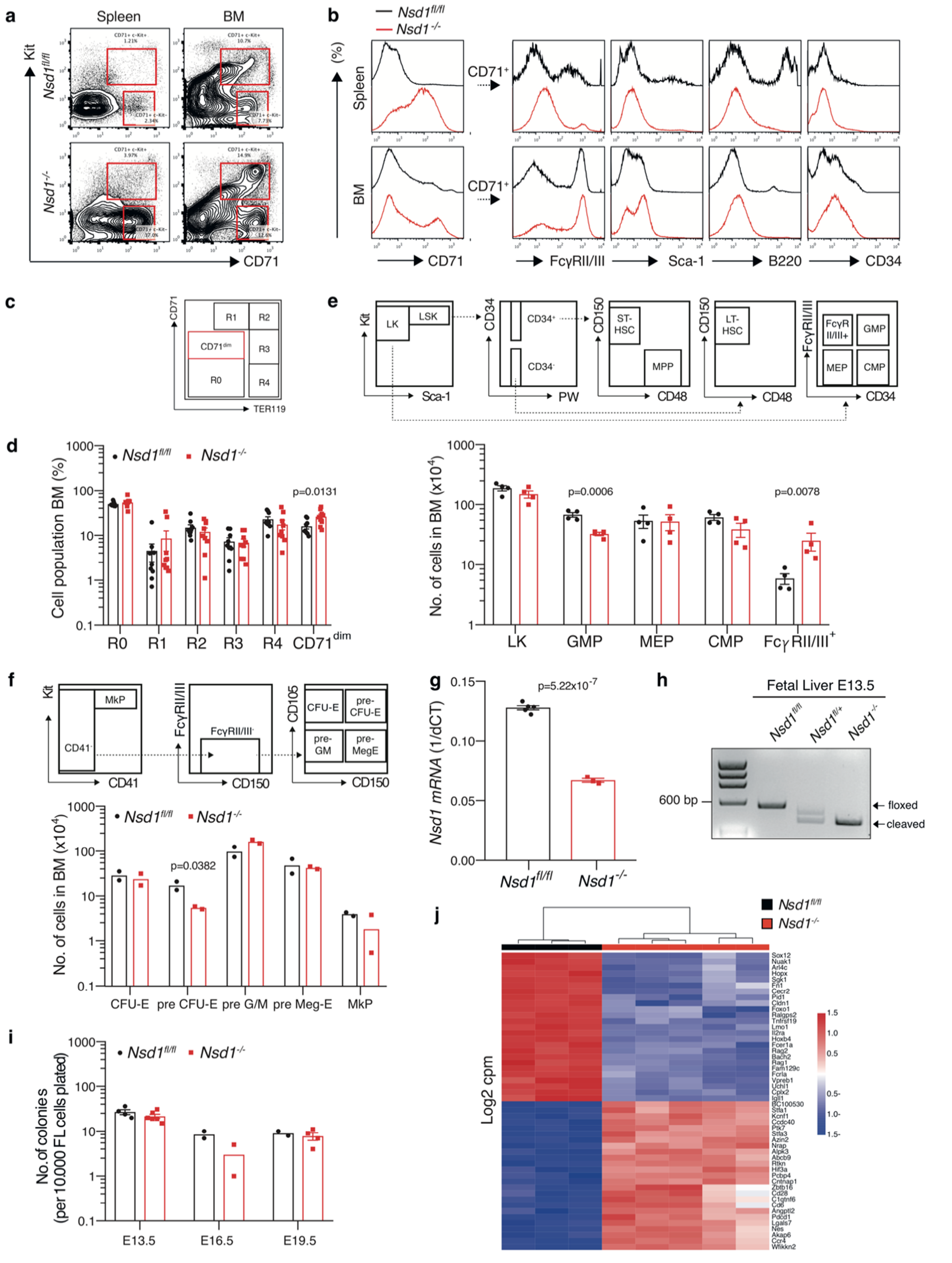

Legend to Supplementary Figure 3:

(**a**) Representative flow cytometry plots of red blood cell-lysed single cell suspensions of spleens and BM of $Nsd1^{fl/fl}$ and $Nsd1^{-/-}$ mice highlighting CD71 and Kit double positive or CD71 single positive cells.

(**b**) Representative flow cytometry histograms showing FcγRII/III, Sca-1, B220 and CD34 profiles of $CD71^{+}$ cells from spleens and BM of $Nsd1^{fl/fl}$ and $Nsd1^{-/-}$ mice.

(**c**) Flow cytometric gating strategy after CD71 and Ter119 staining to distinguish different stages of erythroid maturation: “R0” fraction ($CD71^{-/dim}$, $Ter119^{-}$), “R1” fraction ($CD71^{+}$, $Ter119^{-}$), “R2” fraction ($CD71^{+}$, $Ter119^{+}$), “R3” fraction ($CD71^{+/dim}$, $Ter119^{+}$), “R4” fraction ($CD71^{-}$, $Ter119^{+}$) and “$CD71^{dim}$” ($CD71^{+/dim}$, $Ter119^{-}$).

(**d**) Flow cytometric analysis of erythroid maturation of single cell suspensions (without red cell lysis) of BM from $Nsd1^{fl/fl}$ (black bars) and symptomatic $Nsd1^{-/-}$ (red bars) mice (n=9 per group).

(**e**) Schematic depiction of flow cytometry gating strategies to quantify $Lin^{-}/Sca\text{-}1^{+}/Kit^{+}$(LSK), $Lin^{-}/Sca\text{-}1^{+}/Kit^{+}/CD34^{-}/CD150^{+}/CD48^{-}$ (LT-HSC, long- term hematopoietic stem cells), $Lin^{-}/Sca\text{-}1^{+}/Kit^{+}/CD34^{+}/CD150^{+}/CD48^{-}$ (ST-HSC, short- term hematopoietic stem cells), $Lin^{-}/Sca\text{-}1^{+}/Kit^{+}/CD34^{+}/CD150^{-}/CD48^{+}$ (MPP, multipotent progenitor cells), $Lin^{-}/Sca\text{-}1^{-}/Kit^{+}$ (LK), $Lin^{-}/Sca\text{-}1^{-}/Kit^{+}/CD34^{+}/Fc\gamma RII/III^{+}$ (GMP, granulocyte macrophage progenitor), $Lin^{-}/Sca\text{-}1^{-}/Kit^{+}/CD34^{-}/Fc\gamma RII/III^{-}$ (MEP, megakaryocyte erythrocyte progenitor), $Lin^{-}/Sca\text{-}1^{-}/Kit^{+}/CD34^{+}/Fc\gamma RII/III^{-}$ (CMP, common myeloid progenitor) and $Lin^{-}/Sca\text{-}1^{-}/Kit^{+}/CD34^{-}/Fc\gamma RII/III^{+}$ (“$Fc\gamma RII/III^{+}$”) populations. Number of different progenitor cells ($x10^{4}$) in the BM of $Nsd1^{fl/fl}$ (black bars) and $Nsd1^{-/-}$ mice (red bars) (n=4 per group).

(**f**) Schematic depiction of flow cytometry gating strategies to quantify myeloid progenitors $Kit^{+}/CD41^{-}/Fc\gamma RII/III^{-}/CD150^{-}/CD105^{+}$ (CFU-E), $Kit^{+}/CD41^{-}/Fc\gamma RII/III^{-}/CD150^{+}/CD105^{+}$ (pre-CFU-E), $Kit^{+}/CD41^{-}/Fc\gamma RII/III^{-}/CD150^{-}/CD105^{-}$ (pre-GM), $Kit^{+}/CD41^{-}/Fc\gamma RII/III^{-}/CD150^{+}/CD105^{-}$ (pre-MegE) and $Kit^{+}/CD41^{+}/CD150^{+}$ (MkP) in BM and spleen of $Nsd1^{fl/fl}$ and $Nsd1^{-/-}$ mice. Number of different myeloid progenitor cells ($x10^{4}$) in the BM of $Nsd1^{fl/fl}$ (black bars) and $Nsd1^{-/-}$ mice (red bars) (n=2/group).

(**g**) Relative *Nsd1* (exon5/6 junction) mRNA expression in fetal liver cells from E13.5 $Nsd1^{-/-}$ (n=5) and $Nsd1^{fl/fl}$ (n=3) mice assessed by quantitative RT-PCR. Bars represent average expression normalized to *Gapdh.*

(**h**) Genomic DNA PCR analysis showing efficient cleavage of the *floxed* alleles in fetal liver tissue at E13.5 in $Nsd1^{-/-}$, $Nsd1^{fl/+}$ and $Nsd1^{fl/fl}$ mice (n=2).

(**i**) Colonies formed by $1x10^{4}$ of fetal liver hematopoietic cells in MC (M3434) from $Nsd1^{-/-}$ (red dots; E13.5: n=6, E16.5: n=2, E19.5: n=4) and $Nsd1^{fl/fl}$ (black dots; E13.5: n=4 E16.5: n=2, E19.5: n=2) mice at E13.5, E16.5 and E19.5.

(**j**) Heatmap of differently expressed genes (FDR<0.05, two-sided) in BM cells from healthy $Nsd1^{fl/fl}$ littermates (n=3) and diseased $Nsd1^{-/-}$ (n=5) mice (FDR < $4.20*10^{42}$).

Values are presented as individual points, bar graphs represent the mean value of biological replicates, error bars as standard error of the mean. Statistical significances in **d, e, f, g, i** was tested with unpaired two-tailed t-test.

Supplementary Figure 4:

Aberrant regulation of Gata1 in *Nsd1*$^{-/-}$ erythroblasts

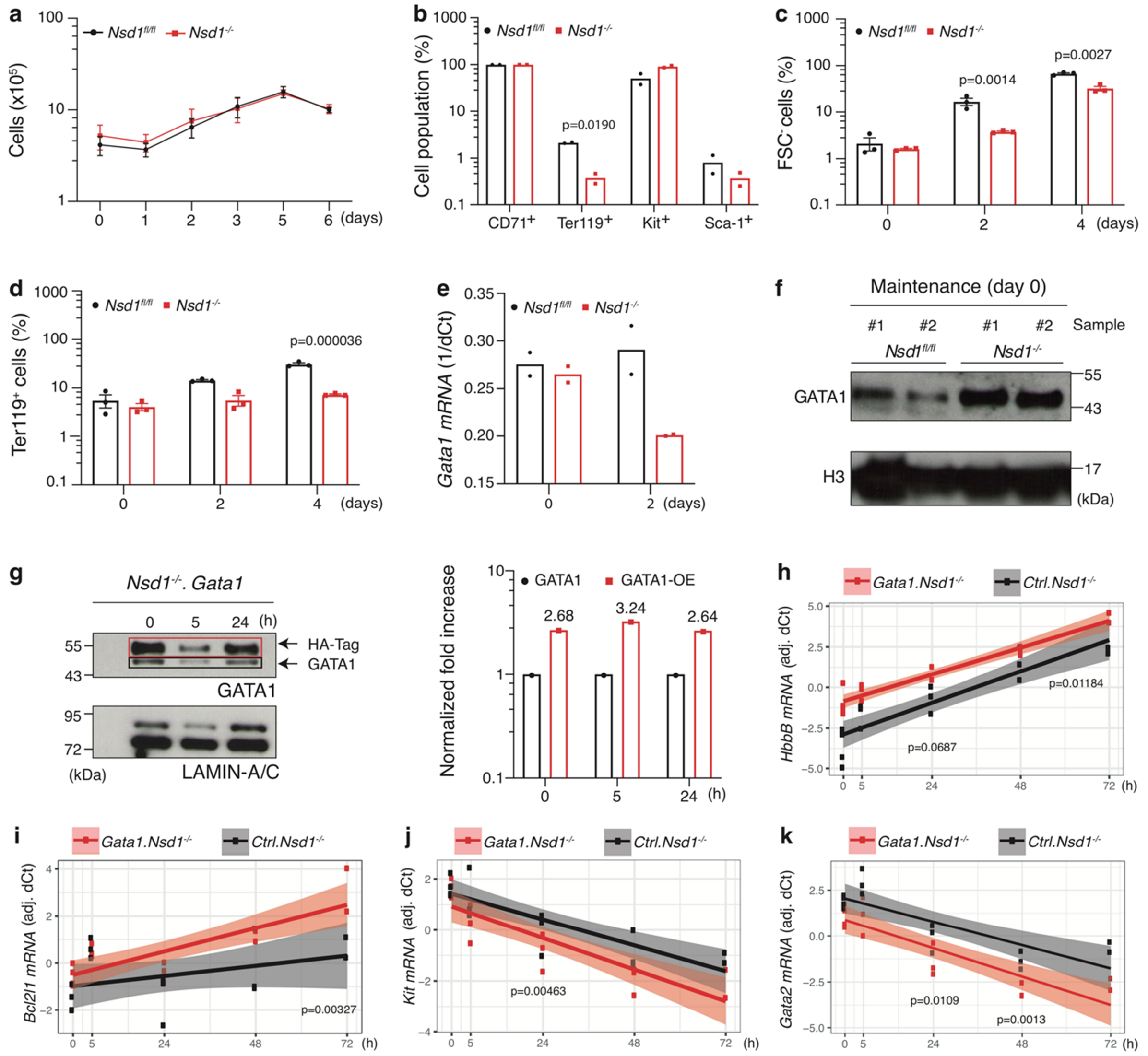

Legend to Supplementary Figure 4:

(**a**) Growth of fetal liver-derived *Nsd1*$^{fl/fl}$ (red line, n=6) and *Nsd1*$^{-/-}$ (black line, n=4) erythroblasts in maintenance medium. Living cells were counted using trypan blue exclusion. p-value > 0.05 for all time points.

(**b**) Flow cytometric CD71, Ter119, Kit and Sca-1 staining (given in %) of fetal liver-derived erythroblasts obtained from *Nsd1*$^{fl/fl}$ (black bars) and *Nsd1*$^{-/-}$ (red bars) mice grown in maintenance medium (n=2 per group).

(**c**) Forward scatter-negative (FSC^{-}) living *Nsd1*$^{fl/fl}$ (black bars) and *Nsd1*$^{-/-}$ (red bars) BM-derived erythroblasts in maintenance medium (day 0) and after 2 and 4 days in differentiation medium. (n=3 per group).

(**d**) Ter119^{+} (%) living *Nsd1*$^{fl/fl}$ (black bars) and *Nsd1*$^{-/-}$ (red bars) BM-derived erythroblasts in maintenance medium (day 0) and after 2 and 4 days in differentiation medium (n=3 per group).

(**e**) Relative *Gata1* mRNA expression (1/dCt) in *Nsd1*$^{fl/fl}$ (black bars) and *Nsd1*$^{-/-}$ (red bars) fetal liver-derived erythroblasts in maintenance medium (day 0) and after 2 days in differentiation medium

assessed by quantitative RT-PCR analysis. The Ct values were normalized to *Gapdh* expression (n=2 per group).

(**f**) GATA1 protein expression in fetal liver-derived *Nsd1*$^{fl/fl}$ and *Nsd1*$^{-/-}$ erythroblasts grown in maintenance medium as shown by Western blot analysis. H3 served as loading control (1 out of 2 experiments).

(**g**) Western blot analysis of GATA1 protein in *Nsd1*$^{-/-}$ BM-derived erythroblasts transduced with *pMSCV-mGata1-puro (Gata1)* before (0) and after 5 and 24h of induced differentiation as shown by immunoblotting. LAMIN-AC was used as loading control. The amount of exogenously expressed HA-tagged GATA1 was quantified according to band intensity (Red rectangle. bars), and calculated as fold change normalized to endogenous GATA1 expression (black rectangle, bars) using ImageJ. Numbers on top of the red bars indicate the calculated fold change increase in GATA1 expression (GATA1-OE). This data represents 1 out of 2 time course experiments.

(**h**) *HbbB* (**i**) *Bcl2l1*, (**j**) *Kit* and (**k**) *Gata2* mRNA levels in BM-derived *Nsd1*$^{-/-}$ erythroblasts transduced with *pMSCV-puro* (*Ctrl*, black dots) or *pMSCV-mGata1-puro* (*Gata1*, red dots) measured by quantitative RT-PCR analysis 0, 5, 24, 48 and 72h in differentiation medium. Values are residual ΔCT relative *Gapdh*, after adjustment for effect of individual mouse (Tukey test for difference in expression between transductions at 24h in linear model with interaction between time and transduction, adjusting for effect of mouse, two-sided, adjusted for multiple comparisons).

Values are presented as individual points, bar graphs represent the mean value of biological replicates, error bars as standard error of the mean. Statistical significances in **a, b, c, d, e, g** was tested with an unpaired two-tailed t- test.

Supplementary Figure 5:

NSD1-SET is essential for *in vitro* erythroblast maturation

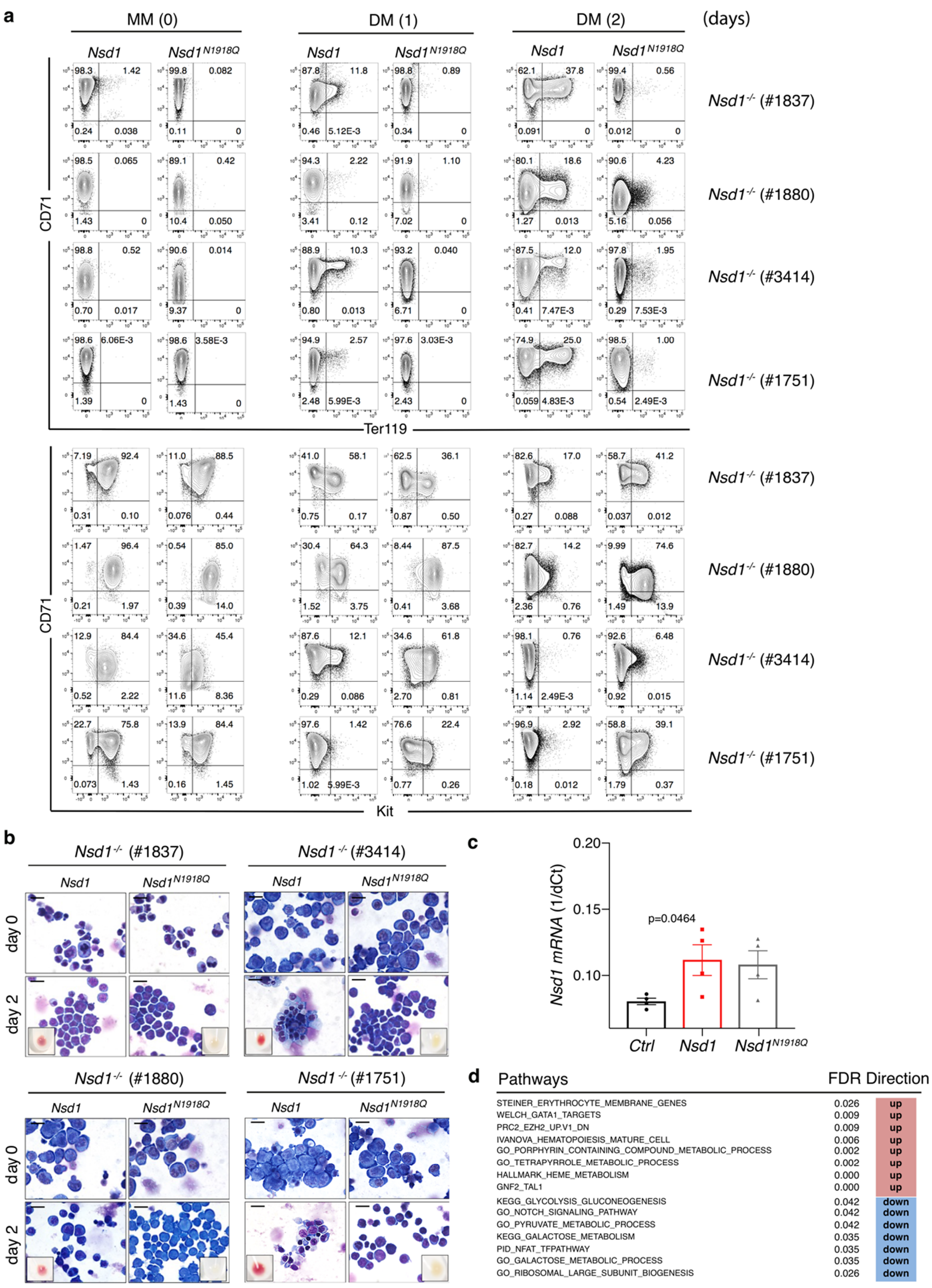

Pathways	FDR	Direction
STEINER_ERYTHROCYTE_MEMBRANE_GENES	0.026	up
WELCH_GATA1_TARGETS	0.009	up
PRC2_EZH2_UP.V1_DN	0.009	up
IVANOVA_HEMATOPOIESIS_MATURE_CELL	0.006	up
GO_PORPHYRIN_CONTAINING_COMPOUND_METABOLIC_PROCESS	0.002	up
GO_TETRAPYRROLE_METABOLIC_PROCESS	0.002	up
HALLMARK_HEME_METABOLISM	0.000	up
GNF2_TAL1	0.000	up
KEGG_GLYCOLYSIS_GLUCONEOGENESIS	0.042	down
GO_NOTCH_SIGNALING_PATHWAY	0.042	down
GO_PYRUVATE_METABOLIC_PROCESS	0.042	down
KEGG_GALACTOSE_METABOLISM	0.035	down
PID_NFAT_TFPATHWAY	0.035	down
GO_GALACTOSE_METABOLIC_PROCESS	0.035	down
GO_RIBOSOMAL_LARGE_SUBUNIT_BIOGENESIS	0.026	down

Legend to Supplementary Figure 5:

(**a**) Flow cytometry plots showing percentage of CD71$^+$, Kit$^+$ and Ter119$^+$ in living BM-derived *Nsd1*$^{-/-}$ erythroblasts transduced with *pMSCV-Nsd1-GFP-Puro* or *pMSCV-Nsd1*^{N1918Q}*-GFP-Puro* expanded in maintenance medium (day 0) and 1 or 2 days in differentiation medium. #(1837), #(1880), #(3414), and #(1751) are ID numbers of *Nsd1*$^{-/-}$ mice from which the cells were established.
(**b**) Representative images of Wright-Giemsa stained cytospin preparations and cell pellets from *Nsd1*$^{-/-}$ BM-derived erythroblasts expressing either *Nsd1* or *Nsd1*^{N1918Q} expanded in maintenance medium (day 0) and after 2 days in differentiation medium. #1837, #1880, #3414, and #1751 are ID numbers of *Nsd1*$^{-/-}$ mice from which the cells were established. This data illustrates 1 transduction experiment using 4 different mice. (1000x, bars = 10μM).
(**c**) *Nsd1* mRNA relative expression levels (1/dCt) in *Nsd1*$^{-/-}$ BM-derived erythroblasts transduced with *Nsd1* (red bar), *Nsd1*^{N1918Q} (grey bar) or control (*Ctrl*, black bar) expanded in maintenance medium (n=4 per group). Values are shown as relative expression normalized to *Gapdh*.
(**d**) The most significantly differentially expressed protein signatures (FDR ≤ 0.026) as determined by difference in corresponding protein product (measured by mass-spectrometry) in *Nsd1*$^{-/-}$ BM-derived erythroblasts cells either expressing *Nsd1*- or *Nsd1*^{N1918Q} measured after 24h in differentiation medium. Significance level at FDR<0.05, using one-sided Fisher's exact test for overrepresentation of signatures, within significant genes with positive and negative logFC, respectively. A full list is provided in Supplementary Data 9.
Values are presented as individual points, bar graphs represent the mean value of biological replicates, error bars as standard error of the mean. Statistical significances in **c** was tested with an unpaired two-tailed t-test.

Supplementary Figure 6:

Nsd1 regulates GATA1 chromatin binding, H3K36 marks, and protein interactions

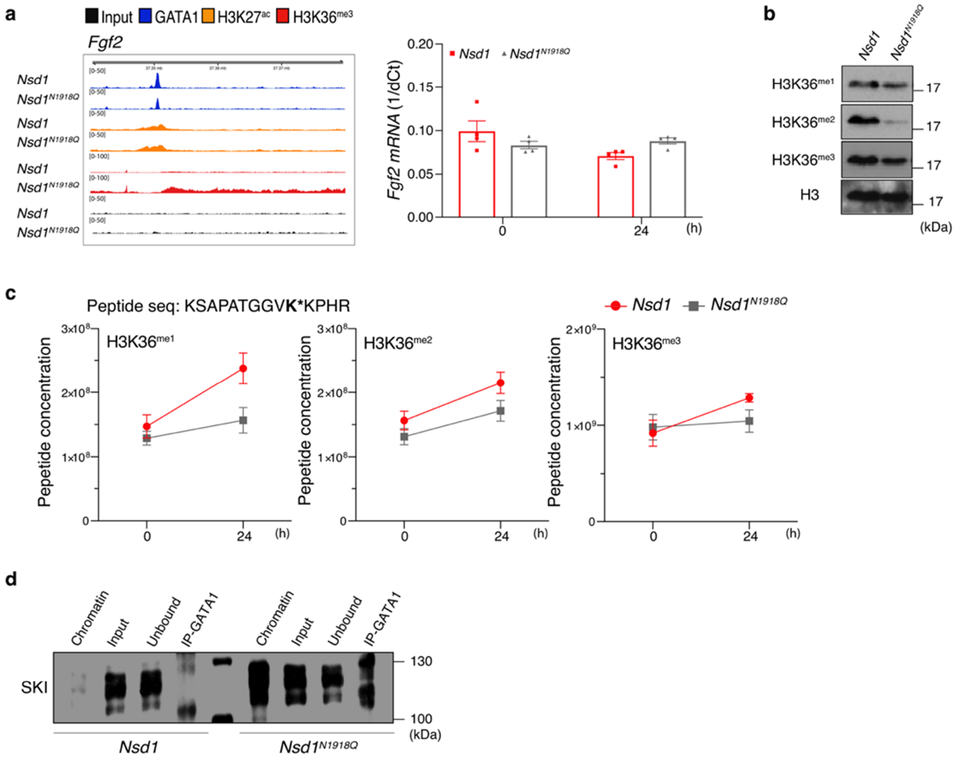

Legend to Supplementary Figure 6:

(**a**) The left panel shows an integrated genome viewer (IGV) representation of GATA1, H3K27^{ac}, and H3K36^{me3} ChIP peaks in the *Fgf2* gene locus from *Nsd1*$^{-/-}$ BM-derived erythroblasts expressing either *Nsd1* or *Nsd1*^{N1918Q} after 24h in differentiation medium (n=2). The right panel show relative *Fgf2* mRNA expression levels (1/dCt) in *Nsd1*$^{-/-}$ BM-derived erythroblasts expressing *Nsd1* (red bars) or *Nsd1*^{N1918Q} (grey bars) in maintenance medium (0h) and after 24h differentiation medium. Values are shown as relative expression normalized to *Gapdh* (n=4 per group). p-value > 0.05 for all time points.
(**b**) Western blot analysis showing H3K36^{me1}, H3K36^{me2} and H3K36^{me3} protein expression in 1x10^{6} BM-derived *Nsd1*$^{-/-}$ erythroblasts either expressing *Nsd1* or *Nsd1*^{N1918Q} grown in maintenance medium. Histone 3 (H3) was used as a loading control. This data represents 1 of 2 independent experiments.
(**c**) Comparative quantification of histone 3 lysine 36 mono, di and tri methylation (H3K36^{me1}, H3K36^{me2} & H3K36^{me3}) peptide concentration, in *Nsd1*$^{-/-}$ BM-derived erythroblasts expressing either *Nsd1* or *Nsd1*^{N1918Q} grown in maintenance medium (0h) and after 24h in differentiation medium (24h). Differentiation was associated with a higher increase in peptides containing the measured modifications in *Nsd1*- (red circles) compared to *Nsd1*^{N1918Q} expressing cells (grey squares (n=5).
(**d**) Western blot analysis showing SKI protein expression in GATA1-immunoprecipitates from nuclear extracts of 10x10^{6} *Nsd1*$^{-/-}$ BM-derived erythroblasts either expressing *Nsd1* or *Nsd1*^{N1918Q} after 24 hours in differentiation medium. This data represents 1 of 2 independent experiments.
Values in (**a**) are presented as individual points, bar graphs represent the mean value of biological replicates, error bars as standard error of the mean. Values in (**c**) are presented as a single point for the mean value of biological replicates, error bars as standard error of the mean. Statistical significances in (**a**) and (**c**) were tested with a paired two-tailed t- test.

Supplementary Figure 7:

SKI knockdown results in differentiation of *Nsd1*$^{-/-}$ erythroblasts

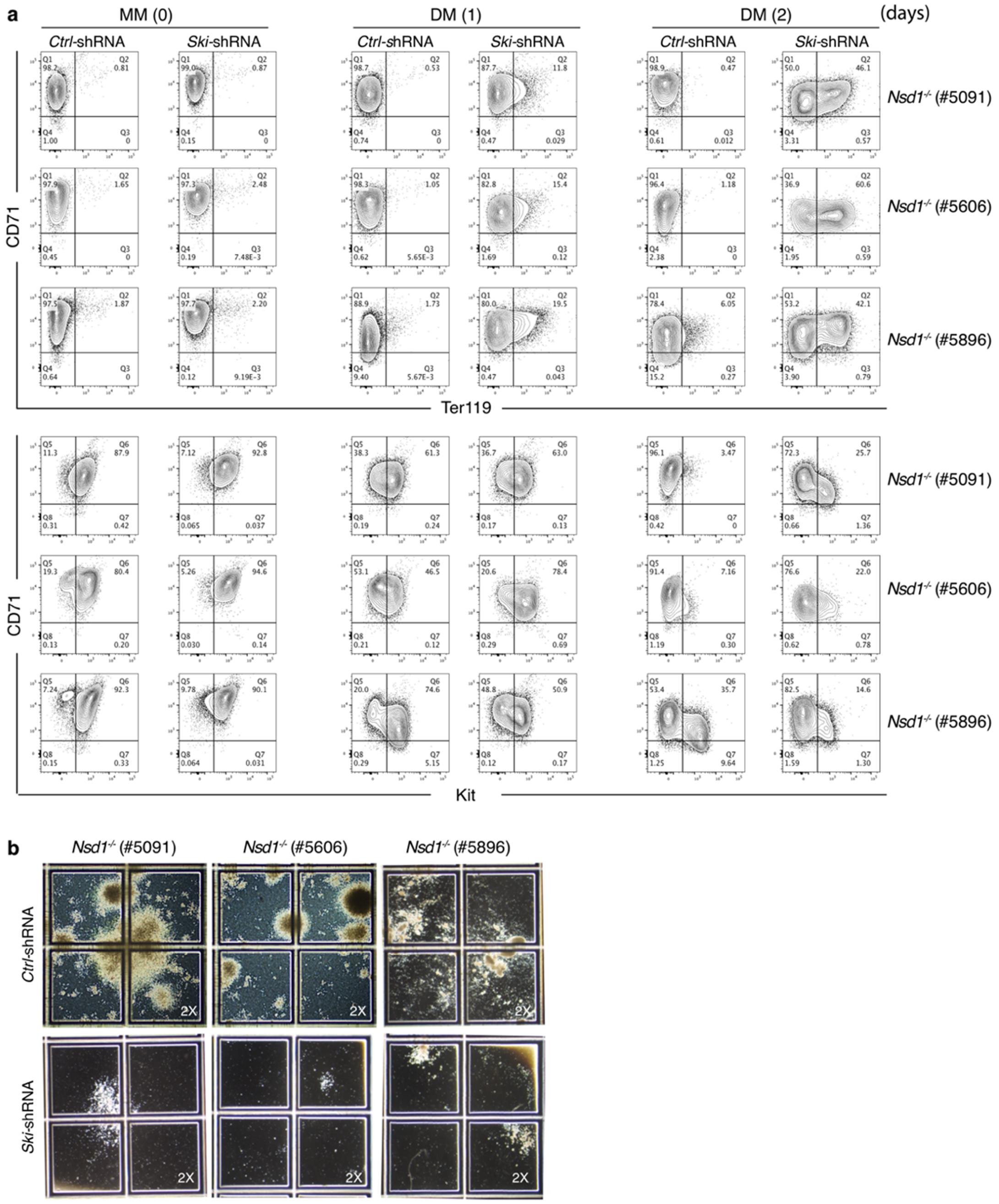

Legend to Supplementary Figure 7:

(**a**) Flow cytometry analysis showing percentage of CD71$^+$, Kit$^+$ and Ter119$^+$ stained living *Nsd1*$^{-/-}$ BM-derived erythroblasts transduced with *pLMP-empty-shRNA-GFP-Puro (Ctrl*-shRNA*),* or *pLMP-Ski-shRNA-GFP-Puro (Ski*-shRNA*)* expanded in maintenance medium (0h) and after 1 or 2 days in differentiation medium. #5091, #5606, #35896 are ID numbers of *Nsd1*$^{-/-}$ mice from which the cells were established.

(**b**) Representative images MC cultures (M3434) 11 days after plating of 1x10^4 *Nsd1*$^{-/-}$ BM-derived erythroblasts expressing either *control (Ctrl*-shRNA, top row*)* or *Ski shRNA (Ski-shRNA,* bottom row*).* #5091, #5606, #35896 indicate are ID numbers of *Nsd1*$^{-/-}$ mice from which the cells were established.

Supplementary Figure 8:

FACS gating strategies

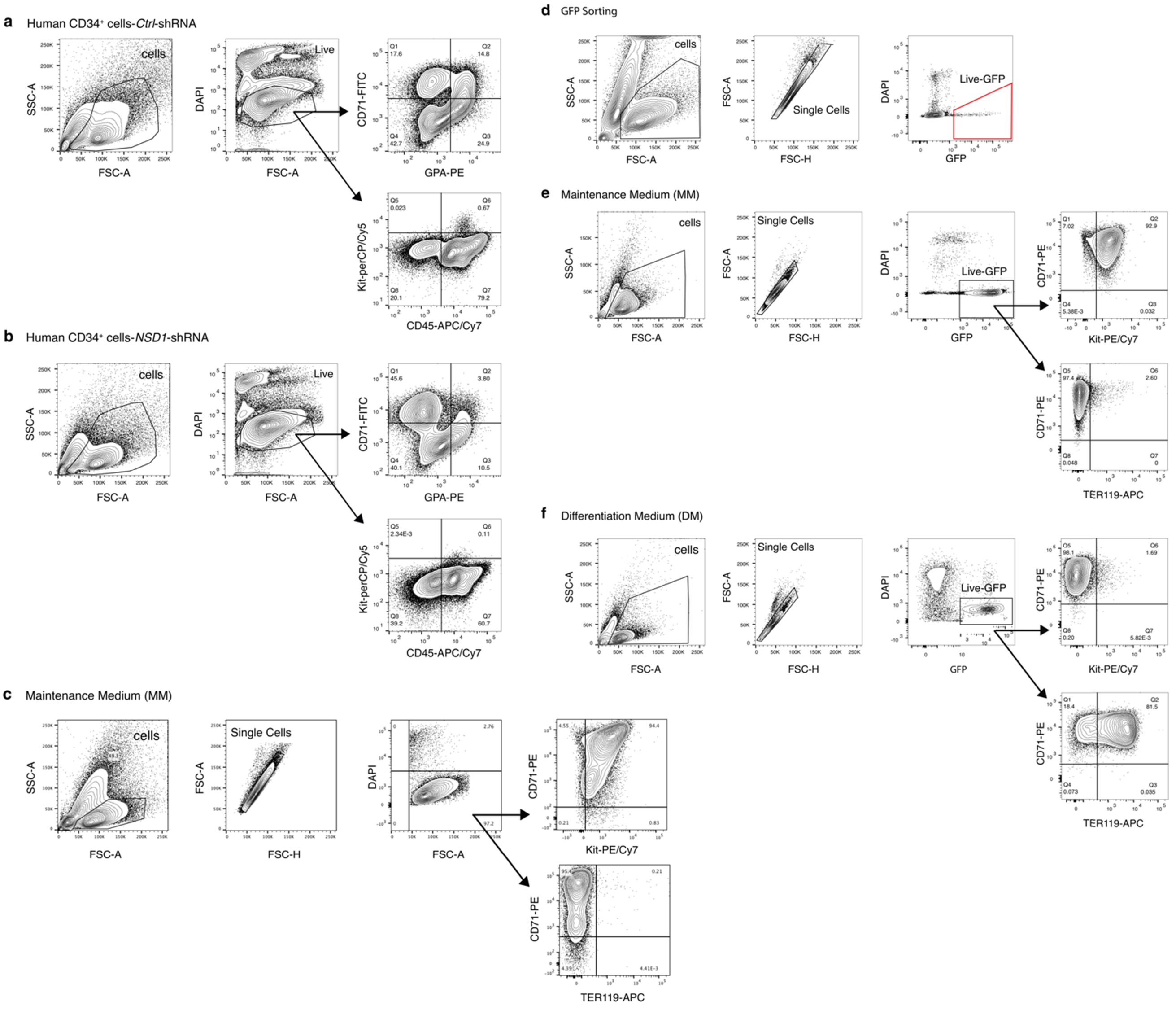

Legend to Supplementary Figure 8:

(**a-b**) Gating strategy to determine the percentage of CD71, GPA, Kit and CD45-postive cells in human CD34$^+$ cells transduced with *Ctrl*-shRNA and *NSD1*-shRNA for *in vitro* MC cultures shown in Fig. 1 & Supp. Fig.1.

(**c**) Gating strategy to determine the percentage of kit, CD71, and Ter119-positive cells in *Nsd1*$^{-/-}$ BM-derived erythroblasts transduced with *Ctrl* and *Gata1* for *in vitro* cultures presented in Fig. 4 & Supp. Fig. 4.

(**d**) Gating strategy used to sort (Red gate) *Nsd1*$^{-/-}$ BM-derived erythroblasts transduced with *Ctrl*, *Nsd1*, *Nsd1*^{N1918Q}, *Ctrl*-ShRNA and *Ski*-shRNA for *in vitro* cultures presented in Fig. 5-7 & Supp. Fig. 5-7.

(**e-f**) Gating strategy to determine the percentage of kit, CD71, and Ter119-positive cells in *Nsd1*$^{-/-}$ BM-derived erythroblasts transduced with *Ctrl*, *Nsd1*, *Nsd1*^{N1918Q}, *Ctrl*-shRNA and *Ski*-shRNA for *in vitro* cultures presented in Fig. 5-7 & Supp. Fig. 5-7.

Supplementary Figure 9:

Uncropped original Western Blot scans

Fig. 5b

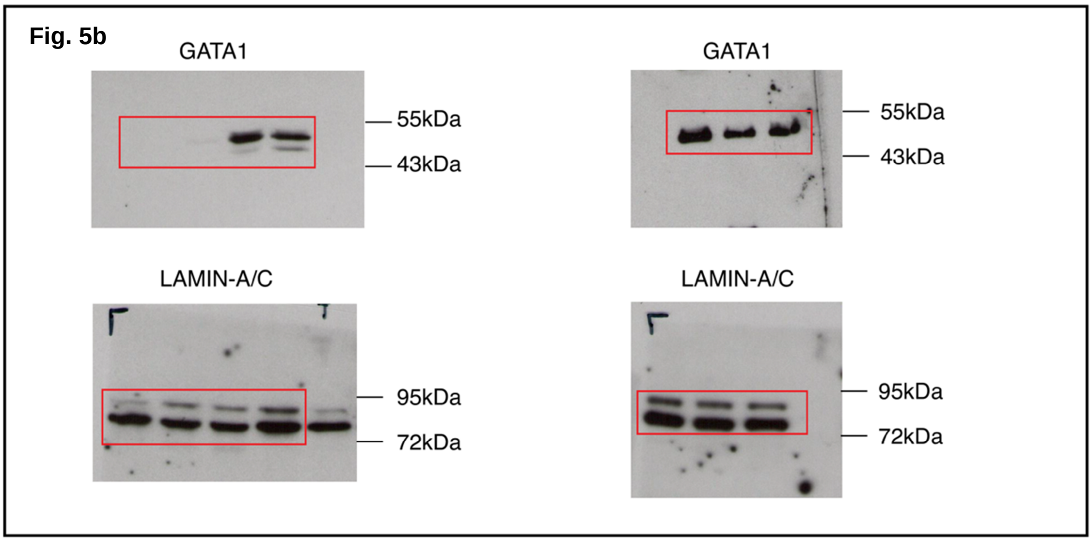

Fig. 6c

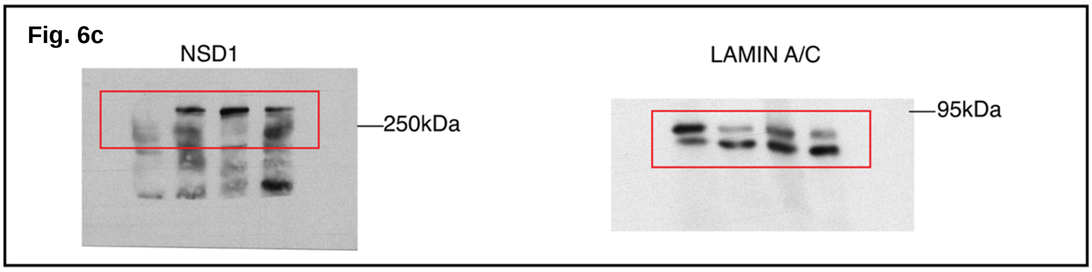

Fig. 8b **Fig. 8h**

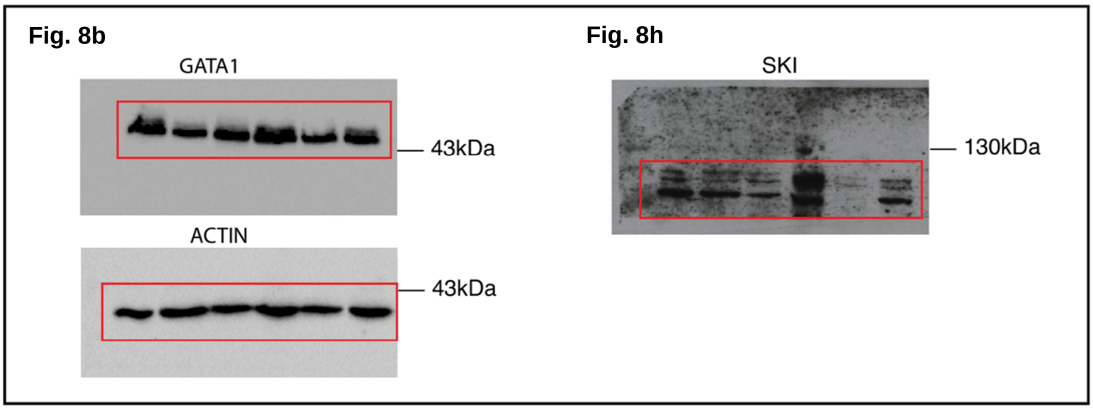

Fig. 9e

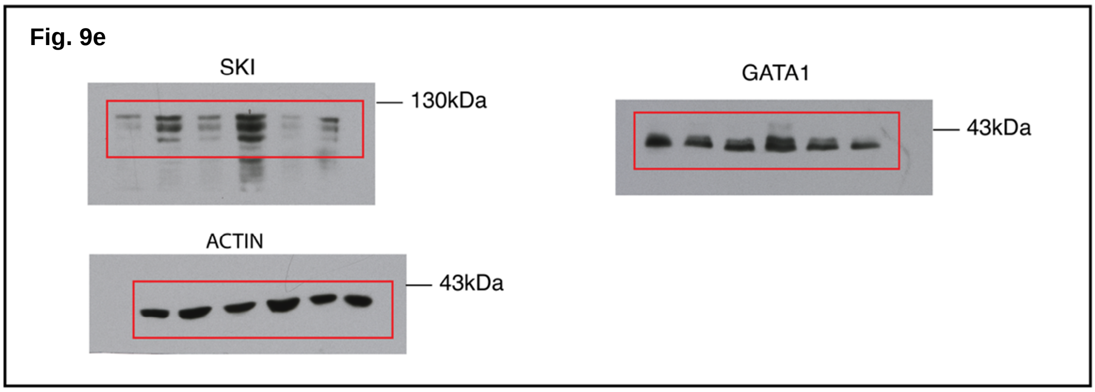

Supp. Fig. 2e

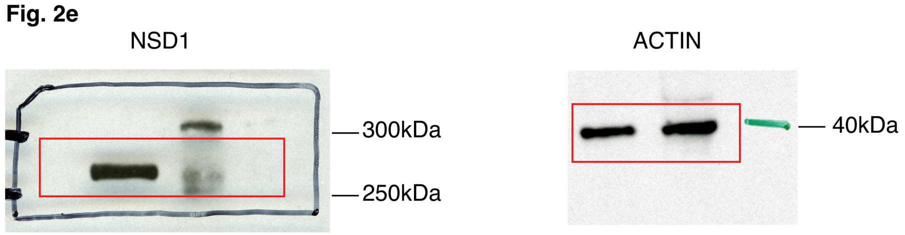

Supp. Fig. 4f

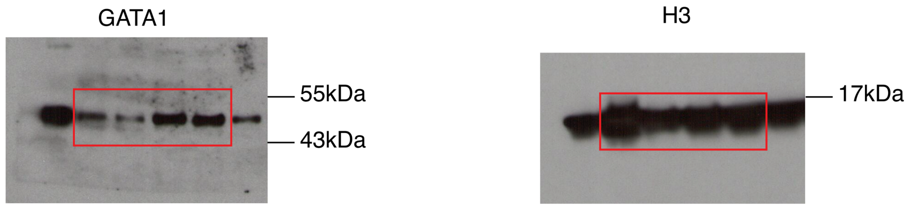

Supp. Fig. 4g

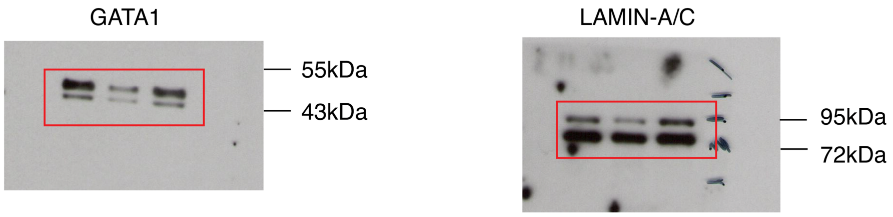

Supp. Fig. 6b

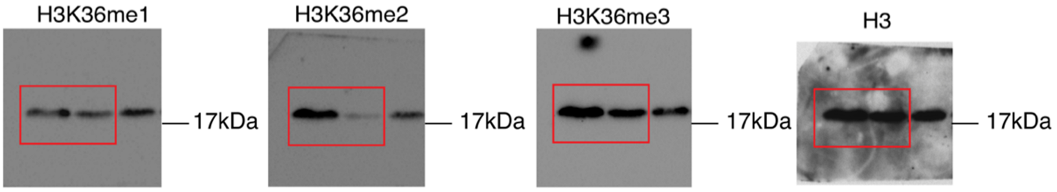

Supp. Fig .6d

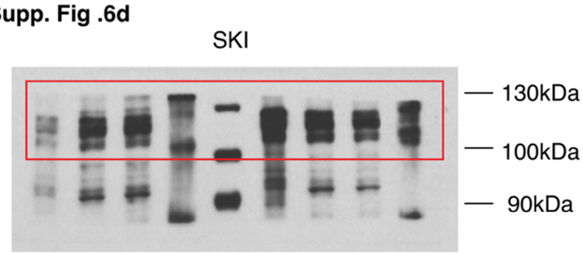

Legend to Supplementary Figure 9:

Uncropped original scans of the Western blots membranes shown in Figs. 4f, 5c, 6b and 6h.

Supplementary Tables Index:

Supplementary Table 1: Blood counts and organ weights of wildtype vs *Nsd1*$^{-/-}$ mice

Supplementary Table 2: Blood counts and organ weights from competitive BM transplantations of *Nsd1*$^{-/-}$ cells into wildtype recipients

Supplementary Table 3: Addback of SET mutated *Nsd1* into *Nsd1*$^{-/-}$ vs addback of wildtype *Nsd1* into *Nsd1*$^{-/-}$. Protein pathways analysis after 24h in differentiation medium

Supplementary Table 4: Primers for genotyping of the transgenic mice

Supplementary Table 5: Antibodies used for flow cytometric analysis and -sorting

Supplementary Table 6: RT-qPCR primers

Supplementary Table 7: Antibodies used for Western blotting, ChIP-sequencing and IP-MS experiments

Supplementary Table 8: Plasmids used for gene transfer experiments

Supplementary Table 1:

Blood counts and organ weights of wildtype vs *Nsd1*$^{-/-}$ mice (n=12)

***Nsd1*$^{fl/fl}$**												
**spleen (g)**	0.08	0.08	0.09	0.07	0.08	0.08	0.08	0.09	0.06	0.11	0.09	0.09
**liver (g)**	1.21	1.62	1.27	1.21	0.95	1.54	1.3	1.2	0.86	1.14	0.91	1.7
**RBC (x10^12 cells/L)**	9.78	10.11	8.61	9.52	10.29	9.84	10.59	9.93	9.75	9.78	10.26	9.36
**RTC (x10^12 cells/L)**	3.093	2.643	4.593	2.468	2.721	1.881	3.144	2.613	3.102	2.466	3.027	2.736
**HGB (g/L)**	138	150	126	144	150	147	156	150	147	144	156	138
**PLT (x10^12 cells/L)**	13.08	8.52	8.04	11.67	9.39	13.95	10.89	11.04	10.35	9.21	13.14	13.11
**WBC (x10^9 cells/L)**	11.88	9	10.95	12.2	14.76	3.96	9.03	5.64	10.38	6.51	6.51	10.86
**LUC (x10^9 cells/L)**	0.1	0.5	1.3	0.1	0.7	0.6	0.2	0.03	0.03	0.03	0.06	0.06
**MONO (x10^9 cells/L)**	0.24	0.33	0.24	0.03	1.08	0.06	0.18	0.06	0.15	0.09	1.23	0.45
**EOS (x10^9 cells/L)**	0.27	0.18	0.21	0.12	0.48	0.15	0.18	0.24	0.15	0.12	0.09	0.18
**NEU (x10^9 cells/L)**	0.9	0.57	1.74	0.18	1.08	0.21	0.75	0.21	0.33	0.42	0.81	0.99
***Nsd1*$^{-/-}$**												
**spleen (g)**	1.15	2.34	1.97	1.14	1.42	2.18	1.47	1.11	0.8	1.35	1.68	0.8
**liver (g)**	2.15	2.15	2.47	2.2	3.48	1.11	1.4	1.66	1.9	2.25	1.25	2.5
**RBC (x10^12 cells/L)**	5.58	5.61	5.01	8.34	8.37	5.37	9.36	6.57	7.26	5.82	7.74	8.1
**RTC (x10^12 cells/L)**	7.641	14.583	13.734	11.337	7.806	15.735	3.852	10.57	10.032	5.859	7.059	7.494
**HGB (g/L)**	75	81	84	129	117	90	144	108	117	84	120	120
**PLT (x10^12 cells/L)**	0.75	2.67	0.99	0.54	3.69	0.21	2.22	0.45	0.81	0.6	0.66	0.72
**WBC (x10^9 cells/L)**	6.51	16.35	20.55	10.56	4.14	9.27	4.95	9.42	70.08	8.49	12	21.93
**LUC (x10^9 cells/L)**	3.2	5.9	13.9	7.7	0.5	8	0.6	0.81	3.75	0.52	0.6	0.81
**MONO (x10^9 cells/L)**	2.13	0.81	0.99	0.21	0.24	0.15	0.21	0.18	0.48	0.12	0.54	0.15
**EOS (x10^9 cells/L)**	0.03	0.09	0.03	0.09	0.15	0.12	0.45	0.12	0.12	0.03	0.03	0.06
**NEU (x10^9 cells/L)**	1.11	0.93	1.29	0.42	0.75	0.33	0.63	0.27	0.69	0.27	0.63	0.24

Supplementary Table 2:

Blood counts and organ weights from wildtype mice transplanted with *Nsd1*$^{-/-}$ and wildtype BM in a competitive manner at different ratio (1:0, 0:1, 1:1)

**1:0**						
**spleen (g)**	0.1	0.09				
**liver (g)**	1	0.9				
**RBC (x10^12 cells/L)**	9.36	9.96	9.3			
**RTC (x10^12 cells/L)**	292.5	306	317.4			
**HGB (g/L)**	138	138	138			
**PLT (x10^12 cells/L)**	621	954	555			
**WBC (x10^9 cells/L)**	11.76	13.8	10.41			
**LUC (x10^9 cells/L)**	0.03	0.06	0.06			
**MONO (x10^9 cells/L)**	0.27	0.33	0.21			
**EOS (x10^9 cells/L)**	0.18	0.18	0.27			
**NEU (x10^9 cells/L)**	0.51	0.66	1.02			
**0:1**						
**spleen (g)**	0.89	1.1	1.18	1.13		
**liver (g)**	1.72	1.83	1.74	1.85		
**RBC (x10^12 cells/L)**	5.55	3.21				
**RTC (x10^12 cells/L)**	577.2	322.8				
**HGB (g/L)**	81	45				
**PLT (x10^12 cells/L)**	48	75				
**WBC (x10^9 cells/L)**	20.4	18.6				
**LUC (x10^9 cells/L)**	1.05	2.01				
**MONO (x10^9 cells/L)**	1.02	0.36				
**EOS (x10^9 cells/L)**	0.12	0.03				
**NEU (x10^9 cells/L)**	1.08	0.63				
**1:1**						
**spleen (g)**	1.2	1	1	1.34	1.07	1
**liver (g)**	2.16	1.7	1.7	1.95	1.7	2.15
**RBC (x10^12 cells/L)**	3.69	5.01	5.46	4.71	4.59	
**RTC (x10^12 cells/L)**	666.9	853.5	995.7	691.5	323.73	
**HGB (g/L)**	60	81	90	75	78	
**PLT (x10^12 cells/L)**	30	63	72	75	21	
**WBC (x10^9 cells/L)**	14.16	16.62	18.84	20.28	10.89	
**LUC (x10^9 cells/L)**	1.17	0.48	1.05	1.05	0.48	
**MONO (x10^9 cells/L)**	0.42	0.18	0.24	0.45	0.15	
**EOS (x10^9 cells/L)**	0.03	0.06	0.06	0.03	0.03	
**NEU (x10^9 cells/L)**	0.3	0.57	0.27	0.57	0.27	

Supplementary Table 3:

Addback of SET mutated *Nsd1* into *Nsd1*$^{-/-}$ vs addback of wildtype *Nsd1* into *Nsd1*$^{-/-}$.

Protein pathways analysis of cells after 24h in differentiation medium

Pathways	p-value	Direction
GO_RIBOSOMAL_LARGE_SUBUNIT_BIOGENESIS	0.025733921	down
KEGG_GALACTOSE_METABOLISM	0.035282503	down
PID_NFAT_TFPATHWAY	0.035282503	down
GSE40274_HELIOS_VS_FOXP3_AND_HELIOS_TRANSDUCED_ACTIVATED_CD4_TCELL_UP	0.035282503	down
GO_MATURATION_OF_LSU_RRNA	0.035282503	down
GO_GALACTOSE_METABOLIC_PROCESS	0.035282503	down
KEGG_GLYCOLYSIS_GLUCONEOGENESIS	0.04166082	down
GENTILE_UV_LOW_DOSE_DN	0.04166082	down
GSE40274_CTRL_VS_FOXP3_AND_EOS_TRANSDUCED_ACTIVATED_CD4_TCELL_DN	0.04166082	down
GO_ATP_GENERATION_FROM_ADP	0.04166082	down
GO_HEXOSE_CATABOLIC_PROCESS	0.04166082	down
GO_NOTCH_SIGNALING_PATHWAY	0.04166082	down
GO_PYRUVATE_METABOLIC_PROCESS	0.04166082	down
GNF2_RAD23A	0.000142273	up
GNF2_BNIP3L	0.000322478	up
GNF2_ANK1	0.000322478	up
GNF2_SPTB	0.000322478	up
GNF2_TAL1	0.000322478	up
HALLMARK_HEME_METABOLISM	0.000394452	up
GNF2_MAP2K3	0.00154909	up
GNF2_SPTA1	0.00154909	up
GO_PORPHYRIN_CONTAINING_COMPOUND_METABOLIC_PROCESS	0.00154909	up
GO_TETRAPYRROLE_METABOLIC_PROCESS	0.00154909	up
GNF2_PRDX2	0.002807792	up
GNF2_PCAF	0.006032021	up
IVANOVA_HEMATOPOIESIS_MATURE_CELL	0.006276415	up
WELCH_GATA1_TARGETS	0.008579858	up
PRC2_EZH2_UP.V1_DN	0.008579858	up
chr1q42	0.01354547	up
GO_TETRAPYRROLE_BIOSYNTHETIC_PROCESS	0.01354547	up
GNF2_CDC27	0.015598763	up
GSE34205_RSV_VS_FLU_INF_INFANT_PBMC_UP	0.015598763	up
GSE22025_UNTREATED_VS_TGFB1_TREATED_CD4_TCELL_DN	0.015598763	up
GSE5589_LPS_AND_IL10_VS_LPS_AND_IL6_STIM_MACROPHAGE_45MIN_UP	0.023343999	up
STEINER_ERYTHROCYTE_MEMBRANE_GENES	0.026248249	up
GSE42021_CD24HI_VS_CD24LOW_TREG_THYMUS_DN	0.026248249	up

Supplementary Table 4:
Primers for genotyping of transgenic mice

**Primer sequences for genotyping PCR**		
**forward:**		**reverse:**
**Vav1-iCre**	5'-CTCTGACAGATGCCAGGACA-3'	5'- TGATTTCAGGGATGGACACA-3'
**Nsd1**	5'- GTCTGCATTAAGTAATTGTGCCCTGAAG-3'	5`- ACTGACTCCTCTTCTGGAG ATCTGAGTTC-3'
**Primers sequences for cleavage PCR**		
**ZB197**		
5' – GTCTGCATTAAGTAATTGTGCCCTGAAG – 3'		
**AAW199**		
5' – ACTGACTCCTCTTCTGGAGATCTGAGTTC – 3'		
**NSD1i3R**		
5' – CACTCTCAAAGCACAGTGAGAAG – 3'		

Supplementary Table 5:
Flow cytometry and FACS antibodies

species	Antibody	Fluorochrome	Concentration	Company
human	CD45	APC-Cy7	1:100	BioLegend
human	GPA	PE	1 : 100	BioLegend
human	CD71	FITC	1 : 100	BioLegend
mouse	CD71	FITC	1 : 100	e-Bioscience
moues	CD71	PE	1 : 200	BD Pharmingen
mosue	CD71	Pe-Cy7	1 : 200	BD Pharmingen
mouse	TER119	PE	1 : 100	BD Pharmingen
mosue	TER119	APC	1:100	BD Pharmingen
mouse	c-Kit	APC	1 : 25	e-Bioscience
mouse	c-Kit	BV711	1 : 200	BioLegend
mouse	Sca-1	Pe-Cy7	1 : 25	BioLegend
mouse	FcyRII/III	PE	1 : 50	e-Bioscience
mouse	CD34	FITC	1 : 50	e-Bioscience
mouse	CD150	APC	1 : 25	e-Bioscience
mouse	CD48	A700	1 : 50	BioLegend
mouse	CD41	FITC	1 : 25	BD Pharmingen
mouse	CD105	Pe-Cy7	1 : 25	BioLegend
mouse	CD3	APC	1: 200	BD Pharmingen
mouse	CD19	APC-Cy7	1 : 200	BD Pharmingen
mouse	CD11b	FTIC	1: 200	BD Pharmingen

Supplementary Table 6:

RT-qPCR primers

target	forward 5`- 3`	reverse 5`- 3`
mGapdh	ATGACATCAAGAAGGTGGTG	CATACCAGGAAATGAGCTTG
mHbba1	TGATGTAAGCCACGGCTCTG	CAGTGGCTCAGGAGCTTGAA
mHbbb1	GTCTCTTGCCTGTGGGGAAA	CAACCAGCAGCCTGCCC
mSpi	CGATTCAGAGCTATACCAACGTCC	ACTCGTTTGTTGTGGACATGGTG
mKit	ACGATGTGGGCAAGAGTTCC	GCCTGGATTTGCTCTTTGTTGT
mBcl2l1	GCCTTTTTCTCCTTTGGCGG	TCCACAAAAGTGTCCCAGCC
mPklr	GACCGCCTCAAGGAGATGAT	CGAATGTTGGCGATGGACTC
mArt4	CGCTGTGGCTTCCAGGAG	TCTGTTCGCTACAGCCTTGG
mFgf2	GGCTGCTGGCTTCTAAGTGT	CAACTGGAGTATTTCCGTGACC
mGata1	GTGTCCTCACCATCAGATTCCAC	TCCCTCCATACTGTTGAGCAGTG
mNsd1 exon2	CAACAGCACTTGCTATGAAACAG	GCATCGTCCACACCAGTAAAA
mNsd2 exon 5/6	CAAAGAGCTCCTCCTACAAGTAAACC	CCGAATAGCTGGCTCAGGGA
mNsd1 exon 13/14	TGCTTCTAAAGGTCGTCTGATGCGC	CTAGGGGTGAAGTGATTAGGGCAGA
hGapdh	GTGGTCTCCCTGACTTTCAACAGC	A TGAGGTCCACCTGCTTGCTG
hNsd1	AGG TAC AGG AGC AGG TGC ACA	AGC ACT AGA TCG ACC TCG GGC

Supplementary Table 7:

Antibodies for Western Blotting, ChIP-Sequening and IP-MS experiments

Antibodies used in Western Blotting			
**protein**	**clone**	**dilution**	**supplier**
NSD1	# 2479	1 : 1000	gift from Antoine Peters
GATA1	D52H6 XP	1 : 1000	Cell Signaling
SKI	G-8	1 : 1000	Santa Cruz
ACTIN	C-11	1 : 6000	Santa Cruz
H3	D1H2	1 : 1000	Cell Signalng
H3K36me1	5928	1 : 1000	Cell Signalng
H3K36me2	2901	1 : 1000	Cell Signalng
H3K36me3	9050	1 : 1000	Abcam
**Antibodies for ChIP**			
GATA1	ab11862	10 ug per IP	Abcam
H3K36me3	ab9050-11	10 ug per IP	Abcam
H3K27ac	ab4729	10 ug per IP	Abcam
**Antibodies for IP-MS**			
GATA1 N6	sc-265	2ug per IP	Santa Cruz
IgG2a	sc-3883	2 ug per IP	Santa Cruz

Supplementary Table 8:

Plasmids used for gene transfer experiments

Short name	Full name
Ctrl	*pMSCV-pgk-puro*
mGata1	*pMSCV-mGata1-pgk-puro*
mNsd1	*pMSCV-mNsd1-pgk-puro-IRES-GFP*
mNsd1N1819Q	*pMSCV-mNsd1-Setmut-pgk-puro-IRES-GFP*
shRNA Ctrl	*pLKO.1 mock shRNA (Addgene plasmid 8453)*
shRNA NSD1 353	*pLKO.1 shRNA targeting NSD1 number 353 (TRCN0000061353)*
shRNA NSD1 369	*pLKO.1 shRNA targeting NSD1 number 369 (TRCN0000238369)*
shRNA NSD1 372	*pLKO.1 shRNA targeting NSD1 number 372 (TRCN0000238372)*
pIPAK6	*pIPAK6 enevlope plasmid*
Ctrl-shRNA	*pLMP-empty-shRNA-GFP-Puro*
Ski-shRNA	*pLMP-Ski-shRNA-GFP-Puro*

Supplementary methods

RNA sequencing: RNA isolation and library preparation

Total RNA was isolated from total BM of 3 diseased *Nsd1*$^{-/-}$ and 3 healthy *Nsd1*$^{fl/fl}$ littermates using the Qiagen total RNAEasy extraction kit (Cat #. 74104). Total RNA from *Nsd1* and *Nsd1*^{N198Q} transduced cells was isolated using MACHEREY-NAGEL Nucleospin RNA Plus kit (Ref 740984.250). RNA concentration was measure by Flurorometry using the QuantiFuor RNA system (cat # E3310, Promega, Mqadison, WI, USA). RNA integrity was measure using the Bioanalyzer instrument (Agilent Technologies, Santa Clara, CA, USA) using the RNA 6000 Nano Chip (Agilent, Cat #5067-1511). Library preparation was performed with 200ng total RNA using the TruSeq Stranded mRNA Library Prep Kit High Throughput (Cat # RS-122-2103, Illumine, San Diego, CA, USA). Libraries were quality-checked on the fragment Analyzer (Advanced Analytical, Ames, IA, USA) using Standard Sensitivity NSG Fragment Analysis kit (Cat # DNF-473, Advanced Analytical). Samples were pooled to equal molarity and 1.4pM was used for clustering on the NextSeq 500 instrument (D-BSSE, ETH Zurich, Basel). Samples were sequenced Single-reads 76 bases using the NextSeq 500 high Output Kit 75-cycles (Illumina, Cat # FC-404-1005). Primary data analysis was performed with the Illumina RTA version 2.4.11 and Basecalling Version bcl2fastq-2.20.0.422.

RNA-Sequencing analysis

Missing description for mapping and quantification (which parameters, genome, annotation) Quantification of reads was done QuasR by Log2 of counts+1 Per Milion (CPM) was used for plots of expression [65]. Differential expression was performed with DESeq2 [66]. In the addback experiment paired design of donor mouse was

accounted for in the following model: ~ 0 + Addback_group + mouse. To test the additive interaction term in the addback experiment the design was: ~ mouse + timepoint + *Nsd1* + *Nsd1*: timepoint, where *Nsd1* is the mutation status in the addback (WT Nsd1, or Set domain mutated *Nsd1*). For log-fold-change-shrinkage apeglm was used; ashr algorithm was used for the Addback experiment, as the design did not allow apeglm [67, 68].

Gene set enrichment analysis was performed using fGSEA [10.1101/060012], using parameters nperm=10000 or 100000, minSize = 8, maxSize = 5000. Gene signatures were from MsigDB[https://doi.org/10.1016/j.cels.2015.12.004], Gene Ontology [69] independent studies [29, 70], and erythronDB [25]. The latter "ERYTHROID LINAGE DEVELOPMENT" was obtained using the functionality "Build Gene Lists" within an erythropoietic lineage in a comparison between proerythroblasts and basophilic erythroblasts. The gene list can be found in SupplementaryData 12.

Immunoprecipitation-MS analysis of GATA1

Prior to nuclear extract preparation, all buffers were supplied with Complete Mini proteinase inhibitor (Cat. 11836153001, Roche) to prevent degradation of the target protein. In order to lyse the cell membrane, dry pellets (10 x106 cells) from *Nsd1* and *Nsd1*^{N198Q} transduced cells were incubated in hypotonic lysis buffer (10 mM HEPES; pH 7.9, 10 mM KCl; 1.5 mM $MgCl_2$) for 15 minutes on ice. After short centrifugation at 14000 rpm at 4°C the nuclear membrane was dissolved in non-ionic nuclear lysis buffer (10 mM Tris pH 7.5, 100 mM NaCl, 2.5 mM $MgCl_2$, 0.5% Triton X-100) to minimize protein denaturation. In addition, cell pellets were disrupted by benzonase nuclease (Cat. E1014-25KU, Sigma Aldrich, Buchs, Switzerland) and sonicated for 5 cycles (30 sec sonication, 30 sec pause) on a Bioruptor pico sonicator (Cat.

B01060001, Diagenode, Seraing, Belgium). Next, cells were incubated on ice for 1.5 hours. After centrifugation for 15 minutes at 4000 rpm at 4°C the supernatant containing the nuclear fraction was pre-cleared for 1h at 4°C to remove non-specific contaminants bound to the Protein G dynabeads (Cat. 1004D, Thermo Scientific, Rheinach, Switzerland). The protein concentration was determined with a colorimetric protein assay, based on the Bradford method (595 nm wavelength). The antibodies against GATA1 N6 (Cat. Sc-265, Santa Cruz) or control IgG2a (Cat. Sc-3883, Santa Cruz) (Supplementary Table 7) were pre-bound to the beads rotating for 15 minutes at room temperature to minimize co-elution of the antibodies. Immunoprecipitations were performed overnight at 4°C. The bead-antibody-antigen complexes were washed in nuclear lysis buffer to remove potential contaminants. Samples were subjected to immunoblotting or trypsin-based (5 µg/ml, Promega) on-bead digestion in 1.6 M urea/ 100 mM ammonium bicarbonate buffer at 27°C for 30 minutes. Cells were subjected to on-bead digestion. Carbamidomethylation of cysteins was performed by addition of 10 mM TCEP and 15 mM Chloroacetamide in the dark at 37 C for 1 hour. Final digestion of proteins was performed by addition of 1 µg Trypsin (Promega) at 37 C overnight. The tryptic digest was acidified by addition of TFA (pH<3) and samples were desalted using C18 reversed phase spin columns (Harvard Apparatus) following the manufacturer instructions. Dried peptides were stored at -20°C until further use. Peptides were dissolved in 0.1% formic acid prior to injection into the mass spectrometer.

Chromatin immunoprecipitation and sequencing (ChIP-seq)

ChIP protocol was adapted from the EZ-Magna ChIP^{TM} A/G kit protocol (Millipore, Merck KGaA, Darmstadt, Germany). Cells transduced with *Nsd1* or *Nsd1*^{N198Q}

pMSVC virus in maintenance as well as 24h differentiation were fixed with 1% formaldehyde, lysed with Cell Lysis and then Nuclear Lysis buffers to a concentration of 20^106 cells per mL, and finally sonicated (20-min cycle on Covaris apparatus; KBioscience). Sheared chromatin was immunoprecipitated overnight using the following antibodies: anti-GATA1 (Abcam, ab11852), anti-H3K36me3 (Abcam, ab9050-100), anti-H3K27ac (Abcam, ab4729) (Supplementary Table 7). 1/10 of the sheared chromatin was used as a reference (Input). Immune complex collection was performed using Protein G Sepharose (Sigma-Aldrich, P3296) for 1h30 at 4°C. Rinses were done according to Magna ChIP™ kit protocol with Low salt, High salt and LiCl immune complex wash buffers. Finally, elution was performed according the IPure Kit protocol (Diagenode, Cat.No. C03010012) following manufacturer's instructions. Two independent ChIP-seq experiments were conducted for Gata1, H3K27ac and H3K36me3.

Mass spectrometry analysis and label-free quantification

Protein samples from were subjected to LC–MS analysis using a dual pressure LTQ-Orbitrap Elite mass spectrometer (Thermo Fisher Scientific) connected to an electrospray ion source (Thermo Fisher Scientific) as recently described [71]. Peptide separation was carried out on an EASY nLC-1000 system (Thermo Fisher Scientific) equipped with a RP-HPLC column (75 μm × 30 cm) packed in-house with C18 resin (ReproSil-Pur C18–AQ, 1.9 μm resin, Dr. Maisch GmbH). A step-wise gradient from 95% solvent A (0.1% formic acid) and 5% solvent B (80% acetonitrile, 0.1% formic acid) to 50% solvent B over 60 min at a flow rate of 0.2 μl/min was used. Data acquisition mode was set to obtain one high resolution MS scan in the FT part of the mass spectrometer at a resolution of 240,000 full width at half-maximum (at m/z 400)

followed by 20 MS/MS scans (TOP20) in the linear ion trap of the most intense ions using rapid scan speed. Unassigned and singly charged ions were excluded from analysis. Dynamic exclusion duration was set to 30 seconds.

MS1-based label-free quantification of MS data was performed using Progenesis QI software (Nonlinear Dynamics (Waters), version 2.0). After MS raw file import the data was analyzed using default parameter settings. MS/MS-data were exported from Progenesis QI (MGF format) and searched with a target/decoy-based strategy against a database containing forward and reverse sequences of the proteome from *Mus musculus* (UniProt, 33984 entries) using MASCOT (version 2.4.1). Search criteria required strict trypsin allowing for three missed cleavages. Carbamidomethyl of cysteine was specified in Mascot as a fixed modification. Oxidation of methionine and acetyl of the n-terminus were specified in Mascot as variable modifications. Mass tolerance was set to 10 ppm for precursor ions and 0.6Da for fragment ions. The peptide and protein false discovery rate (FDR) was set to 1%. Results from the database search were imported into Progenesis QI and the resulting peptide measurement list containing peak area values of identified peptides was further used in quantitative analysis. Processing and statistical evaluation of peptide and protein quantities between samples was performed using SafeQuant [71]. Normalized peptide and protein intensities from SafeQuant analysis were used to calculate intensity ratios between experimental conditions for identified proteins. Functional analysis was performed on the corresponding gene annotation, with a one-sided Fishers' exact test for enrichment of signatures in the up- and down- regulated proteins, respectively, using adjustment for multiple hypothesis testing with Benjamini-Hochberg method (FDR).

ChIP-seq data analysis

Reads were aligned with bowtie2 (version 2.3.2) to the mouse genome (UCSC version mm10) [72]. The output was sorted and indexed with samtools (version 1.7.20) and duplicated reads were marked with picard (version 2.9.2) Coverage tracks per sample were generated by tiling the genome in 20bp windows and counting 5'end of reads per window using the function bamCount from the bioconductor package bamsignals (bioconductor version 3.6). These window counts were exported in bigWig format using the bioconductor package rtracklayer.

Peak Calling

Fragment size was estimated using the correlateReads function from csaw (bioconductor version 3.6) using data from chr1 and excluding duplicated reads [73]. The estimated average fragment size (i.e. distance of highest correlation of reads on positive and negative strand) was calculated for each experiment and between 120 and 230. For each group of biological replicates, peaks where called with macs2 (version 2.1.1) [74] using the options '-q 0.05 --nomodel --extsize 133 -g 2652783500 --keep-dup all'. Called peaks were subsequently filtered for a log2[fold-change]>1.5 and an FDR<0.05. The filtered peaks were converted to bigBed format using the UCSC command line tool bedToBigBed. Additionally, peaks were annotated using the refGene transcript annotation (version from December 18, 2015). For the filtered peak set, the fraction of peaks overlapping with annotated promoter regions, genes (intro/exons) and intergenic regions and their respective enrichment assuming a random distribution was calculated. Overlap with differentially expressed genes was calculated from transcription start sites (TSS) +/- 200 base pairs (bp). Differential binding analysis was performed with csaw using two replicates and no input.

Parameters were 'max.frag=600, pe="both", discard=repeats' and combining windows less than 1000 bp apart and annotated using window overlap to TxDb.Mmusculus.UCSC.mm10.knownGene. An initial window.width of 10 was used for GATA1 and 150 for the histone. Visualization of bigWig files was done in IGV [75].

Supplementary methods references

65 Gaidatzis, D., Lerch, A., Hahne, F. & Stadler, M. B. QuasR: quantification and annotation of short reads in R. Bioinformatics 31, 1130-1132, doi:10.1093/bioinformatics/btu781 (2015).

66 Love, M. I., Huber, W. & Anders, S. Moderated estimation of fold change and dispersion for RNA-seq data with DESeq2. Genome Biol 15, 550, doi:10.1186/s13059-014-0550-8 (2014).

67 Zhu, A., Ibrahim, J. G. & Love, M. I. Heavy-tailed prior distributions for sequence count data: removing the noise and preserving large differences. Bioinformatics 35, 2084-2092, doi:10.1093/bioinformatics/bty895 (2019).

68 Stephens, M. False discovery rates: a new deal. Biostatistics 18, 275-294, doi:10.1093/biostatistics/kxw041 (2017).

69 The Gene Ontology, C. Expansion of the Gene Ontology knowledgebase and resources. Nucleic Acids Res 45, D331-D338, doi:10.1093/nar/gkw1108 (2017).

70 Riz, I. et al. TLX1/HOX11-induced hematopoietic differentiation blockade. Oncogene 26, 4115-4123, doi:10.1038/sj.onc.1210185 (2007).

71 Glatter, T. et al. Large-scale quantitative assessment of different in-solution protein digestion protocols reveals superior cleavage efficiency of tandem Lys-C/trypsin proteolysis over trypsin digestion. J Proteome Res 11, 5145-5156, doi:10.1021/pr300273g (2012).

72 Langmead, B. & Salzberg, S. L. Fast gapped-read alignment with Bowtie 2. Nat Methods 9, 357-359, doi:10.1038/nmeth.1923 (2012).

73 Lun, A. T. & Smyth, G. K. csaw: a Bioconductor package for differential binding analysis of ChIP-seq data using sliding windows. Nucleic Acids Res 44, e45, doi:10.1093/nar/gkv1191 (2016).

74 Zhang, Y. et al. Model-based analysis of ChIP-Seq (MACS). Genome Biol 9, R137, doi:10.1186/gb-2008-9-9-r137 (2008).

75 Robinson, J. T. et al. Integrative genomics viewer. Nat Biotechnol 29, 24-26, doi:10.1038/nbt.1754 (2011).
