# Supplementary material for: Nuclear interacting SET domain protein 1 inactivation impairs GATA1-regulated erythroid differentiation and causes erythroleukemia
Source: Nat Commun. 2020 Jun 12;11:2807. doi: 10.1038/s41467-020-16179-8 (PMC7293310; doi:10.1038/s41467-020-16179-8)
Supplement: Supplementary file 2 — Description of Additional Supplementary Files [file 41467_2020_16179_MOESM2_ESM.pdf]

Description of Additional Supplementary Files

File Name: Supplementary Data 1
Description: Differential mRNA expression in wildtype vs *Nsd1*$^{-/-}$ BM erythroblasts

File Name: Supplementary Data 2
Description: Addback of SET mutated Nsd1 into *Nsd1*$^{-/-}$ vs addback of wildtype *Nsd1* into *Nsd1*$^{-/-}$. Differential mRNA expression after 24h in differentiation medium

File Name: Supplementary Data 3
Description: Gene Set Enrichment Analysis (GSEA) of significantly up- or down regulated genes according to shrunken log fold changes between maintenance medium (0h) and differentiation medium (24h) of cells rescued with add back of wildtype *Nsd1*

File Name: Supplementary Data 4
Description: Gene Set Enrichment Analysis (GESA) of significantly up- or down regulated genes according to shrunken log fold changes between wildtype add back of *Nsd1* and add back of SET mutated *Nsd1* at 24h in differentiation medium

File Name: Supplementary Data 5
Description: Addback of SET mutated *Nsd1* into *Nsd1*$^{-/-}$ vs addback of wildtype *Nsd1* into *Nsd1*$^{-/-}$. Differential expression of protein after 24h in differentiation medium

File Name: Supplementary Data 6
Description: Addback of SET mutated *Nsd1* into *Nsd1*$^{-/-}$ vs addback of wildtype *Nsd1* into *Nsd1*$^{-/-}$. Differential binding of GATA1 ChIP-seq after 24h in differentiation medium

File Name: Supplementary Data 7
Description: Addback of SET mutated *Nsd1* into *Nsd1*$^{-/-}$ vs addback of wildtype *Nsd1* into *Nsd1*$^{-/-}$. Differential binding of H3K36me3 ChIP-seq after 24h in differentiation medium

File Name: Supplementary Data 8
Description: Addback of SET mutated *Nsd1* into *Nsd1*$^{-/-}$ vs addback of wildtype *Nsd1* into *Nsd1*$^{-/-}$. Differential binding of H3K27ac ChIP-seq after 24h in differentiation medium

File Name: Supplementary Data 9
Description: Addback of SET mutated *Nsd1* into *Nsd1*$^{-/-}$ vs addback of wildtype functional *Nsd1* into *Nsd1*$^{-/-}$. Differential protein expression following IP of GATA1 after 24h in differentiation medium (normalized to the IgG IP control)

File Name: Supplementary Data 10
Description: Addback of wildtype functional *Nsd1* into *Nsd1*$^{-/-}$. Differential protein expression following IP of GATA1 after 24h in differentiation medium

File Name: Supplementary Data 11
Description: Addback of SET mutated *Nsd1* into *Nsd1*$^{-/-}$. Differential protein expression following IP of GATA1 after 24h in differentiation medium

File Name: Supplementary Data 12

Description: Gene expression signature from ErythonDb: "ERYTHROID LINEAGE DEVELOPMENT” was obtained using the functionality "Build Gene Lists" within an erythropoietic lineage in a comparison between proerythroblasts and basophilic erythroblasts
