# Supplementary material for: Nuclear interacting SET domain protein 1 inactivation impairs GATA1-regulated erythroid differentiation and causes erythroleukemia
Source: Nat Commun. 2020 Jun 12;11:2807. doi: 10.1038/s41467-020-16179-8 (PMC7293310; doi:10.1038/s41467-020-16179-8)
Supplement: Supplementary file 15 — Reporting Summary [file 41467_2020_16179_MOESM15_ESM.pdf]

nature research

Corresponding author(s): Juerg Schwaller

Last updated by author(s): Mar 20, 2020

Reporting Summary

Nature Research wishes to improve the reproducibility of the work that we publish. This form provides structure for consistency and transparency in reporting. For further information on Nature Research policies, see Authors & Referees and the Editorial Policy Checklist.

Statistics

For all statistical analyses, confirm that the following items are present in the figure legend, table legend, main text, or Methods section.

n/a	Confirmed	
☐	☒	The exact sample size (*n*) for each experimental group/condition, given as a discrete number and unit of measurement
☐	☒	A statement on whether measurements were taken from distinct samples or whether the same sample was measured repeatedly
☐	☒	The statistical test(s) used AND whether they are one- or two-sided<br>*Only common tests should be described solely by name; describe more complex techniques in the Methods section.*
☒	☐	A description of all covariates tested
☐	☒	A description of any assumptions or corrections, such as tests of normality and adjustment for multiple comparisons
☐	☒	A full description of the statistical parameters including central tendency (e.g. means) or other basic estimates (e.g. regression coefficient) AND variation (e.g. standard deviation) or associated estimates of uncertainty (e.g. confidence intervals)
☐	☒	For null hypothesis testing, the test statistic (e.g. *F*, *t*, *r*) with confidence intervals, effect sizes, degrees of freedom and *P* value noted<br>*Give P values as exact values whenever suitable.*
☒	☐	For Bayesian analysis, information on the choice of priors and Markov chain Monte Carlo settings
☒	☐	For hierarchical and complex designs, identification of the appropriate level for tests and full reporting of outcomes
☐	☒	Estimates of effect sizes (e.g. Cohen's *d*, Pearson's *r*), indicating how they were calculated

Our web collection on statistics for biologists contains articles on many of the points above.

Software and code

Policy information about availability of computer code

Data collection	RNA-seq was performed on a NextSeq 500 instrument, samples were sequenced with single-read of 76 bases using the NextSeq 500 high Output Kit 75-cycles (lllumina, Cat# FC-404-1005). Primary data analysis was performed with the lllumina RTA version 2.4.11 and Basecalling Version bcl2fastq-2.20.0.422.<br>Flow cytometry data were collected using either FACS Diva or LSR II Fortessa (BD, Biosciences) and BD Cytoflex.<br>Mass-spectrometry protein data were collected following LC-MS analysis using a dual pressure LTQ-Orbitrap Elite mass spectrometer (Thermo Fisher Scientific) connected to an electrospray ion source (Thermo Fisher Scientific). Peptide separation was carried out on an EASY nLC-1000 system (Thermo Fisher Scientific)
Data analysis	Statical analysis was performed using Prism software (GraphPad, version 7) or R (version 3.6)<br>Flow Cytometry analysis was performed using FlowJo-Software (Version 10.0.8 or 10.2)<br>RNA-seq analysis was done using R (version 3.6). QuasR by Log2 of counts+ 1 Per Milion (CPM) for plots of expression, differential expression was performed with DESeq2(version 1.24.0), gene set enrichment analysis was performed using fGSEA (version 1.10.1) [doi:10.1101/060012]<br>Processing and statistical evaluation of peptide and protein quantities between samples was performed using SafeQuant (PMID: 23017020). MSl-based label-free quantification of MS data was performed using Progenesis QI software (Nonlinear Dynamics (Waters), version 2.0)<br>Ch IP-seq reads were aligned with bowtie2 (Langmead & Salzberg, 2012)(version 2.3.2) to the mouse genome (UCSC version mmlO). Fragment size was estimated using the correlateReads function from csaw (Lun & Smyth, 2016) (bioconductor version 3.6) using data from chrl and excluding duplicated reads.<br>Complete blood count were measured using an Advia120 Hematology Analyzer using multispecies software (version 5.9.0-MS, Bayer)

For manuscripts utilizing custom algorithms or software that are central to the research but not yet described in published literature, software must be made available to editors/reviewers. We strongly encourage code deposition in a community repository (e.g. GitHub). See the Nature Research guidelines for submitting code & software for further information.

Data

Policy information about availability of data

All manuscripts must include a data availability statement. This statement should provide the following information, where applicable:

- Accession codes, unique identifiers, or web links for publicly available datasets
- A list of figures that have associated raw data
- A description of any restrictions on data availability

All sequencing data generated for this publication are in the GEO under accession number GSE136811. The mass spectrometry proteomics data have been deposited to the ProteomeXchange Consortium via the PRIDE partner repository with the dataset identifier PXD017657. All other materials will be available on request to the corresponding author .

Field-specific reporting

Please select the one below that is the best fit for your research. If you are not sure, read the appropriate sections before making your selection.

☒ Life sciences ☐ Behavioural & social sciences ☐ Ecological, evolutionary & environmental sciences

For a reference copy of the document with all sections, see nature.com/documents/nr-reporting-summary-flat.pdf

Life sciences study design

All studies must disclose on these points even when the disclosure is negative.

Sample size	The number of animals per experiment are given. The number of biological replicates for each experiment is given. No Statical methods were used to predetermine the sample size. except for the GATA-1 IP-MS analysis (n=2), we used at least 3 independent biological replicates were used for grouped analysis.
Data exclusions	One pair of RNA-sequencing reads (Nsdl and Nsd1N1918Q at 24h) were excluded due to technical issues, probably poor sequencing run, identified by skewed GC distribution over reads.
Replication	All attempts at replication were successful except for the Nsd1 rescue experiments, which we could manage to reproduced in 6 out of 15 transduction experiments. This was due to the very low percentage of transduction rate (2-5%) and variability in cell viability and expansion after sorting as the cells. Every experiment was replicated at least twice.
Randomization	Mice were randomly selected for transplantation experiments. For permutation tests (GSEA) random seed in R was set to 42
Blinding	Not relevant for this study.

Reporting for specific materials, systems and methods

We require information from authors about some types of materials, experimental systems and methods used in many studies. Here, indicate whether each material, system or method listed is relevant to your study. If you are not sure if a list item applies to your research, read the appropriate section before selecting a response.

Materials & experimental systems

n/a	Involved in the study	
☐	☒	Antibodies
☐	☒	Eukaryotic cell lines
☒	☐	Palaeontology
☐	☒	Animals and other organisms
☐	☒	Human research participants
☒	☐	Clinical data

Methods

n/a	Involved in the study	
☐	☒	ChIP-seq
☐	☒	Flow cytometry
☒	☐	MRI-based neuroimaging

Antibodies

Antibodies used	FACS antibodies<br>human CD45 APC-Cy7 1 : 100 Hl30 Biolegend<br>human GPA PE 1: 100 Hl264 Biolegend<br>human CD71 FITC 1 : 100 CY1G4 Biolegend<br>mouse CD71 FITC 1: 100 Rl7217 e-Bioscience<br>mouse CD71 Pe-Cy7 1 : 200 BD Pharmingen<br>mouse TER119 PE 1 : 100 BD Pharmingen<br>mouse TER119 APC 1:100 BD Pharmingen

mouse c-Kit APC 1 : 25 2B8 e-Bioscience
mouse CD19 APC-Cy7 1 : 200 BD Pharmingen
mouse CD11b FTIC 1: 200 BD Pharmingen
mouse Sca-1 Pe-Cy7 1: 25 D7 Biolegend
mouse CD3 APC 1: 200 BD Pharmingen
mouse FcyRll/III PE 1: 50 93 e-Bioscience
mouse CD34 FITC 1 : 50 RAM34 e-Bioscience
mouse CD150 APC 1 : 25 mShad150 e-Bioscience
mouse CD48 A700 1: 50 HM48-1 Biolegend
mouse CD41 FITC 1 : 25 eBioMWReg30 BD Pharmingen
mouse CD105 Pe-Cy7 1: 25 MJ7/18 Biolegend
mouse NKp46 Pe 1:100 29A1.4 Biolegend
Antibodies used in Western Blotting
protein clone dilution supplier
NSDl 2748 1 : 1000 gift from Antoine Peters
GATAl D52H6 XP 1 : 1000 Cell Signaling
SKI G-8 1 : 1000 Santa Cruz
ACTIN C-11 1: 6000 Santa Cruz
H3 D1H2 1 : 1000 Cell Signaling
H3K36me1 5928 1 : 1000 Cell Signaling
H3K36me2 2901 1 : 1000 Cell Signaling
H3K36me3 9050 1 : 1000 Abcam
Antibodies for ChlP
GATAl ab11862 10 ug per ChlP Abeam
H3K36me3 ab9050-11 10 ug per ChlP Abeam
H3K27ac ab4729 10 ug per ChlP Abeam
Antibodies for IP-MS
GATAl N6 sc-265 2ug per IP Santa Cruz
lgG2a sc-3883 2 ug per IP Santa Cruz

Validation

All the antibodies used for FACs analysis are commercially available and are suitable for each species. Suppliers validated their antibodies my immunofluorescence staining of mouse cells followed by flow cytometry analysis and comparison with isotype control. All the Western blot antibodies, except for NSD1, were bought commercially and validated by the companies. The NSD1 Rabbit polyclonal antibody was made in house and would be available on request from Prof. Antoine Peters (FMI, Basel, Switzerland).

Eukaryotic cell lines

Policy information about cell lines

Cell line source(s)

Lenti-Xtm Hek293T-LX obtained from Takara (cat. # 632180)

Authentication

The cell line was not authenticated

Mycoplasma contamination

The cells were not regularly tested for Mycoplasma.

Commonly misidentified lines
(See ICLAC register)

No commonly misindentified cell lines were used in the study

Animals and other organisms

Policy information about studies involving animals; ARRIVE guidelines recommended for reporting animal research

Laboratory animals

B6;129P-Nsdl(fl/fl)<tm#Apet> x B6.Tg(Vavl-icre)A2Kio. More information regarding the generated Nsdl(fl/fl) mouse line can be made available by contacting the corresponding author or Prof. Antoine Peters (FMI, Basel, Switzerland). Mice were bred and maintained at the Animal Experimentation Facility of the Department of Biomedicine of the University of Basel. Animals are kept in groups of 5-6 in microisolator cages and provided continuously with sterile, water and chow pellets. Both female and male mice were used in equivalent numbers as the phenotype was not sex-biased.

Wild animals

This study did not involve the use of wild animals

Field-collected samples

This study did not involve field-collected samples

Ethics oversight

All experiments were done in adherence to Swiss laws for animal welfare and approved by the Swiss Cantonal Veterinary Office of Basel-Stadt, Switzerland (Permission 2087).

Note that full information on the approval of the study protocol must also be provided in the manuscript.

Human research participants

Policy information about studies involving human research participants

Population characteristics | *Describe the covariate-relevant population characteristics of the human research participants (e.g. age, gender, genotypic information, past and current diagnosis and treatment categories). If you filled out the behavioural & social sciences study design questions and have nothing to add here, write "See above."*

Recruitment | *Describe how participants were recruited. Outline any potential self-selection bias or other biases that may be present and how these are likely to impact results.*

Ethics oversight | *Identify the organization(s) that approved the study protocol.*

Note that full information on the approval of the study protocol must also be provided in the manuscript.

ChIP-seq

Data deposition

☒ Confirm that both raw and final processed data have been deposited in a public database such as GEO.

☒ Confirm that you have deposited or provided access to graph files (e.g. BED files) for the called peaks.

Data access links
May remain private before publication.

All sequencing data generated for this publication are in the GEO under accession number GSE136811.

Files in database submission

Processed files
KOl_Gatal_MM_mml0.bigWig
KOl_K27ac_MM_mml0.bigWig
KOl_Input_MM_mml0.bigWig
KO3_Gatal_MM_mm10.bigWig
KO3_K27ac_MM_mm10.bigWig
KO3_Input_MM_mm10.bigWig
WTMM_Gatal_mmlO.bigWig
WTMM_K27ac_mm10.bigWig
WTMM_input_mml0.bigWig
dSETMM_Gatal_mml0.bigWig
dSETM M_K27ac_mm 10.bigWig
dSETMM_input_mml0.bigWig
dSET24h_Gatal_mm10.bigWig
dSET24h_K27ac_mm10.bigWig
dSET _24_input_mm10.bigWig
WT24h_ Gatal_mm 10.bigWig
WT24h_K27ac_mm10.bigWig
WT_24_input_mm10.bigWig
WT_24h_GATAl_mm10.bigWig
WT_24h_H3K27ac_mm10.bigWig
WT_24h_lnput_mm10.bigWig
WT_MM_GATAl_mml0.bigWig
WT _MM_H3K27ac_mml0.bigWig
WT _MM_lnput_mml0.bigWig
SET_24h_GATAl_mml0.bigWig
SET _24h_H3K27ac_mml0.bigWig
SET _24h_lnput_mml0.bigWig
SET_MM_GATAl_mml0.bigWig
SET _MM_H3K27ac_mml0.bigWig
SET _MM_lnput_mml0.bigWig
KOl_K36met_MM_bowtie2_mml0_markDuplicates.bigWig
KO3_K36met_MM_bowtie2_mml0_markDuplicates.bigWig dSETMM_K36me3_bowtie2_mml0_markDuplicates.bigWig
WTMM_K36me3_bowtie2_mml0_markDuplicates.bigWig
WT24h_K36me3_bowtie2_mml0_markDuplicates.bigWig
dSET24h_K36me3_bowtie2_mml0_markDuplicates.bigWig
WT _24h_H3K36me 3 l_rep2_ bowtie 2_m m 10 _markDuplicates. bigWig WT _M M_H 3K36me31_rep2_bowtie2_mm 10 _markDuplicates.bigWig SET _24h_H3K36me3 l_rep2_ bowtie 2_m m 10 _ma rkDuplicates. bigWig SET _M M_H 3K36me31_rep2_bowtie2_mm 10 _markDuplicates.bigWig
RAW FILES
MM1_Gatal_S30_Rl_001.fastq.gz
MM1_K27ac_S31_R1_001.fastq.gz
MM1_Input_S40_Rl_001.fastq.gz
MM1_Input_S12_Rl_001.fastq.gz
MM3_Gatal_S37 _Rl_00l.fastq.gz
MM3_K27ac_S38_R1_001.fastq.gz
MM3_input_S43_Rl_001.fastq.gz

M M3 _in put_ S35 _Rl_ 001. fastq.gz
WTMM_Gatal_SS_Rl_00l.fastq.gz
WTMM_K27ac_S6_Rl_001.fastq.gz WTMM_input_S41_R1_001.fastq.gz WTMM_input_S13_R1_001.fastq.gz dSETM M_ Gatal_Sl 7 _Rl_00l.fastq.gz dSETMM_K27ac_S18_Rl_00l.fastq.gz dSETAM_input_S42_R1_001.fastq.gz
dSETAM_input_S14_Rl_00l.fastq.gz dSET24h_ Gata 1_ S24 _Rl_ 001. fastq.gz dSET24h_K2 7ac_ S25 _Rl_ 001. fastq.gz SET _24_input_S31_R1_001.fastq.gz
SET _24_input_S37 _Rl_00l.fastq.gz WT24h_Gatal_S12_R1_001.fastq.gz WT24h_K27ac_S13_R1_001.fastq.gz WT _24_input_S30_Rl_001.fastq.gz
WT _24_input_S36_Rl_001.fastq.gz 508_24h_GATA1_S27 _Rl_00l.fastq.gz 508 _24h_H 3K2 7ac_ S29 _Rl_ 001. fastq.gz 508_24h_lnput_S18_Rl_001.fastq.gz 508_MM_GATA1_S19_R1_001.fastq.gz 508_MM_H3K27ac_S21_R1_001.fastq.gz
508_MM_lnput_S33_R1_001.fastq.gz 553_24h_GATA1_S28_Rl_001.fastq.gz 553_24h_H3K27ac_S30_R1_001.fastq.gz
553_24h_lnput_S34_Rl_001.fastq.gz 553_MM_GATA1_S20_R1_001.fastq.gz
553_MM_H3K27ac_S22_Rl_001.fastq.gz
553_MM_lnput_S26_Rl_001.fastq.gz
MM1_K36me3_532_R1_001.fastq.gz
MM3_K36me3_539_Rl_001.fastq.gz
dSETMM_K36me3_519_R1_001.fastq.gz
WTMM_K36me3_57 _Rl_00l.fastq.gz
WT24h_K36me3_514_Rl_001.fastq.gz
dSET24h_K36me3_526_Rl_001.fastq.gz
508_24h_H3K36me3_531_Rl_001.fastq.gz
508_MM_H3K36me3_523_Rl_001.fastq.gz
553_24h_H3K36me3_532_Rl_001.fastq.gz
553_MM_H3K36me3_524_Rl_001.fastq.gz
MM1_Gatal_S30_R2_001.fastq.gz
MM1_K27ac_S31_R2_001.fastq.gz
MM1_1nput_S40_R2_001.fastq.gz
MM1_1nput_S12_R2_001.fastq.gz
MM3_ Gatal_S37 _R2_001.fastq.gz
MM3_K27ac_S38_R2_001.fastq.gz
MM3_input_S43_R2_001.fastq.gz
MM3_input_S35_R2_001.fastq.gz
WTMM_Gatal_S5_R2_001.fastq.gz
WTMM_K27ac_S6_R2_001.fastq.gz
WTMM_input_S41_R2_001.fastq.gz
WTMM_input_S13_R2_001.fastq.gz
dSETM M_ Gatal_Sl 7 _R2_001.fastq.gz
dSETMM_K27ac_S18_R2_001.fastq.gz
dSETAM_input_S42_R2_001.fastq.gz
dSETAM_input_S14_R2_001.fastq.gz
dSET24h_ Gata 1_ 524 _R2_ 001. fastq.gz
dSET24h_K2 7ac_ 525 _R2_ 001. fastq.gz
SET _24_input_S31_R2_001.fastq.gz
SET _24_input_S37 _R2_001.fastq.gz
WT24h_Gatal_S12_R2_001.fastq.gz
WT24h_K27ac_S13_R2_001.fastq.gz
WT _24_input_S30_R2_001.fastq.gz
WT _24_input_S36_R2_001.fastq.gz
508_24h_GATA1_527 _R2_001.fastq.gz
508 _24h_H 3K2 7ac_ 529 _R2_ 001. fastq.gz
508_24h_lnput_S18_R2_001.fastq.gz
508_MM_GATA1_519_R2_001.fastq.gz
508_MM_H3K27ac_S21_R2_001.fastq.gz
508 _M M_ln put_ 533 _R2_ 001. fastq.gz
553_24h_GATA1_528_R2_001.fastq.gz
553_24h_H3K27ac_S30_R2_001.fastq.gz
553_24h_lnput_S34_R2_001.fastq.gz
553_MM_GATA1_520_R2_001.fastq.gz
553_MM_H3K27ac_S22_R2_001.fastq.gz
553_MM_lnput_S26_R2_001.fastq.gz
MM1_K36me3_532_R2_001.fastq.gz
MM3_K36me3_539_R2_001.fastq.gz
dSETMM_K36me3_519_R2_001.fastq.gz
WTMM_K36me3_57 _R2_001.fastq.gz
WT24h_K36me3_514_R2_001.fastq.gz
dSET24h_K36me3_526_R2_001.fastq.gz
508_24h_H3K36me3_531_R2_001.fastq.gz
508_MM_H3K36me3_523_R2_001.fastq.gz
553_24h_H3K36me3_532_R2_001.fastq.gz
553_MM_H3K36me3_524_R2_001.fastq.gz

Genome browser session
(e.g. UCSC)

no longer applicable

Methodology

Replicates	Two biological replicates (independently transduced) were used in the Ch IP analysis and both replicates yielded similar results. Replicates where not merged, but used confirmatory.
Sequencing depth	paired-end, 50pb<br>KOl_Gatal_MM 63977172<br>KO1_K27ac_MM 54380640<br>KOl_lnput_MM 34532178<br>K03_Gatal_MM 49569330<br>K03_K27ac_MM 116764112<br>K03_1nput_MM 38477834<br>WTMM_Gatal 59147486<br>WTMM_K27ac 144820664<br>WTMM_input 4554936<br>dSETMM_Gatal 18660620<br>dSETMM_K27ac 246050158<br>dSETMM_input 25441868<br>dSET24h_Gatal 23091972<br>dSET24h_K27ac 180890620<br>dSET _24_input 21521454<br>WT24h_Gatal 19362446<br>WT24h_K27ac 222678524<br>WT_24_input 16574450<br>508_24h_GATA1 81157328<br>508_24h_H3K27ac 583776508<br>508_24h_lnput 57248836<br>508_MM_GATA1 145268294<br>508_MM_H3K27ac 601730828<br>508_MM_lnput 38169956<br>553_24h_GATA1 126073544<br>553_24h_H3K27ac 699226454<br>553_24h_lnput 33219906<br>553_MM_GATA1 129146144<br>553_MM_H3K27ac 660984404<br>553_MM_lnput 33488536
Antibodies	GATAl ab11862 10 ug per ChlP Abeam<br>H3K36me3 ab9050-11 10 ug per ChlP Abeam<br>H3K27ac ab4729 10 ug per ChlP Abeam
Peak calling parameters	Not applicable
Data quality	The data quality was assessed on several levels: (1) Read and alignment quality was evaluated using the qQCReport function of the bioconductor package QuasR. (2) Alignment statistics per sample measured the number of total, mapped, duplicated and chrM reads. (3) Sample correlation was calculated from a log-transformed counts per million (log-CPM) matrix which was generated from a genome-wide set of windows (window size 500bp) taking the top 500 most regulated windows into account. (4) IP strength was assessed by calculating the cumulative read density versus the cumulative genome size coverage. (5) For each sample, enrichment over input was evaluated using the log-CPM transfomed window counts. (6) GC bias was assessed by checking the number of observed over expected reads in GC bins from 0% to 100% across the same set of windows. (7) The frequency histogram of windows with up to 30 reads was used to assess the sequencing depth per sample. Reads with poor mapping quality were discarded from further analysis. Visual assessment of IGV tracks for antibody specificity and assessment of distribution of estimated fragment size. Re-sequencing was performed to ensure high number of reads.
Software	Reads were aligned with bowtie2 to the mouse genome (UCSC version mml0). The output was sorted and indexed with samtools. Duplicated reads were marked with pica rd. Coverage tracks per sample were generated by tiling the genome in 20bp windows and counting 5'end of reads per window using the function bamCount from the bioconductor package bamsignals. These window counts were exported in bigWig format using the bioconductor package rtracklayer.<br>R package csaw was used for differential binding analysis.

Flow Cytometry

Plots

Confirm that:

☐ The axis labels state the marker and fluorochrome used (e.g. CD4-FITC).

☒ The axis scales are clearly visible. Include numbers along axes only for bottom left plot of group (a 'group' is an analysis of identical markers).

☒ All plots are contour plots with outliers or pseudocolor plots.

☒ A numerical value for number of cells or percentage (with statistics) is provided.

Methodology

Sample preparation	A detailed protocol for cell preparation for FACS analysis and sorting is described in the materials and method sections. In summary, single haematopoietic cells either from human CD34+ HSCP or mouse bone marrow were treated with red blood lysis and then either analyzed directly or depleted for lineage-marker expressing cells prior to analysis. Fetal liver cells were isolated from E13, E14.5, E1 7 and E19 fetuses and either analyzed directly or following Red blood lysis. Cells were always washed twice with PBS (2X) prior to 30 min incubation for staining in buffer (5mM EDTA, 0.5% BSA, PBS) at 4 C. Before analysis, the cells were washed with buffer (5mM EDTA, 0.5% BSA, PBS) and then resuspended in buffer + DAPI before analysis.
Instrument	FACS Diva or LSR II Fortessa (BD, Biosciences) and BD cytoflex
Software	FloJo-Software (Version 10.0.8 or 10.2)
Cell population abundance	Post sorting purity was always <95% and is determined (when possible) by reanalysis on LSR II Fortessa machine
Gating strategy	Gating Strategy as the following: Cells population (FSC-A vs. SSC-A), single cells population (FSC-A vs. FSC-H), Live/GFP+ population (DAPI vs. FSC-H or DAPI vs. GFP) and gated according antigen/fluorescence used according to unstained control and positive and negative beads (Thermofisher Ref. A10344). A detailed gating strategy is provided in Supplementary Figure 8.

☒ Tick this box to confirm that a figure exemplifying the gating strategy is provided in the Supplementary Information.
